# A General and Scalable Method toward Enantioenriched C2-Substituted Azetidines Using Chiral *tert*-Butanesulfinamides

**DOI:** 10.1021/acs.joc.4c01908

**Published:** 2024-09-30

**Authors:** Daniel Zelch, Christopher M. Russo, Kirsten J. Ruud, Matthew C. O’Reilly

**Affiliations:** † Department of Chemistry, Villanova University, Villanova, Pennsylvania 19085, United States; ‡ Department of Chemistry and Biotechnology, University of Wisconsin−River Falls, River Falls, Wisconsin 54022, United States

## Abstract

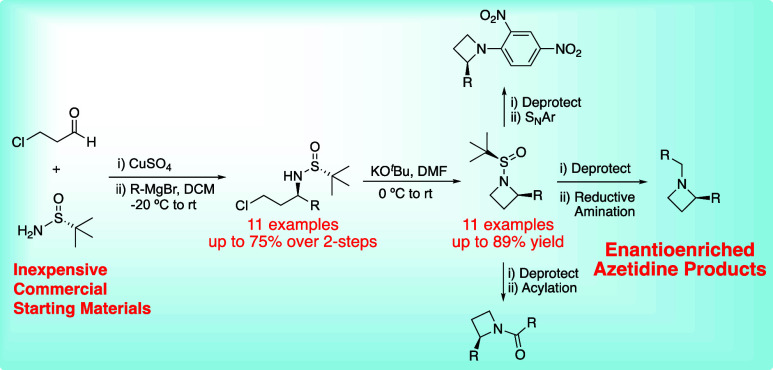

Diverse ranges of chiral nitrogen-containing heterocycles serve as a molecular toolbox for modulating a wide array of biological processes, but enantioenriched production of smaller chiral heterocycles is a bottleneck. There is a lack of general approaches for the stereoselective preparation of chiral 4-membered monocyclic C2-substituted azetidines, where many routes to different substitution types are possible, but no simple and common approach exists. To bridge this gap, inexpensive and widely available chiral *tert*-butanesulfinamides are harnessed for chiral induction with 1,3-bis-electrophilic 3-chloropropanal, providing a three-step approach to C2-substituted azetidines with aryl, vinyl, allyl, branched alkyl, and linear alkyl substituents. Eleven azetidine products are produced, and the approach is shown to be effective on a gram-scale with a single purification of the protected azetidine product in 44% yield over three steps in an 85:15 diastereomeric ratio. In most cases, the diastereomers are separable using normal phase chromatography, often resulting in previously elusive enantiopure azetidine products. Protected azetidines were shown to undergo rapid and efficient sulfinamide cleavage, producing an azetidine hydrochloride salt that was subjected to derivatization reactions, highlighting the method’s applicability to medicinal chemistry approaches.

## Introduction

Heterocycles are key components of more than 85% of biologically active compounds, and nitrogen-containing heterocycles are present in approximately 60% of today’s FDA approved drugs, making their ease of synthesis critical to the development of life-saving therapeutics.^1,2^ This includes saturated nitrogen-containing heterocycles, with the 5- and 6-membered variants being most common among drugs (e.g., piperidines, piperazines, morpholines, and pyrrolidines).^1−5^ These molecules are often considered privileged structures,^6,7^ a term that implies their ability to bind a broad range of biological targets while also being exquisitely selective for those targets if adorned with ideal chiral substitution.

**Figure 1:**
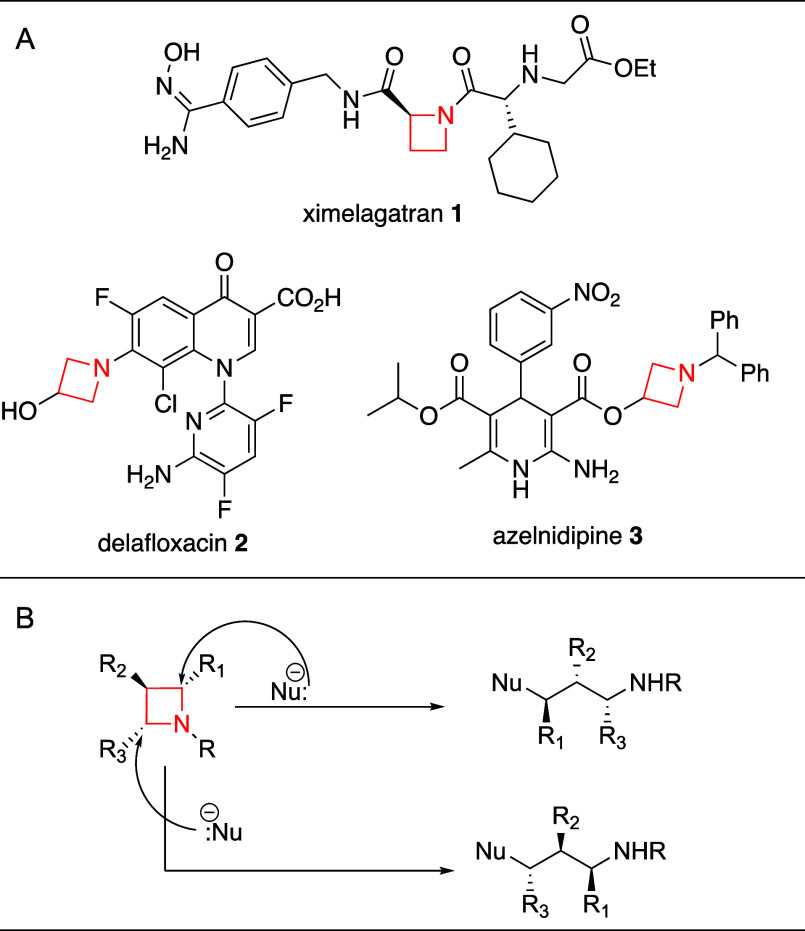
(A) FDA-approved therapeutics containing azetidines. (B) Ring opening can be regioselective and facilitate chirality transfer.

Azetidines, saturated 4-membered nitrogen heterocycles, are less explored synthetically and medicinally, which is demonstrated by the fact that they are found in less than 1% of today’s FDA approved drugs containing nitrogen heterocycles.^8−10^ However, when properly substituted, they can potently bind a broad array of biological targets due in part to the rigidity of their four-membered ring.^11^ While rare, various FDA-approved pharmaceuticals contain azetidines (Figure 1A), including anticoagulant ximelagatran **1**, antibiotic delafloxacin **2**, and calcium channel blocker azelnidipine **3**. Most of the azetidine-containing therapeutics are C3-substituted, making the azetidine motif achiral (as in **2** and **3**). Indeed, three additional FDA approved pharmaceuticals containing monocyclic azetidines were approved from 2013 to 2023, and the azetidines in each case were C3-substituted.^12^ Chiral C2-substituted azetidine motifs are less common (as in **1**), and they are almost exclusively carboxylic acid derivatives, and their syntheses are often not described, including a lack of literature disclosure for the sourcing or synthesis of the chiral azetidine of ximelagatran **1**. Beyond the use of azetidines as components of biologically active molecules, substituted chiral azetidines are useful synthetic intermediates, as ring opening reactions of this strained ring system can provide functionalized chiral products (Figure 1B).^13^ Despite the clear usefulness of azetidines, and significant recent works by the Schindler,^14,15^ Gaunt,^16^ Aggarwal,^17^ and Kürti^18^ Groups toward novel preparations of racemic azetidines with various substitution patterns, general methods toward the stereoselective preparation of chiral C2-substituted azetidines are lacking in efficiency, generalizability, and scalability.

**Scheme 1:**
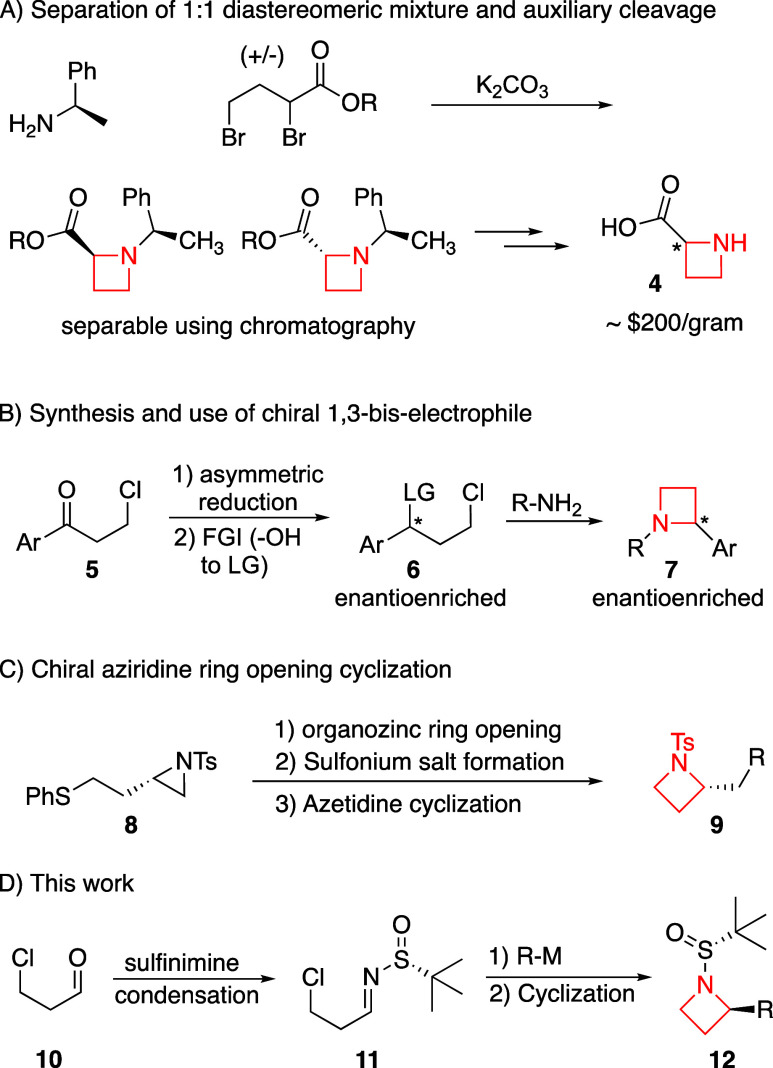
C2-Substituted Azetidine Syntheses

The most common approach toward chiral C2-subtituted azetidines involves the acquisition or synthesis of chiral azetidine-2-carboxylic acids **4**. These are commercially available (∼$200/gram) and have two published syntheses producing 1:1 diastereomeric azetidine-2-ester mixtures that are separated using chromatography (Scheme 1A).^19,20^ Once azetidine **4** is prepared or purchased, further functionalization can occur, and a variety of groups have developed chemistry that can be performed on these accessible chiral azetidines.^21−24^ Secondary approaches typically produce an enantioenriched electrophile that transfers its chirality to the final azetidine through a stereospecific reaction. Toward that end, a classic option involves the synthesis of a chiral 1,3-bis-electrophilic motif, which can undergo bis-substitution with an amine to produce a chiral azetidine product. The most common utilization of this involves the preparation of aryl ketone **5**, which can undergo an enantioselective reduction to a chiral alcohol (commonly the CBS reduction) followed by formation of a leaving group (−LG) via sulfonylation or stereospecific substitution with a halide, forming **6**. Bis-substitution then provides enantioenriched azetidine **7**, commonly in around 80% ee (Scheme 1B).^25−28^ This approach is less useful when the ketone’s substituents are similar in size, as the asymmetric reduction is most selective when the substituents have different steric footprints. This works well when the desired C2-substituent is an arene (e.g., **5**), and these types of starting materials are generally available using Friedel–Crafts acylation chemistry,^29^ but this method is less useful if C2-alkyl substitution is desired. A final noteworthy method providing general access to monocyclic C2-substituted azetidines involves the enantioselective preparation of an aziridine tethered to a phenyl sulfide **8**, which is synthesized in 6 steps from a commercially available enantiopure homoserine lactone (∼$33/gram).^30^ Organozinc addition to **8** opens the aziridine, Meerwein’s Salt (Me_3_OBF_4_) is used to alkylate the sulfide, forming a sulfonium salt leaving group, which is displaced during cyclization to form azetidine **9** (Scheme 1C). This three-step sequence was high yielding, producing the desired azetidines in 63–82% yield with no loss in enantioselectivity. Downsides include the six steps required to synthesize the starting materials, only ethyl-linked R-groups were disclosed (although a methylene linkage, as shown in Scheme 1C, may theoretically be possible depending on the choice of organozinc reagent), the chemistry cannot provide direct aryl or branched alkyl substitution off the azetidine C2-position, and the *N*-tosyl protecting group was never removed, indicating it may be a challenge. Further, only the (*S*)-enantiomer of the homoserine lactone starting material is commercially available, limiting preparation to a single stereoisomer of the product azetidine. Beyond these methods for asymmetric azetidine preparation, recent works in the Evano, Morken, Du Bois, and Iwabuchi Laboratories have provided new approaches for racemic syntheses of C2-substituted azetidines.^23,31−35^

The available methods for the preparation of enantioenriched C2-substituted azetidines could be described as a patchwork, where no one method provides access to a broad range of azetidines, and all approaches require many steps from commercial materials. To address this, we have developed chemistry combining achiral starting materials with the Ellman *tert-*butanesulfinamide chiral auxiliary, as it is inexpensive (∼$2/gram), both enantiomers are broadly available, it provides strong chiral induction, and it acts as a protecting group that can be easily cleaved after cyclization to the azetidine. Specifically, 1,3-bis electrophile 3-chloropropanal **10** undergoes condensation with the auxiliary to form sulfinimine **11**. Organometallic addition and intramolecular chloride substitution provides azetidine **12** in high diastereoselectivity, which provides enantioenriched C2-substituted azetidines following protecting group cleavage (Scheme 1D). Importantly, our chemistry provides access to aryl, vinyl, allyl, alkyl, and branched alkyl substitution at the C2-position with high stereoselectivity, and the azetidine is formed in only three-steps from the achiral aldehyde starting material.

## Results and Discussion

**Scheme 2:**
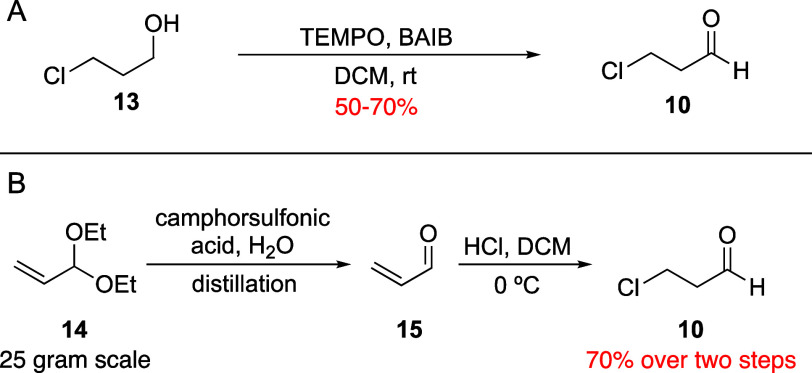
Preparation of 3-Chloropropanal

The proposed azetidine synthesis relies on easy access to 3-chloropropanal **10**, and we were concerned that this starting material may be unstable, as elimination of the leaving group would generate acrolein, a thermodynamic sink with a stable α,β-unsaturated aldehyde. Despite this, we noted that oxidation to **10** was reported via pyridinium chlorochromate (PCC)^36^ or manganese dioxide (MnO_2_)^37^ in the patent literature. However, in our hands, PCC led to the desired oxidation accompanied by considerable decomposition of **10**, and MnO_2_ was incapable of facilitating the oxidation as described. Beyond those reagents, **10,** and similar 3-bromopropanal, had additional preparations, including either a catalytic TEMPO oxidation with [bis(acetoxy)iodo]benzene (BAIB) as a stoichiometric oxidant^38,39^ or the addition of HCl to acrolein (Scheme 2).^40,41^ While ultimately these preparations were successful, complications arose during their synthesis. Oxidation of 3-chloropropanol **13** was attempted, and while analysis of a reaction aliquot showed a successful oxidation, concentration of aldehyde **10** after purification led to decomposition, likely via chloride elimination and oligomerization of the resulting acrolein. While these issues were not described in the literature,^39^ we considered that storage of **10** in solution after the oxidation reaction and resulting column may shield it from decomposition. Therefore, the oxidation was repeated, and the purified product was stored as a solution in dichloromethane (DCM, 0.5–1.0 M), which allowed preparation of **10** in yields between 50 and 70% (Scheme 2A). The yield variability is likely due to modest decomposition occurring during the workup. As the oxidation approach required a stoichiometric oxidant, we considered that the addition of HCl to acrolein **15** may be more atom economical.^40,41^ However, this option requires easy access to large quantities of acrolein, which is no longer commercially available on scale. Therefore, acrolein diethyl acetal **14** is an inexpensive and broadly available alternative, and a two-step approach could then provide access to **10**. First, following a literature preparation that cleaved **14**’s acetal in quantitative yield on a 168-g scale,^42^ the acetal was hydrolyzed by mixing **14** with camphorsulfonic acid and water with simultaneous distillation of the acrolein product. This produced a secondary challenge, as the distillation produces a mixture of acrolein **15**, water, and ethanol, and the subsequent reaction requires anhydrous conditions. Therefore, the acrolein distillate was extracted into dichloromethane, allowing the ethanol and water to be removed via multiple aqueous washes. In the following reaction, HCl gas was bubbled through the DCM solution, where complete conversion was observed after periodic ^1^H NMR analysis of reaction aliquots. This two-step approach was more scalable, as it required no chromatography, and it was performed on a 25-g scale, producing **10** in 70% yield (Scheme 2B). As the second reaction of **15** to **10** appeared to be quantitative, with no workup or purification steps to optimize, it is likely that the yield from **14** to **10** could have been improved by optimizing the extraction sequence of **15**. Either way, both methods for the preparation of 3-chloropropanal **10** were effective, facilitating access to this precursor in our proposed azetidine synthesis.

**Scheme 3:**
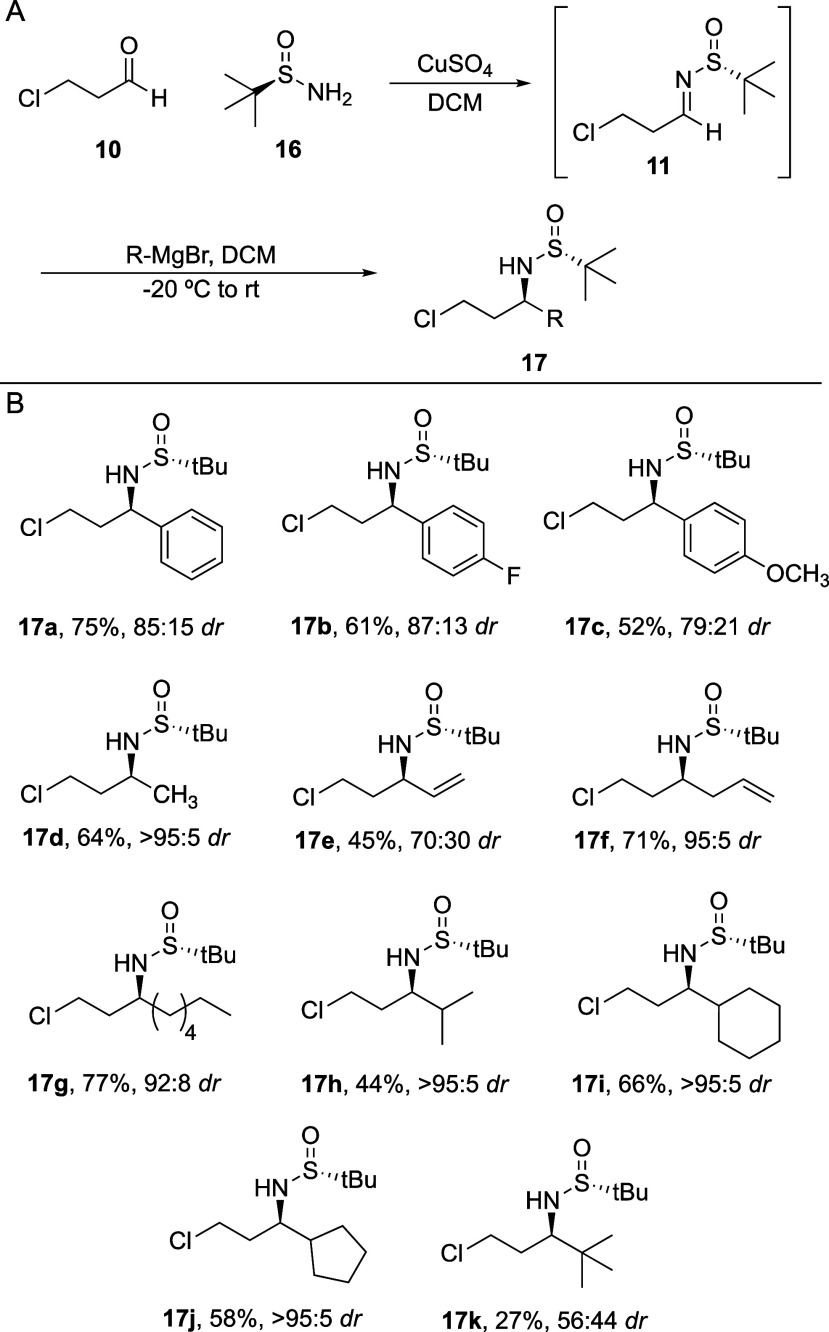
Sulfinimine Synthesis and Addition

With 3-chloropropanal **10** in hand, condensation with *tert-*butanesulfinamides was explored. With standard aldehydes, the condensation is facilitated by a Lewis acidic desiccant, and the resulting sulfinimine is bench stable on large scale.^43−47^ Therefore, we attempted synthesis by combining aldehyde **10** with (*R*)-*tert-*butanesulfinamide **16** and copper(II) sulfate in dichloromethane, and a ^1^H NMR of an aliquot of the reaction mixture showed conversion to the sulfinimine **11**. After workup and purification of **11**, however, complete decomposition occurred, demonstrated by NMR evidence of chloride elimination and isolation of additional insoluble polymeric material, indicating that **11** is too sensitive for concentration and long-term storage (Scheme 3A). To circumvent this issue, we considered that telescoping the imine formation together with organometallic addition may form a more stable product, as elimination of the chloride would no longer generate a conjugated olefin. Therefore, after imine formation, filtration was performed to remove the copper salts and other insoluble impurities, eluting with dichloromethane to double the reaction volume. At that point, the reaction was cooled to −46 °C in a dry ice/acetonitrile bath, and a Grignard reagent was added. This proceeded in good yield and *dr*, and further optimization indicated that this one-pot method was successful even without the filtration step, where the Grignard is added dropwise directly to the imine after cooling, demonstrating that the organometallic addition can occur in the presence of copper(II) sulfate. Further, comparing the diastereoselectivity of the addition at a warmer temperature (NaCl/ice bath, −20 °C) indicated that minimal loss in diastereoselectivity was observed, and the reaction was performed at −20 °C from this point onward to minimize acetonitrile waste and the use of dry ice.

To explore the scope of this method, the reaction sequence was performed with a variety of commercial Grignard reagents, providing a variety of chiral chlorosulfinamide products in yields up to 77% over two steps (Scheme 3B). Aryl Grignard reagents proceeded in 52–75% yield, with diastereoselectivity ranging from around 87:13 to 79:21 (**17a**–**c**). Unbranched methyl, vinyl, allyl, and hexyl Grignard reagents provided a similar range of yields, and the methyl, allyl, and hexyl nucleophiles added with excellent diastereoselectivity (**17d**–**g**, >95:5, 95:5, and 92:8 respectively). Surprisingly, vinyl Grignard gave a significant reduction in yield (45%) and *dr* (70:30). This may be improved if the reaction is run at a cooler temperature, but vinyl Grignard is known to have quality issues,^48^ where decomposition to a mixture of magnesium hydride and oligomeric alkenyl magnesium bromides could have led to decomposition. We noted our commercial vinyl magnesium bromide turned a deep red color indicative of this decomposition, and highest yields occurred when the newly acquired reagent had an amber color. Despite the issues with the vinyl Grignard, incorporation of an alkene in the vinyl and allyl products allows an additional handle that could be useful for derivatization of the final azetidine products. Importantly, branched Grignards, which would produce the more elusive branched azetidines following cyclization, continued to produce chlorosulfinamide products in similar two-step yields (44–66%) with excellent diastereocontrol (∼95:5) including the isopropyl, cyclohexyl, and cyclopentyl products (**17h**–**j**). Lastly, the *tert*-butyl Grignard addition caused some issues. Our standard conditions kept the reaction at −20 °C for 1.5 h, then allowed to warm to room temperature for an additional 30 min prior to quenching, but none of the *tert*-butyl Grignard addition product was noted with these conditions. To some degree, this reaction could be expected to occur at a slower rate due to the increased steric footprint of the *tert*-butyl group. Therefore, the reaction was repeated, but was left overnight to stir at room temperature, and this led to isolation of product, albeit in a modest two-step yield of 27% with almost no diastereocontrol (**17k**), demonstrating that *tert*-butyl substitution can be accomplished, but with significant challenges.

With the chlorosulfinamides in hand, attention turned to cyclization. Initial conditions were based on a similar cyclization that produced chiral 2-aryl pyrrolidines, which was performed with 3 equiv of potassium hydroxide in 1:1 THF/H_2_O at reflux.^49^ When this was applied to cyclization of chlorosulfinamide **17a**, a 30% yield of azetidine accompanied by decomposition was observed (Table 1). When the same reaction conditions were used at lower temperatures, a 9% yield with 54% recovered starting material was observed, indicating that a significant amount of decomposition was occurring even at the lower temperatures. Next, solvents DMF, THF, and DCM were combined with organic bases potassium *tert*-butoxide (KO*^t^*Bu) or lithium hexamethyldisilazane (LHMDS), and these led to higher isolated yields of the azetidine and less decomposition. The initially most promising condition was KO*^t^*Bu in DMF (0.025 M), leading to a 78% isolated yield, but many of the conditions gave similar yields. Further, variability was noted within the individual conditions. For instance, when entry 3 was repeated a second time, a 72% yield was obtained, and it was later found that the product azetidine **18a** was volatile. Specifically, after product concentration via rotary evaporation, product material was often transferred to a scintillation vial and concentrated under a stream of air, and this was found to lead to loss of product mass. Once this was realized, it was noted that the yield differences in entries 3–7 could have been related to variability with the amount of time the product material was subjected to a stream of air. To address this, as many of the proposed azetidines were likely to be even more volatile than **18a**, we found that the extraction and subsequent column chromatography could be performed exclusively with diethyl ether, a low boiling solvent, which would allow product concentration without subjecting the material to high vacuum for long periods. To examine this, the reaction was performed with KO*^t^*Bu in DMF (0.1 M) with diethyl ether extractions and chromatography, and this led to our highest isolated yield of **18a**, 89%.

**Table 1:**
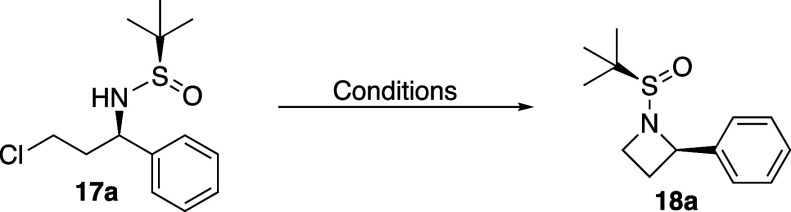
Optimization of Azetidine Cyclization

Entry	Base	Eq.	Solvent	Temperature	Yield^a^
1	KOH	3	THF/H_2_O, 0.1 M	reflux	30%
2	KOH	3	THF/H_2_O, 0.1 M	0–>rt	9%^b^
3	KO*^t^*Bu	3	DMF, 0.025 M	0–>rt	78%
4	LiHMDS	2	4:1 THF/DCM 0.02 M	0–>rt	62%
5	KO*^t^*Bu	3	4:1 THF/DCM 0.02 M	0–>rt	72%
6	KO*^t^*Bu	3	DMF, 0.1 M	0–>rt	67%
7	LiHMDS	3	DMF, 0.025 M	0–>rt	68%
8	KO*^t^*Bu	1.5	DMF, 0.1 M	0–>rt	89%^c^

a Isolated yields.

b Recovered 54% of the starting material.

c Workup and column chromatography performed with exclusively diethyl ether to minimize product loss.

**Scheme 4:**
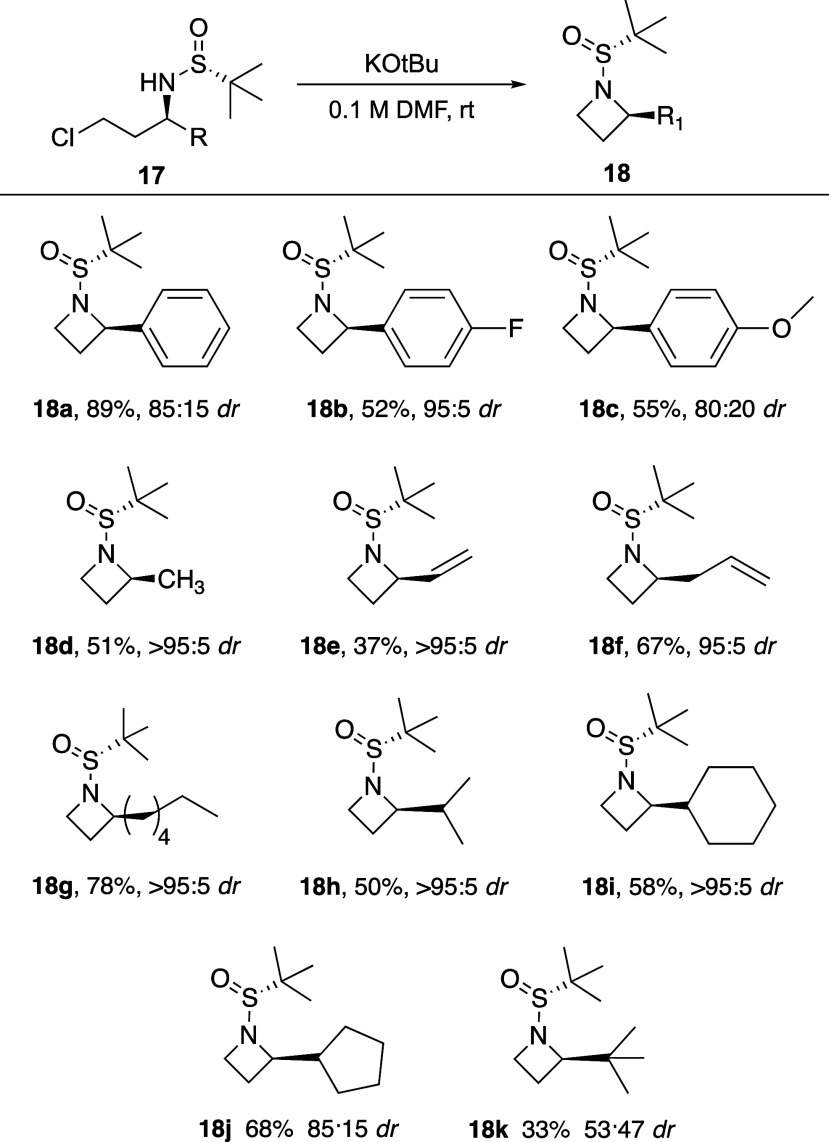
Azetidine Cyclization Scope

To explore the scope of the cyclization, these reaction conditions were applied to all chlorosulfinamides previously synthesized, and all reactions provided azetidine products **18** in yields from 89 to 33% (Scheme 4). Diastereomeric ratio was retained from the chlorosulfinamide **17** to the azetidine products **18**, with any changes from Scheme 3 being due to full or partial separation of the diastereomers during isolation of the chlorosulfinamide products. For example, vinyl product **17e** was produced in 70:30 *dr*, but isolation of **17e** separated the major and minor diastereomers. Therefore, during cyclization, a diastereoenriched sample of **17e** was chosen as the starting material (>95:5), which produced azetidine **18e** in modest yield while retaining the >95:5 *dr* from the starting materials. In general, it was often challenging to separate chlorosulfinamide diastereomers using normal phase chromatography (e.g., no separation of **17a** diastereomers), but diastereomeric azetidine products **18** generally had larger differences in retention, frequently allowing for their separation. While we had seen the volatility of **18a** previously, we noted that other azetidines, especially short chain azetidines (**18d**–**f, h, k**), could be even more volatile, and there is a chance some of the low yields could be due to product loss related to that volatility. Additionally, while most azetidine products had significant benchtop stability, decomposition was noted in some cases. Specifically, an NMR sample of azetidine **18c** was shown to be pure after chromatography, but after leaving the material in chloroform-D overnight, 10–20% decomposition was noted the following morning. This lack of stability was never seen for azetidine **18a**, which appeared to have indefinite room temperature stability. This indicates that the electron donating methoxy group on **18c** likely activates the azetidine for ring opening.

**Scheme 5:**
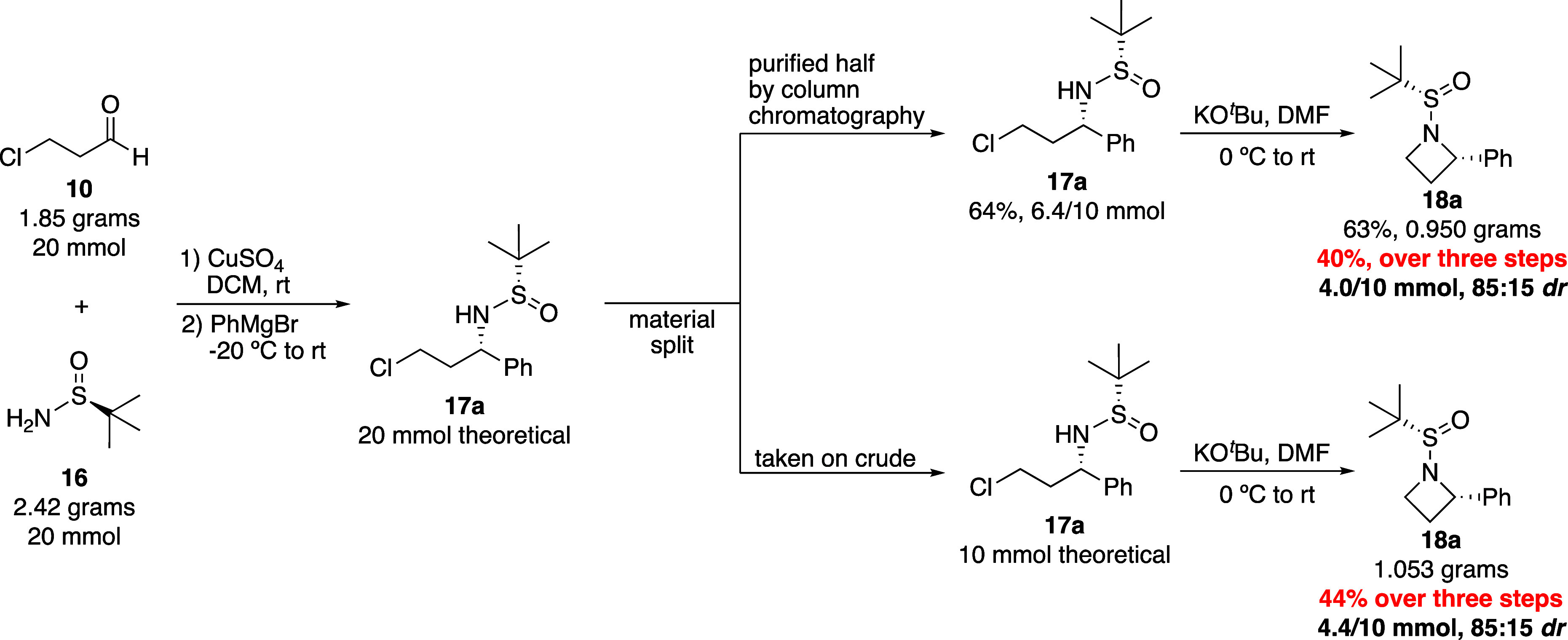
Gram Scale Azetidine Synthesis

To demonstrate the scalability of the method, which would be important in a medicinal chemistry context for those wanting to synthesize larger quantities of a given azetidine to later derivatize, synthesis of azetidine **18a** was attempted on a 20 mmol scale, with a theoretical yield of 4.786 g (Scheme 5). On larger scale, the condensation and Grignard addition worked well, producing crude sulfinamide **17a** that looked quite clean when examined by ^1^H NMR. To determine whether purification was necessary at this stage, the crude material was split into equal parts: half was purified by column chromatography and the other half was used directly in a cyclization reaction without prior purification. Comparing these parallel routes allowed us to determine the importance of chlorosulfinamide **17a** purity in both the efficiency of the subsequent cyclization and the overall three-step yield. The purification step provided a 64% yield of **17a** over two steps (1.754 g, 6.4/10 mmol), and subsequent cyclization of this pure material provided 0.950 g of azetidine **18a**, which was a combined 40% yield over three steps (4.0/10 mmol). When **17a** was used without purification, the cyclization produced 1.053 g of azetidine **18a**, or 44% over three steps (4.4/10 mmol), slightly higher than when **17a** was purified (Scheme 5). This demonstrated that crude chlorosulfinamides, such as **17a**, could be cyclized without negatively impacting yields, allowing for the preparation of the protected azetidine products with only one purification step. The 85:15 *dr* indicated below was for the isolated material, where both diastereomers could be separated with a single normal-phase column, and this value matched the *dr* calculated from the crude NMR data during our substrate scope (Scheme 4). Yield suffered on larger scale, where the three-step sequence produced a 44% yield compared to 66% when it was performed on small scale. While this was slightly disappointing, the chemistry held up on larger scale, easily providing gram scale quantities of diastereoenriched azetidines after a single chromatographic purification from inexpensive and readily available starting materials.

**Scheme 6:**
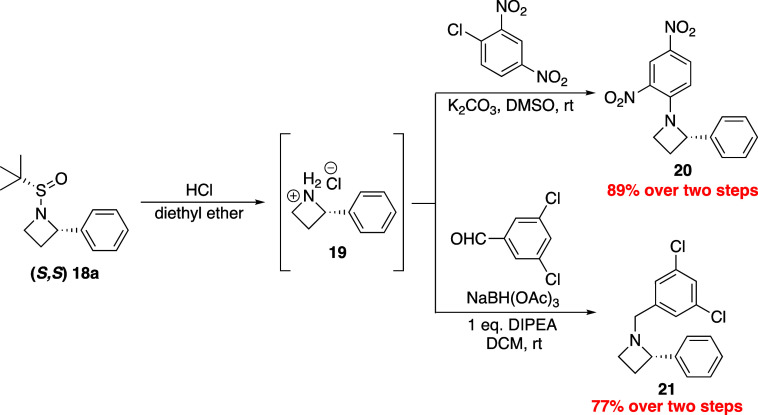
Azetidine Deprotection and Derivatization

With streamlined access to sulfinyl azetidines, our attention turned to demonstrating that our products could be easily deprotected and derivatized. First, we attempted sulfinamide cleavage using the typical literature conditions, which involves treating the sulfinamide with anhydrous HCl in methanol.^43−45,47^ Unfortunately, when these conditions were applied to azetidine **18a**, it led to deprotection with concomitant partial decomposition. This was not all too surprising, as the C2-position of 2-phenylazetidine has increased electrophilicity due to benzene’s ability to stabilize cationic character, making it presumably more reactive than an alkyl substituted azetidine. A solvent change to diethyl ether suppressed any decomposition, leading to deprotection in quantitative yield (Scheme 6). Further, this was an operationally simple reaction, with the azetidine hydrochloride salt **19** precipitating as a solid, allowing for isolation by decantation and/or filtration. When the azetidine **18** diastereomers were separable, as was the case for most derivatives including **18a**, deprotection led to enantiopure C2-substituted monocyclic azetidines, which could then produce enantiopure azetidine derivatives. To demonstrate this, telescoping deprotection reactions to derivatization reactions often used by medicinal chemists was pursued. First, nucleophilic aromatic substitution was performed with 1-chloro-2,4-dinitrobenzene, which smoothly produced *N-*aryl-2-phenyl azetidine **20** in 89% yield over two steps. Additionally, reductive amination with 3,5-dichlorobenzaldehyde in the presence of sodium triacetoxyborohydride was performed, producing the functionalized product **21** in 77% yield over two steps.

**Scheme 7:**
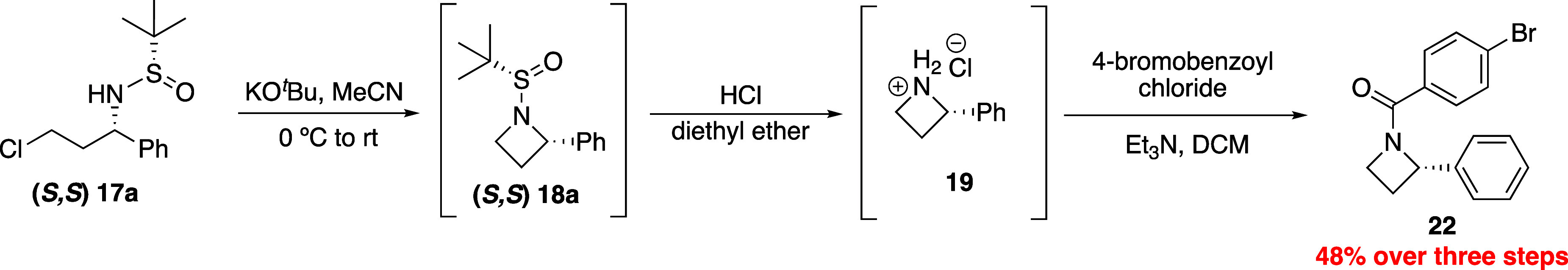
One-Pot Cyclization, Deprotection, and Acylation Sequence

In the case that an azetidine sulfinamide may have significant volatility, as we expected the short chain compounds to have (e.g., **18d**–**f**), we postulated that cyclization could be performed without concentrating the reaction mixture, where subsequent deprotection and further functionalization could produce a less volatile product, stitching multiple reactions together. Further, derivatization of azetidines via amide formation could be an important strategy used by medicinal chemists, so we ventured to perform a cyclization, deprotection, and acylation reaction sequence to produce azetidine amides. One initial issue with this one-pot approach involved DMF providing enhanced solubility of the azetidine hydrochloride salt **19**, which interfered with our ability to purify the salt with a simple filtration. As other solvents had formerly been found to promote the cyclization, we attempted the reaction in a different solvent that we considered less likely to solubilize **19** when mixed with diethyl ether. Therefore, chlorosulfinamide **17a** was subjected to cyclization with acetonitrile as the solvent, and instead of isolating the azetidine sulfinamide **18a**, the reaction was diluted with diethyl ether to decrease the solubility of undesired ionic salts (Scheme 7). The crude mixture of soluble azetidine **18a** and insoluble salts was passed through a paper filter, eluting with additional diethyl ether, and anhydrous HCl was added directly to that filtrate, effectively cleaving the sulfinamide, providing azetidine hydrochloride **19**. This mixture was further filtered to remove the diethyl ether and other organic-soluble byproducts, and the insoluble materials, including **19,** were dissolved in DCM. Acylation was promoted with 4-bromobenzoyl chloride and triethylamine, producing the azetidine amide **22** in 48% yield over three steps. This three-step sequence included only one, terminal liquid–liquid extraction, allowing for the isolation of **18a** and **19** by simple filtration steps, and it could be applied to generate azetidine amide libraries.

## Conclusions

Herein, we have disclosed a general and scalable approach to chiral C2-substituted monocyclic azetidines in good yields and diastereoselectivity. The three-step method starts from inexpensive starting materials that produce their diastereocontrol using chiral *tert*-butanesulfinamides, where either stereochemistry of the products can be produced using either the (*R*)- or (*S*)-sulfinamide reactants. The protected azetidine product diastereomers can be separated with normal phase chromatography, and subsequent azetidine deprotection provides enantioenriched monocyclic C2-substituted azetidine products. The chemistry provides a platform to produce aryl, vinyl, allyl, branched alkyl, and linear alkyl substitution in the azetidine’s 2-position, which represents an elusive series of substituent types previously inaccessible via a single method to produce optically active azetidines. Further, we demonstrate that azetidine deprotection is possible and operationally simple, and that sulfinamide cleavage can be coupled to further azetidine functionalization via traditional amine functionalization chemistries.

## Data Availability

The data underlying this study are available in the published article and its Supporting Information.

## Supporting Information Available

General synthesis considerations; description of *dr* determination; full details of chemical synthesis including full characterization; NMR spectra of all final compounds and intermediates (PDF)
