# Supplementary material for: A General and Scalable Method toward Enantioenriched C2-Substituted Azetidines Using Chiral tert-Butanesulfinamides
Source: J Org Chem. 2024 Sep 30;89(20):15137–44. doi: 10.1021/acs.joc.4c01908 (PMC11494643; doi:10.1021/acs.joc.4c01908)
Supplement: Supplementary file 1 — jo4c01908_si_001.pdf [file jo4c01908_si_001.pdf]

A general and scalable method toward enantioenriched C2-substituted azetidines using chiral *tert*-butanesulfinamides

Daniel Zelch,[a] Christopher M. Russo,[a] Kirsten J. Ruud,[b] and Matthew C. O'Reilly[a,*]

[a]Department of Chemistry, Villanova University, Villanova, Pennsylvania 19085, United States

[b]Department of Chemistry and Biotechnology, University of Wisconsin–River Falls, River Falls, Wisconsin 54022, United States. Present Address: University of California–Irvine, Irvine, California, 92697, United States

Corresponding Author Information

*Corresponding author: Matthew C. O'Reilly – Department of Chemistry, Villanova University. ORCID: 0000-0002-9175-4179. Email: matthew.oreilly@villanova.edu

Supporting Information

1. Details of chemical synthesis and characterization – *page S2*
    a. General Synthesis Considerations – *page S2*
    b. 3-chloropropanal Synthesis Methods – *page S3*
    c. Chlorosulfinamide **17** Synthesis – *page S6*
    d. Protected Azetidine **18** Synthesis – *page S13*
    e. Azetidine Derivatization (**19-22**) – *page S20*
    f. Determination of diastereoselectivity – page S25
2. NMR spectra – *page S30*
3. Literature Cited – *page 85*

General Synthesis Considerations

All reagents were purchased from commercial suppliers and were used without additional purification. Thin-layer chromatography (TLC) was performed on TLC Silica gel 60 F254 from Supelco. Visualization was accomplished via UV light, and/or the use of iodine or potassium permanganate staining. Flash chromatography was performed using normal phase Silica RediSep Silver Rf flash columns on a CombiFlash Rf automated flash chromatography system. All ^{1}H and ^{13}C NMR spectra were recorded on a 400 MHz or 500 MHz JEOL spectrometer. Chemical shifts are reported in ppm relative to residual solvent peaks as an internal standard set to δ 7.26 and δ 77.16 (CDCl3). Data are reported as follows: chemical shift, multiplicity (s = singlet, d = doublet, t = triplet, q = quartet, p = pentet, sx = sextet, sp = septet, br = broad, dd = doublet of doublets, dq = doublet of quartets, td = triplet of doublets, pd = pentet of doublets, m = multiplet), coupling constant (Hz), integration. High resolution mass spectra (HRMS) were recorded on an AB Sciex 5600 + TripleTOF using electrospray ionization in positive mode. Optical rotation was acquired using a PerkinElmer Instruments Model 341 Polarimeter.

3-chloropropanal synthesis methods

Oxidation Approach:

3-chloropropanal **10** was synthesized via oxidation as detailed below. This details the reaction on a 40 mmol scale where the product was produced in 64% yield. The reaction was run at a variety of scales, and typical yields were between 50-70%

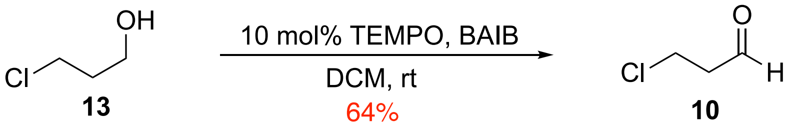

The reaction was based on a report in *Journal of Organic Chemistry*[1] and *Tetrahedron Letters*.[2] To a round-bottomed flask equipped with a magnetic stir bar was added 3-chloropropanol (3.780 grams, 39.98 mmol) and dichloromethane (40 mL), and the flask was placed in a room temperature water bath. To the mixture was added 2,2,6,6-tetramethylpiperidinyloxyl (TEMPO) (0.625 grams, 3.99 mmol) followed by [bis(acetoxy)iodo]benzene (BAIB) (14.167 grams, 43.98 mmol). After stirring for two hours, the reaction was diluted with dichloromethane (60 mL) and was transferred to a separatory funnel that contained saturated sodium thiosulfate (100 mL). After shaking, the organic layer was removed, and the aqueous layer was further extracted with dichloromethane (2 X 50 mL). The organic material (>200 mL) was transferred back to an empty separatory funnel, and it was washed with saturated brine (2 X 75 mL). The organic layer was then dried with sodium sulfate, filtered, and concentrated using a rotary evaporator. To prevent decomposition or loss of the volatile aldehyde, the rotovap bath was set to 25 °C and the vacuum was at 90 torr at its maximum vacuum pressure. At this point, a significant volume of liquid is present, and ^{1}H NMR indicates it is a mixture of the desired product and iodobenzene (from BAIB). To purify **10**, flash chromatography was performed using 100% dichloromethane as the eluant. This led to separation of iodobenzene (R_f = 0.78 in DCM) from **10** (R_f = 0.42 in DCM). To evaluate separation, UV provided visualization of iodobenzene and the TLC plates were subsequently stained with $KMnO_4$ for visualization of the aldehyde. After combining the product fractions, they were concentrated to roughly 10% of their starting volume, and ^{1}H NMR of the solution provided a molar ratio of DCM to the product. From the molar ratio, a concentration was calculated, and this produced 2.354 grams of aldehyde **10** (25.45 mmol, 63% yield). Spectra matched former reports, and a ^{1}H NMR is provided below. ^{1}H NMR (400 MHz, CHLOROFORM-*D*) δ 9.78 (t, J = 1.1 Hz, 1H), 3.80 (t, J = 6.5 Hz, 2H), 2.94 (td, J = 6.5 Hz, 1.1 Hz, 2H).

HCl Addition Approach:

3-chloropropanal **10** was synthesized via the hydrolysis of acrolein diethyl acetal and HCl addition to the alkene. Below details the reaction on a 192 mmol scale (25 grams of starting material), which was the largest scale we performed. This produced a 70% yield of the product, but yields ranged slightly for this preparation, and the literature would suggest that better yields were possible.

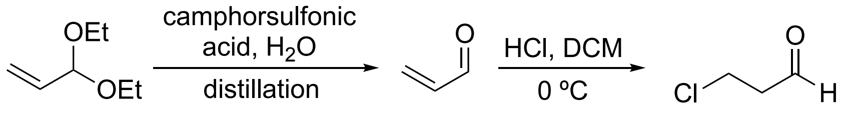

The reaction was based on a report in *Organic Process and Research Development*[3] and *Journal of Antibiotics*.[4] Acrolein diethyl acetal (25 grams, 192 mmol) and water (7.15 mL) were added to a round-bottomed flask equipped with a magnetic stir bar, and camphorsulfonic acid (1.494 grams, 6.43 mmol) was added to this mixture. It was stirred until it was a homogenous solution, which occurred along with a mild endotherm, and the mixture was stirred until it naturally came back to room temperature, which occurred over 45 minutes. The flask was fitted with a simple distillation apparatus, and a distillation occurred under atmospheric pressure at 65-74 °C. The distillate was extracted into DCM (100 mL), which was subsequently washed with saturated brine (initially 3 X 100 mL) until an absence of ethanol was found via ^{1}H NMR. This completed reaction 1, providing a solution of **15** in dichloromethane in a molar ratio of roughly 1:8 between the product **15** and DCM. At this point, the solution of **15** was added to a three-necked round-bottomed flask, and one opening was sealed with a glass stopper. The other two necks were fitted to allow gaseous HCl to flow into the flask and out into a solution of sodium bicarbonate (for quenching unreacted HCl). This flask was cooled in an ice bath. Sodium chloride was added to a secondary two-neck round-bottomed flask, and that flask was fitted with an addition funnel that was filled with concentrated sulfuric acid. The second neck was fitted with a hose that connected the two flasks. As the concentrated sulfuric acid mixed with the sodium chloride, gaseous HCl was produced, and it flowed through the tubing into the solution of **15**. During the reaction, pipette tips of the reaction mixture were taken and diluted into 500 μL of chloroform-D to analyze the reaction progress using ^{1}H NMR. The reaction was complete within 4 hours, producing 7.536 grams of aldehyde **10** (134.4 mmol, 70% yield). Spectra matched what was described on the last page. An image of the second reaction is on the following page, as is NMR data from analyzing the reaction aliquots of reaction 2. It shows at time zero that only acrolein is present, at an hour there is roughly a 1:1 ratio between acrolein and the 3-chloropropanal, and at 4 hours the reaction is complete.

Reaction 2: HCl addition to acrolein setup.

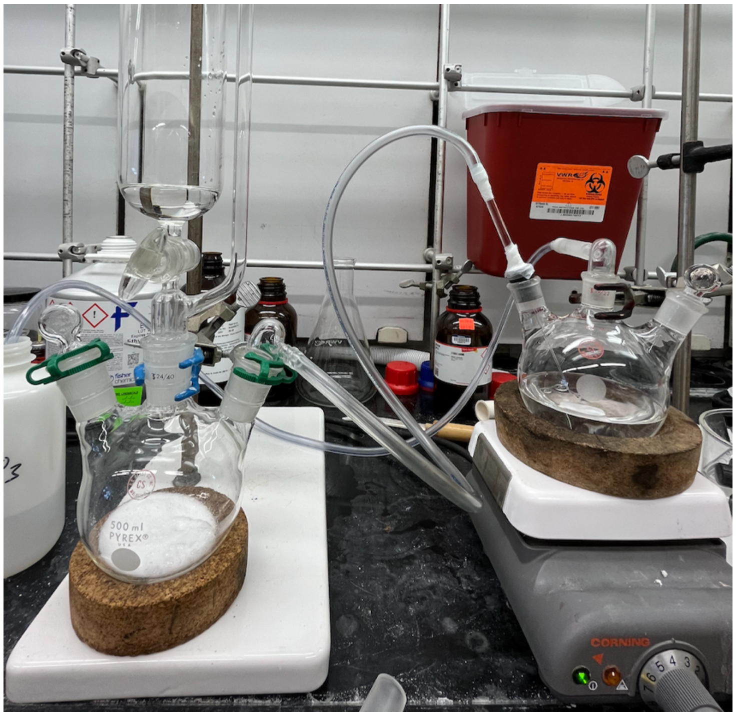

Reaction 2: NMR aliquot showing reaction progress.

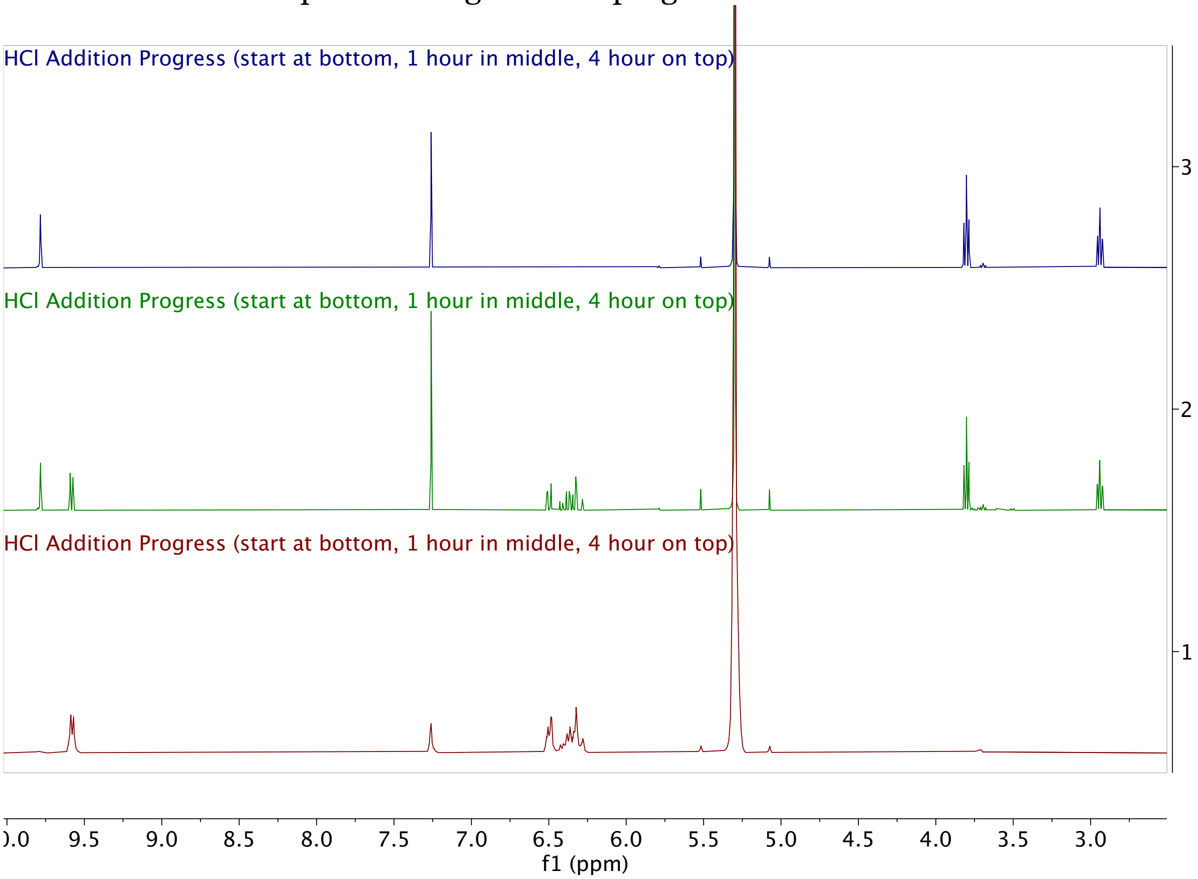

Chlorosulfinamide 17 Synthesis

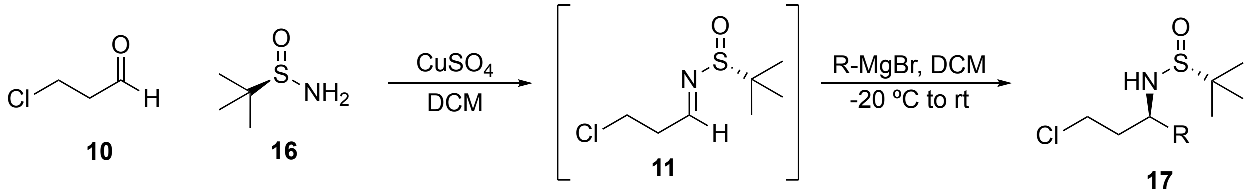

These reactions were generally performed on a 1 mmol scale unless otherwise specified. A solution of the 3-chloropropanal **10** (1.0 eq, DCM solution between 0.5-1 M) was added to a round-bottomed flask equipped with a magnetic stir bar, and the solution was diluted with dichloromethane to a final concentration of 0.25 M. To this solution was added sulfinamide **16 (**1.00 eq) followed by 1.20 eq of anhydrous copper(II) sulfate. The reaction was sealed and stirred for 24 hours, with the color changing from a murky white to a cloudy green/yellow solution.

The reaction mixture (cloudy green/yellow) was purged with argon and was placed in an NaCl/ice bath where a temperature of -20 °C was maintained. Grignard reagent (R-MgBr, 2.5 eq) was added through a syringe under argon to the reaction mixture dropwise to avoid increasing the reaction temperature. The reaction was maintained at -20°C for 1.5 hours then allowed to warm to room temperature.

The reaction mixture, now dark brown in color, was quenched with ~1 mL of saturated ammonium chloride and was diluted with 50 mL ethyl acetate. An additional 50 mL of saturated ammonium chloride was added, and the contents were placed in a separatory funnel where the organic and aqueous layers were separated. After collecting the organic, the organic material was extracted from the aqueous an additional two times with ethyl acetate (2 X 50 mL). The combined organic layer was dried with sodium sulfate, filtered, and was concentrated under reduced pressure.

The reaction mixture was weighed in a pre-weighed scintillation vial and a sample was analyzed using ^{1}H NMR and quantitative ^{13}C NMR (512 scans) for analysis of diastereomeric ratio, determined prior to purification. For purification, a gradient of 0% ethyl acetate in hexanes to 100% ethyl acetate in hexanes over 25 column volumes was used with a 12-gram silica cartridge. The crude product was loaded onto a loading cartridge packed with silica gel (~15 grams) using a minimum amount of hexanes or dichloromethane.

After chromatography, an iodine chamber was used to stain TLC plates of the fractions, which were developed in 1:1 ethyl acetate/hexanes. Relevant fractions were concentrated and yields for the separately combined fractions were recorded, and NMR spectra were acquired for each fraction set to observe the relative purity. Fractions with product signals were combined and concentrated to determine product yield. Citations for the primary literature involving the synthesis of the sulfinamide chiral auxiliary,[5] formation of the sulfinimines,[6] and a broad review[7] of their use are found below. Stereochemistry was assigned according to literature precedent involving Grignard additions to sulfinimines under the same conditions,[5,8] and we would direct the readers to table 1 and 2 of citation 8 for further details.

Commercial Grignard Reagents Used

- Phenylmagnesium bromide, 3.0 M in Et_2O, Sigma-Aldrich, 171565-100ML
- 4-fluorophenylmagnesium bromide, 2.0 M in Et_2O, Sigma-Aldrich, 245550-100ML
- 4-methoxyphenylmagnesium bromide, 0.5 M in THF, Sigma-Aldrich, 470260-100ML
- Methylmagnesium bromide, 3.0 M in Et_2O, Sigma-Aldrich, 189898-100ML
- Vinylmagnesium bromide, 1.0 M in THF, Sigma-Aldrich, 225584-100ML
- Allylmagnesium bromide, 1.0 M in Et_2O, Sigma-Aldrich, 225754-100ML
- Hexylmagnesium bromide, 2.0 M in Et_2O, Sigma-Aldrich, 255025-100ML
- Isopropylmagnesium bromide, 2.9 M in 2-Me-THF, Sigma-Aldrich, 703567-100ML
- Cyclohexylmagnesium bromide, 2.0 M in Et_2O, Sigma-Aldrich, 224413-100ML
- Cyclopentylmagnesium bromide, 2.0 M in Et_2O, Sigma-Aldrich, 428337-100ML
- *Tert*-butylmagnesium chloride, 2.0 M in Et_2O, Sigma-Aldrich, 224499-100ML

Chlorosulfinamide Analogues

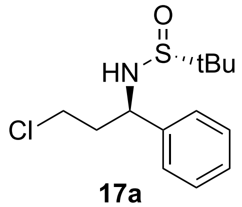

17a

17a was prepared according to the general chlorosulfinamide synthesis procedure using 1.0 equiv. of (*R*)-*tert*-butyl-sulfinamide, 1.0 equiv. of 3-chloropropanal, and 1.20 equiv. of copper sulfate in dichloromethane on a one mmol scale. After the reaction went to completion for 22 hours, 2.50 equiv. of phenylmagnesium bromide was added to the flask for the Grignard addition. The crude was purified by flash column chromatography and yielded the desired product (flakey white solid, 205 mg, 75% yield, DR: 85:15). ^{1}H NMR (400 MHz, CHLOROFORM-*D*) δ 7.39-7.29 (m, 5H), 4.66-4.55 (m, 1H), 3.57 – 3.46 (m, 1H), 3.42, (d, *J* = 4.5 Hz, 1H), 3.40 – 3.29 (m, 1H), 2.52-2.41 (m, 1H), 2.24 – 2.13 (m, 1H), 1.23 (s, 9H). ^{13}C{1H} NMR (126 MHz, CHLOROFORM-*D*) δ 141.2, 128.9, 128.2, 127.0, 56.8, 56.0, 41.1, 39.4, 22.5. HRMS (ESI$^+$) $C_{13}H_{21}ClNOS^+$ $[M+H]^+$: calc. mass 274.1027, found 274.1029. $[\alpha]_D^{20}$ = +57.0° (*c* 0.0078, CH_2Cl_2).

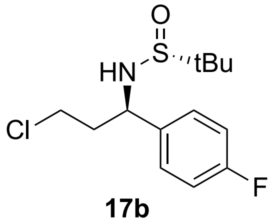

17b

17b was prepared according to the general chlorosulfinamide synthesis procedure using 1.0 equiv. of (*R*)-*tert*-butyl-sulfinamide, 1.0 equiv. of 3-chloropropanal, and 1.20 equiv. of copper sulfate in dichloromethane on a one mmol scale. After the reaction went to completion over 24 hours, 2.50 equiv. of 4-fluoro-phenyl magnesium bromide was added to the flask for the Grignard addition. The crude was purified by flash column chromatography and yielded the desired product (brown oil, 178 mg, 61% yield, DR: 87:13). ^{1}H NMR (400 MHz, CHLOROFORM-*D*) δ 7.36 – 7.30 (m, 2H), 7.09 – 7.02 (m, 2H), 4.60 (dd, *J* = 12.2, 6.9 Hz, 1H), 3.56 – 3.48 (m, 1H), 3.40 – 3.32 (m, 2H), 2.45 (ddt, *J* = 13.8, 7.2, 6.5 Hz, 1H), 2.15 (td, *J* = 14.1, 6.3 Hz, 1H), 1.23 (s, 9H). ^{13}C{1H} NMR (126 MHz, CHLOROFORM-*D*) δ 162.5 (*J* = 247.1 Hz), 137.1 (*J* = 3.3 Hz), 128.8 (*J* = 8.2 Hz), 115.9 (*J* = 21.4 Hz), 56.3, 56.1, 41.1, 39.4, 22.6. HRMS (ESI$^+$) $C_{13}H_{20}ClFNOS^+$ [M+H]$^+$: calc. mass 292.0933, found 292.0935. $[\alpha]_D^{20}$ = -10.0° (*c* 0.005, CH_2Cl_2).

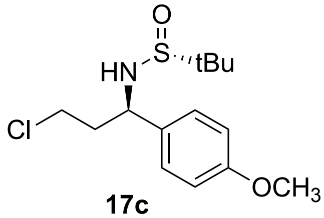

17c

17c was prepared according to the general chlorosulfinamide synthesis procedure using 1.0 equiv. of (*R*)-*tert*-butyl-sulfinamide, 1.0 equiv. of 3-chloropropanal, and 1.20 equiv. of copper sulfate in dichloromethane on a one mmol scale. After the reaction went to completion over 24 hours, 2.50 equiv. of 4-methoxy-phenyl magnesium bromide was added to the flask for the Grignard addition. The crude was purified by flash column chromatography and yielded the desired product (brown oil, 157 mg, 52% yield, DR: 79:21). ^{1}H NMR (400 MHz, CHLOROFORM-*D*) δ 7.22 (d, *J* = 8.3 Hz, 2H), 6.84 (d, *J* = 8.3 Hz, 2H), 4.57 – 4.45 (m, 1H), 3.75 (s, 3H), 3.50 – 3.43 (m, 1H), 3.43 – 3.37 (m, 1H), 3.35 – 3.23 (m, 1H), 2.40 (dq, *J* = 13.7, 6.8 Hz, 1H), 2.09 (dt, *J* = 13.6, 6.8, 1H), 1.17 (s, 9H). ^{13}C{1H} NMR (101 MHz, CHLOROFORM-*D*) δ 159.4, 133.1, 128.3, 114.3, 56.3, 55.9, 55.3, 41.3, 39.4, 22.6. HRMS (ESI$^+$) $C_{14}H_{23}ClNO_2S^+$ [M+H]$^+$: calc. mass 304.1133, found 304.1136. $[\alpha]_D^{20}$ = -33° (*c* 0.005, CH_2Cl_2).

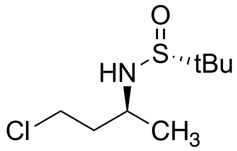

17d

17d was prepared according to the general chlorosulfinamide synthesis procedure using 1.0 equiv. of (*R*)-*tert*-butyl-sulfinamide, 1.00 equiv. of 3-chloropropanal, and 1.20 equiv. of copper sulfate in dichloromethane on a one mmol scale. After the reaction went to completion after 24 hours, 2.50 equiv. of methyl magnesium bromide was added to the flask for the Grignard addition. The crude was purified by flash column chromatography and yielded the desired product (yellow oil, 137 mg, 64% yield, DR: >95:5). ^{1}H NMR (400 MHz, CHLOROFORM-*D*) δ 3.65 – 3.51 (m, 3H), 2.92 (d, *J* = 8.1 Hz, 1H), 1.93 – 1.82 (m, 1H), 1.30 (d, *J* = 6.6f Hz, 3H), 1.18 (s, 9H). ^{13}C{1H} NMR (101 MHz, CHLOROFORM-*D*) δ 55.9, 50.5, 41.6, 40.7, 23.3, 22.6. HRMS (ESI^{+}) $C_8H_{19}ClNOS^+$ [M+H]$^+$: calc. mass 212.0870, found 212.0870. $[\alpha]_D^{20}$ = -6° (*c* 0.049, CH_2Cl_2).

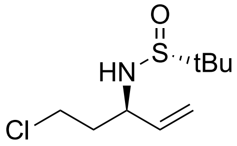

17e

17e was prepared according to the general chlorosulfinamide synthesis procedure using 1.0 equiv. of (*R*)-*tert*-butyl-sulfinamide, 1.00 equiv. of 3-chloropropanal, and 1.20 equiv. of copper sulfate in dichloromethane on a one mmol scale. After the reaction went to completion after 24 hours, 2.50 equiv. of vinyl magnesium bromide was added to the flask for the Grignard addition. The crude was purified by flash column chromatography and yielded the desired product (orange oil, 100 mg, 45% yield, DR: 70:30). Diastereomers were partially separated during flash purification, resulting in fractions with varying DR values, described from nonpolar to polar collected fractions. These include 16 mg (1:99 DR), 12 mg (24:76 DR), 24 mg (80:20 DR) and the most polar fractions containing 48 mg (>99:1 DR). ^{1}H NMR (500 MHz, CHLOROFORM-*D*) δ 5.73 – 5.63 (m, 1H), 5.33 – 5.28 (m, 1H), 5.25-5.21 (m, 1H), 4.07 – 4.01 (m,1H), 3.70 – 3.64 (m, 1H), 3.59 – 3.52 (m, 1H) 3.28, (d, *J* = 4.6 Hz, 1H), 2.15 – 1.98 (m, 2H), 1.21 (s, 9H). ^{13}C{1H} NMR (101 MHz, CHLOROFORM-*D*) δ 137.9, 118.1, 56.4, 55.8, 41.2, 38.7, 22.7. HRMS (ESI^{+}) $C_9H_{19}ClNOS^+$ [M+H]$^+$: calc. mass 224.0870, found 224.0871. $[\alpha]_D^{20}$ = -43° (*c* 0.045, CH_2Cl_2).

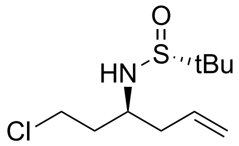

17f

17f was prepared according to the general chlorosulfinamide synthesis procedure using 1.0 equiv. of (*R*)-*tert*-butyl-sulfinamide, 1.00 equiv. of 3-chloropropanal, and 1.20 equiv. of copper sulfate in dichloromethane on a one mmol scale. After the reaction went to completion after 24 hours, 2.50 equiv. of allyl magnesium bromide was added to the flask for the Grignard addition. The crude was purified by flash column chromatography and yielded the desired product (flaky white solid, 170 mg, 71% yield, DR: 95:5). ^{1}H NMR (400 MHz, CHLOROFORM-*D*) δ 5.81 – 5.68 (m, 1H), 5.16 (s, 1H), 5.14-5.11 (m, 1H), 3.63 – 3.57 (m, 2H), 3.57 – 3.49 (m, 1H), 3.23 (d, *J* = 7.6 Hz, 1H), 2.45-2.38 (m, 2H), 2.00-1.80 (m, 2H), 1.18 (s, 9H). ^{13}C{1H} NMR (126 MHz, CHLOROFORM-*D*) δ 133.3, 119.3, 55.9, 53.0, 41.4, 40.5, 37.7, 22.5. HRMS (ESI$^+$) $C_{10}H_{21}ClNOS^+$ [M+H]$^+$: calc. mass 238.1027, found 238.1027. $[\alpha]_D^{20}$ = -16° (*c* 0.006, CH_2Cl_2).

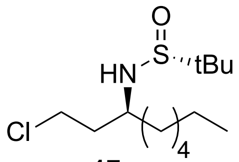

17g

17g was prepared according to the general chlorosulfinamide synthesis procedure using 1.0 equiv. of (*R*)-*tert*-butyl-sulfinamide, 1.0 equiv. of 3-chloropropanal, and 1.20 equiv. of copper sulfate in dichloromethane on a one mmol scale. After the reaction went to completion over 24 hours, 2.50 equiv. of hexyl magnesium bromide was added to the flask for the Grignard addition. The crude was purified by flash column chromatography and yielded the desired product (brown oil, 216 mg, 77% yield, DR: 92:8). ^{1}H NMR (400 MHz, CHLOROFORM-*D*) δ 3.70 – 3.57 (m, 2H), 3.48 – 3.38 (m, 1H), 3.01 (d, *J* = 7.5 Hz, 1H), 2.04 – 1.95 (m, 1H), 1.91 – 1.80 (m, 1H), 1.75 – 1.64 (m, 1H), 1.60 – 1.49 (m, 1H), 1.42 – 1.25 (m, 8H), 1.22 (s, 9H), 0.87 (t, *J* = 6.8 Hz, 3 H). ^{13}C{1H} NMR (126 MHz, CHLOROFORM-*D*) δ 55.8, 54.4, 41.5, 38.3, 36.4, 31.5, 28.9, 25.5, 22.5, 22.4, 13.9. HRMS (ESI$^+$) $C_{13}H_{29}ClNOS^+$ [M+H]$^+$: calc. mass 282.1653, found 282.1650. $[\alpha]_D^{20}$ = -4° (*c* 0.007, CH_2Cl_2).

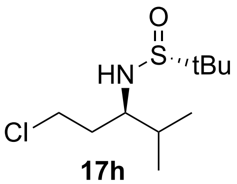

17h was prepared according to the general chlorosulfinamide synthesis procedure using 1.0 equiv. of (*R*)-*tert*-butyl-sulfinamide, 1.00 equiv. of 3-chloropropanal, and 1.20 equiv. of copper sulfate in dichloromethane on a one mmol scale. After the reaction went to completion after 24 hours, 2.50 equiv. of isopropyl magnesium bromide was added to the flask for the Grignard addition. The crude was purified by flash column chromatography and yielded the desired product (flaky white solid, 105 mg, 44% yield, DR: >95:5). ^{1}H NMR (400 MHz, CHLOROFORM-*D*) δ 3.64 – 3.54 (m, 2H), 3.30 – 3.23 (s, 1H), 3.13 (d, *J* = 8.5 Hz, 1H), 2.04 – 1.96 (m, 1H), 1.93 – 1.84 (m, 1H), 1.80 – 1.71 (m, 1H), 1.18 (s, 9H), 0.91 (dd, *J* = 6.9, 3.6 Hz, 6H). ^{13}C{1H} NMR (126 MHz, CHLOROFORM-*D*) δ 59.7, 56.3, 42.1, 34.8, 32.7, 22.8, 18.8, 17.5. HRMS (ESI^{+}) $C_{10}H_{23}ClNOS^{+}$ [M+H]$^{+}$: calc. mass 240.1183, found 240.1179. $[\alpha]_D^{20}$ = +5° (*c* 0.006, CH_2Cl_2).

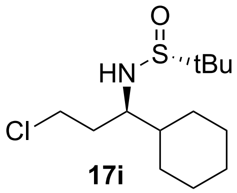

17i was prepared according to the general chlorosulfinamide synthesis procedure using 1.0 equiv. of (*R*)-*tert*-butyl-sulfinamide, 1.0 equiv. of 3-chloropropanal, and 1.20 equiv. of copper sulfate in dichloromethane on a one mmol scale. After the reaction went to completion over 24 hours, 2.50 equiv. of cyclohexyl magnesium bromide was added to the flask for the Grignard addition. The crude was purified by flash column chromatography and yielded the desired product (white solid, 184 mg, 66% yield, DR: >95:5) ^{1}H NMR (500 MHz, CHLOROFORM-*D*) δ 3.65 – 3.52 (m, 2H), 3.29 – 3.22 (m, 1H), 3.17 (d, *J* = 8.5 Hz, 1H), 1.97 – 1.87 (m, 1H), 1.83 – 1.77 (m, 1H), 1.76 – 1.58 (m, 6H), 1.28 – 1.21 (m, 2H), 1.19 (s, 9H), 1.15 – 1.05 (m, 2H), 1.05 – 0.94 (m, 1H). ^{13}C{1H} NMR (101 MHz, CHLOROFORM-*D*) δ 59.3, 56.2, 42.9, 42.1, 35.5, 29.4, 28.1, 26.4, 26.2, 26.2, 22.7. HRMS (ESI^{+}) $C_{13}H_{27}ClNOS^{+}$ [M+H]$^{+}$: calc. mass 280.1496, found 280.1493. $[\alpha]_D^{20}$ = +22° (*c* 0.011, CH_2Cl_2).

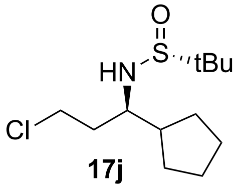

17j

17j was prepared according to the general chlorosulfinamide synthesis procedure using 1.0 equiv. of (*R*)-*tert*-butyl-sulfinamide, 1.0 equiv. of 3-chloropropanal, and 1.20 equiv. of copper sulfate in dichloromethane on a one mmol scale. After the reaction went to completion over 24 hours, 2.50 equiv. of cyclopentyl magnesium bromide was added to the flask for the Grignard addition. The crude was purified by flash column chromatography and yielded the desired product (flakey white solid, 149 mg, 58% yield, DR: >95:5). ^{1}H NMR (500 MHz, CHLOROFORM-*D*) δ 3.67 – 3.57 (m, 2H), 3.31 – 3.23 (m, 1H), 3.18 (d, *J* = 6.3 Hz, 1H), 2.11 – 1.95 (m, 2H), 1.92 – 1.84 (m, 1H), 1.83 – 1.69 (m, 2H), 1.66 – 1.56 (m, 2H), 1.56 – 1.46 (m, 2H), 1.33 – 1.21 (m, 2H), 1.18 (s, 9H). ^{13}C{1H} NMR (101 MHz, CHLOROFORM-*D*) δ 58.3, 56.0, 44.9, 41.7, 37.5, 29.9, 29.5, 25.6, 25.3, 22.7. HRMS (ESI$^+$) $C_{12}H_{25}ClNOS^+$ [M+H]$^+$: calc. mass 266.1340, found 266.1337. $[\alpha]_D^{20}$ = -22° (*c* 0.014, CH_2Cl_2).

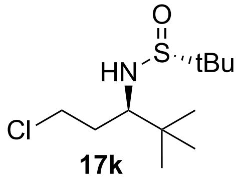

17k

17k was prepared according to the general chlorosulfinamide synthesis procedure using 1.0 equiv. of (*R*)-*tert*-butyl-sulfinamide, 1.20 equiv. of 3-chloropropanal, and 3.00 equiv. of copper sulfate in dichloromethane on a 2 mmol scale. After the reaction went to completion over 24 hours, 2.50 equiv. of *tert*-butyl magnesium chloride was added to the flask without removing the copper sulfate at -46 °C in a dry ice/acetonitrile bath. The bath was allowed to warm to room temperature over several hours and the reaction was left to stir overnight. The crude was purified by flash column chromatography (gradient of 0% ethyl acetate in diethyl ether → 50% ethyl acetate in diethyl ether), yielding the desired product (brown oil, 137 mg, 27% yield, DR: 56:44). ^{1}H NMR (400 MHz, CHLOROFORM-*D*) δ 3.94 – 3.85 (m, 1H), 3.09 – 3.02 (m, 1H), 2.89 (d, *J* = 8.3 Hz, 1H), 2.25 – 2.14 (m, 1H), 1.70 – 1.60 (m, 1H), 1.25 (s, 9H), 0.92 (s, 9H). ^{13}C{1H} NMR (101 MHz, CHLOROFORM-*D*) δ 62.8, 62.7, 56.63, 56.57, 42.7, 42.3, 35.5, 35.1, 34.7, 34.5, 27.0, 26.8, 23.1, 22.9. Note, carbon signals are listed for both diastereomers. HRMS (ESI$^+$) $C_{11}H_{25}ClNOS^+$ [M+H]$^+$: calc. mass 254.1340, found 254.1343. $[\alpha]_D^{20}$ = -33° (*c* 0.006, CH_2Cl_2).

Protected Azetidine 18 Synthesis

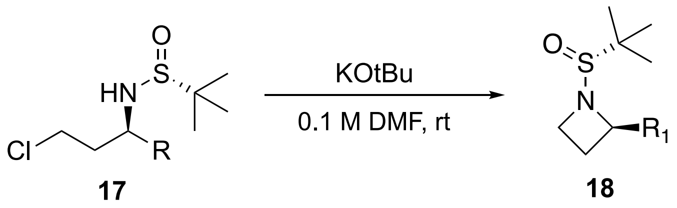

To a scintillation vial containing a magnetic stir bar was added chlorosulfinamides **17** (1.0 eq.), and they were dissolved in Dimethyl formamide (DMF, 0.1 M) and cooled to 0 °C in an ice bath before addition of potassium *tert*-butoxide (KO^{t}Bu, 1.5 eq.). After addition, the reaction was immediately allowed to warm to room temperature, and it was left to stir for an hour. After checking for completion by TLC, it was worked up. In the rare case that starting material remained (compounds **18c** and **18g**), an additional 1.5 equivalents of KO^{t}Bu was added, which led to reaction completion within 30 minutes.

The reaction was partitioned between diethyl ether (30 mL) and saturated sodium bicarbonate (50 mL) into a separatory funnel, and it was extracted with diethyl ether (3 X 30 mL). The organic layer was dried with sodium sulfate and was concentrated using a rotary evaporator, ultimately pulling a maximum vacuum of 80 torr for 5 minutes until dripping slowed. For purification, a 12-gram silica cartridge was used, and the crude was added to a loading cartridge packed with silica gel (~15 grams) using a minimum amount of diethyl ether. For most analogues, the column was run isocratic with 100% diethyl ether, but in some cases (noted for specific molecules below), a gradient of ethyl acetate and hexanes was used. Fractions were analyzed via 254 nm UV light, iodine staining, or potassium permanganate staining.

Azetidine Analogues

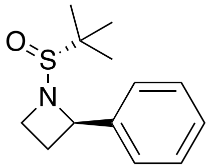

18a

18a was prepared according to the general azetidine synthesis procedure. Reaction occurred on a 0.486 mmol scale, and the starting material had a *dr* of 85:15. The crude was purified by flash column chromatography (100% diethyl ether, 25 CVs), yielding the desired product (white solid/oil, 103 mg, 89% yield, DR 85:15). ^{1}H NMR (500 MHz, CHLOROFORM-*D*) δ 7.48-7.44 (m, 2H), 7.33 (ddd, J = 7.3, 6.6, 1.1 Hz, 2H), 7.28-7.22 (m, 1H), 5.37 (dd, J = 9.3, 7.4 Hz, 1H), 4.02-3.94 (m, 1H), 3.88-3.81 (m, 1H), 2.75-2.64 (m, 1H), 2.34-2.23 (m, 1H), 0.90 (s, 9H). ^{13}C{1H} NMR (101 MHz, CHLOROFORM-*D*) δ 143.3, 128.7, 128.0, 127.7, 58.5, 56.5, 49.0, 29.9, 23.5. HRMS (ESI^{+}) $C_{13}H_{20}NOS^{+}$ [M+H]$^{+}$: calc. mass 238.1260, found 238.1260. $[\alpha]_D^{20}$ = -137° (*c* 0.024, CH_2Cl_2).

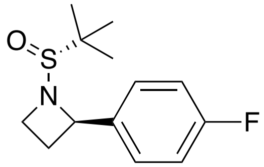

18b

18b was prepared according to the general azetidine synthesis procedure. Reaction occurred on a 0.329 mmol scale, and the starting material had a *dr* of ~95:5. The crude was purified by flash column chromatography (gradient 30% → 50% ethyl acetate in hexanes), yielding the desired product (yellow oil, 44 mg, 52% yield, DR ~95:5). ^{1}H NMR (400 MHz, CHLOROFORM-*D*) δ 7.45 (dd, J = 8.7, 5.5 Hz, 2H), 7.03 (t, J = 8.7 Hz, 2H), 5.36 (dd, J = 9.2, 7.5 Hz, 1H), 4.00-3.92 (m, 1H), 3.88-3.80 (m, 1H), 2.75-2.65 (m, 1H), 2.33-2.22 (m, 1H), 0.91 (s, 9H). ^{19}F NMR (376 MHz, CHLOROFORM-*D*) δ -114.09 (td, J = 8.6, 4.3 Hz), -114.41 (td J = 8.7, 4.2 Hz). ^{13}C{1H} NMR (101 MHz, CHLOROFORM-*D*) δ 161.4 (J = 246.3 Hz), 139.2 (J = 3.2 Hz), 129.4 (J = 8.2 Hz), 115.6 (J = 21.3 Hz), 57.9, 56.5, 48.8, 29.2, 23.4. HRMS (ESI^{+}) $C_{13}H_{19}FNOS^{+}$ [M+H]$^{+}$: calc. mass 256.1166, found 256.1169. $[\alpha]_D^{20}$ = -72° (*c* 0.002, CH_2Cl_2).

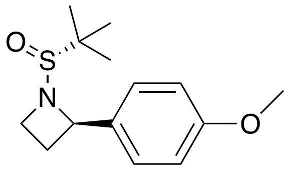

18c

18c was prepared according to the general azetidine synthesis procedure with one modification. The reaction did not appear to be complete after several hours according to TLC. At this point, an additional 1.5 equiv. of KO^{t}Bu was added, and the reaction was complete within an additional 30 minutes. Reaction occurred on a 0.369 mmol scale, and the starting material had a *dr* of 80:20. The crude was purified by flash column chromatography (25-55% ethyl acetate in hexanes), yielding the desired product (yellow oil, 54 mg, 55% yield, DR 80:20). ^{1}H NMR (400 MHz, CHLOROFORM-*D*) δ 7.38 (d, *J* = 8.7 Hz, 2H), 6.86 (d, *J* = 8.7 Hz, 2H), 5.31 (dd, *J* = 9.2, 7.6 Hz, 1H), 3.96-3.88 (m, 1H), 3.85-3.79 (m, 1H), 3.78 (s, 3H), 2.70-2.60 (m, 1H), 2.32-2.22 (m, 1H), 0.90 (s, 9H). ^{13}C{1H} NMR (126 MHz, CHLOROFORM-*D*) δ 159.3, 135.4, 129.0, 113.9, 58.4, 56.4, 55.3, 48.5, 29.2, 23.5. HRMS (ESI$^+$) $C_{14}H_{22}NO_2S$ [M+H]$^+$: calc. mass 268.1371, found 268.1369. $[\alpha]_D^{20}$ = -39° (*c* 0.016, CH_2Cl_2).

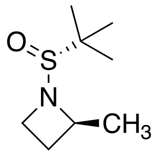

18d

18d was prepared according to the general azetidine synthesis procedure. Reaction occurred on a 0.236 mmol scale, and the starting material had a *dr* of >95:5. The crude was purified by flash column chromatography (100% diethyl ether, 25 CVs), yielding the desired product (yellow oil, 21 mg, 51% yield, *dr* > 95:5). ^{1}H NMR (400 MHz, CHLOROFORM-*D*) δ 4.39 – 4.29 (m, 1H), 4.14 – 4.06 (m, 1H), 3.58 – 3.51 (m, 1H), 2.57 – 2.47 (m, 1H), 1.91 – 1.81 (m, 1H), 1.45 (d, *J* = 6.4 Hz, 3H), 1.13 (s, 9H). ^{13}C{1H} NMR (101 MHz, CHLOROFORM-*D*) δ 58.7, 56.6, 45.8, 26.7, 23.6, 21.6. HRMS (ESI$^+$) $C_8H_{18}NOS^+$ [M+H]$^+$: calc. mass 176.1104, found 176.1103. $[\alpha]_D^{20}$ = -107° (*c* 0.024, CH_2Cl_2).

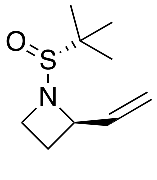

18e

18e was prepared according to the general azetidine synthesis procedure. Reaction occurred on a 0.201 mmol scale, and the starting material had a *dr* of >95:5. The crude was purified by flash column chromatography (100% diethyl ether, 25 CVs), yielding the desired product (yellow oil, 14 mg, 37% yield, *dr* >95:5). ^{1}H NMR (400 MHz, CHLOROFORM-*D*) δ 6.24 – 6.13 (m, 1H), 5.23 – 5.15 (m, 2H), 4.77-4.69 (m, 1H), 4.03 – 3.94 (m, 1H), 3.70 – 3.61 (m, 1H), 2.64 – 2.53 (m, 1H), 2.15 – 2.05 (m, 1H), 1.13 (s, 9H). ^{13}C{1H} NMR (101 MHz, CHLOROFORM-D) δ 139.5, 118.0, 62.6, 56.8, 46.8, 26.0, 23.7. HRMS (ESI$^+$) $C_9H_{18}NOS^+$ [M+H]$^+$: calc. mass 188.1104, found 188.1104. $[\alpha]_D^{20}$ = -116 (*c* 0.014, CH_2Cl_2).

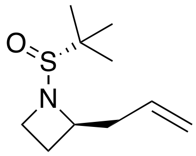

18f

18f was prepared according to the general azetidine synthesis procedure. Reaction occurred on a 0.662 mmol scale, and the starting material had a *dr* of 95:5. The crude was purified by flash column chromatography (100% diethyl ether, 25 CVs), yielding the desired product (yellow oil, 89 mg, 67% yield, *dr* = 95:5). ^{1}H NMR (400 MHz, CHLOROFORM-*D*) δ 5.73 – 5.59 (m, 1H), 5.06 – 4.97 (m, 2H), 4.27 – 4.17 (m, 1H), 4.02 – 3.93 (m, 1H), 3.55 – 3.45 (m, 1H), 2.64 – 2.54 (m, 1H), 2.51 – 2.42 (m, 1H), 2.42 – 2.31 (m, 1H), 1.98 – 1.86 (m, 1H), 1.07 (m, 9H). ^{13}C{1H} NMR (101 MHz, CHLOROFORM-*D*) δ 133.1, 117.8, 60.9, 56.5, 46.2, 39.3, 24.0, 23.3. HRMS (ESI$^+$) $C_{10}H_{20}NOS$ [M+H]$^+$: calc. mass 202.1266, found 202.1260. $[\alpha]_D^{20}$ = -69 (*c* 0.025, CH_2Cl_2).

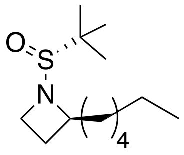

18g

18g was prepared according to the general azetidine synthesis procedure. Reaction occurred on a 0.145 mmol scale, and the starting material had a *dr* of > 95:5, and 3.00 equiv. of potassium *tert*-butoxide was used. The crude was purified by flash column chromatography (gradient 30% → 60% ethyl acetate in hexanes), yielding the desired product (yellow oil, 28 mg, 78% yield, *dr* > 95:5). ^{1}H NMR (400 MHz, CHLOROFORM-*D*) δ 4.24 – 4.14 (m, 1H), 4.14 – 4.01 (m, 1H), 3.60-3.51 (m, 1H), 2.51 – 2.39 (m, 1H), 1.98 – 1.86 (m, 2H), 1.82 – 1.70 (m, 1H), 1.31 – 1.21 (m, 8H), 1.13 (s, 9H), 0.86 (t, *J* = 6.7 Hz, 3H). ^{13}C{1H} NMR (101 MHz, CHLOROFORM-*D*) δ 62.7, 56.5, 46.0, 35.1, 31.9, 29.3, 25.0, 24.8, 23.5, 22.7, 14.2. HRMS (ESI^{+}) $C_{13}H_{28}NOS^{+}$ $[M+H]^{+}$: calc. mass 246.1886, found 246.1892. $[\alpha]_D^{20}$ = +72 (*c* 0.002, CH_2Cl_2)

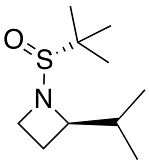

18h

18h was prepared according to the general azetidine synthesis procedure. Reaction occurred on a 0.254 mmol scale, and the starting material had a *dr* of > 95:5. The crude was purified by flash column chromatography (100% diethyl ether, 25 CVs), yielding the desired product (yellow oil, 26 mg, 50% yield, *dr* > 95:5). ^{1}H NMR (400 MHz, CHLOROFORM-*D*) δ 4.30 – 4.22 (m, 1H), 3.80 – 3.64 (m, 2H), 2.27 – 2.15 (m, 1H), 2.12 – 2.02 (m, 1H), 2.02 – 1.91 (m, 1H), 1.12 (s, 9H), 1.03 (d, *J* = 6.7 Hz, 3H), 0.82 (d, *J* = 6.7 Hz, 3H). ^{13}C{1H} NMR (101 MHz, CHLOROFORM-*D*) δ 64.4, 56.3, 48.5, 30.8, 23.2, 19.4, 18.7, 15.6. HRMS (ESI^{+}) $C_{10}H_{22}NOS^{+}$ $[M+H]^{+}$: calc. mass 204.1417, found 204.1414. $[\alpha]_D^{20}$ = -61 (*c* 0.024, CH_2Cl_2).

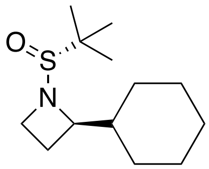

18i

18i was prepared according to the general azetidine synthesis procedure. Reaction occurred on a 0.622 mmol scale, and the starting material had a *dr* of > 95:5. The crude was purified by flash column chromatography (gradient 25% → 55% ethyl acetate in hexanes), yielding the desired product (clear/yellow oil, 88 mg, 58% yield, *dr* > 95:5). ^{1}H NMR (400 MHz, CHLOROFORM-*D*) δ 4.20 – 4.13 (m, 1H), 3.78 – 3.69 (m, 1H), 3.65 – 3.56 (m, 1H), 2.23 – 2.12 (m, 1H), 2.11 – 2.00 (m, 1H), 1.92 (d, *J* = 12.4 Hz, 1H), 1.77 – 1.69 (m, 1H), 1.68 – 1.53 (m, 4H), 1.22 – 1.12 (m, 2H), 1.10-0.98 (m, 11H), 0.80 (dd, *J* = 12.6, 3.3 Hz, 1H). $^{13}C\{1H\}$ NMR (101 MHz, CHLOROFORM-*D*) δ 64.7, 56.3, 48.3, 41.1, 29.3, 26.6, 26.03, 26.01, 25.8, 23.1, 20.7. HRMS (ESI^{+}) $C_{13}H_{26}NOS^{+}$ $[M+H]^{+}$: calc. mass 244.1730, found 244.1735. $[\alpha]_D^{20}$ = +60° (*c* 0.012, CH_2Cl_2).

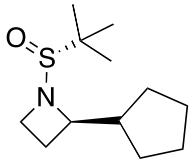

18j

18j was prepared according to the general azetidine synthesis procedure. Reaction occurred on a 0.177 mmol scale, and the starting material had a *dr* of ~85:15. The crude was purified by flash column chromatography (gradient 30% → 60% ethyl acetate in hexanes), yielding the desired product (yellow oil, 28 mg, 68% yield, *dr* = 85:15). ^{1}H NMR (400 MHz, CHLOROFORM-*D*) δ 4.24 – 4.12 (m, 1H), 4.09 – 3.95 (m, 1H), 3.57 – 3.47 (m, 1H), 2.50 – 2.30 (m, 2H), 2.02 – 1.95 (m, 1H), 1.94 – 1.84 (m, 1H), 1.81 – 1.71 (m, 1H), 1.65 – 1.46 (m, 5H), 1.35 – 1.14 (m, 1H), 1.12 (s, 9H). $^{13}C\{1H\}$ NMR (101 MHz, CHLOROFORM-*D*) δ 67.8, 56.8, 45.2, 43.5, 30.7, 27.9, 26.3, 25.6, 23.4, 22.9. HRMS (ESI^{+}) $C_{12}H_{24}NOS^{+}$ $[M+H]^{+}$: calc. mass 230.1573, found 230.1573. $[\alpha]_D^{20}$ = +46 (*c* 0.024, CH_2Cl_2).

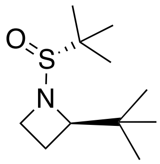

18k

18k was prepared according to the general azetidine synthesis procedure. Reaction occurred on a 0.276 mmol scale, the starting material had a *dr* of 56:44, and 3.00 equiv. of potassium *tert*-butoxide was used. The crude was purified by flash column chromatography (gradient 30% → 60% ethyl acetate in hexanes), yielding the desired product (yellow oil, 22 mg, 33% yield, DR 53:47). ^{1}H NMR (400 MHz, CHLOROFORM-*D*) δ 3.96 (dd, *J* = 9.1, 6.1 Hz, 1H), 3.80-3.71 (m, 1H), 3.58-3.49 (m, 1H), 2.35 (dtd, *J* = 11.3, 9.4, 6.4 Hz, 1H), 2.09-2.03 (m, 1H), 1.14 (s, 9H), 0.95 (s, 9H). ^{13}C{1H} NMR (101 MHz, CHLOROFORM-*D*) δ 73.6, 57.7, 47.3, 34.4, 25.6, 23.0, 20.9. HRMS (ESI$^+$) $C_{11}H_{24}NOS^+$ [M+H]$^+$: calc. mass 218.1573, found 218.1572. $[\alpha]_D^{20}$ = -17 (*c* 0.020, CH_2Cl_2).

Azetidine Derivatization (19-22)

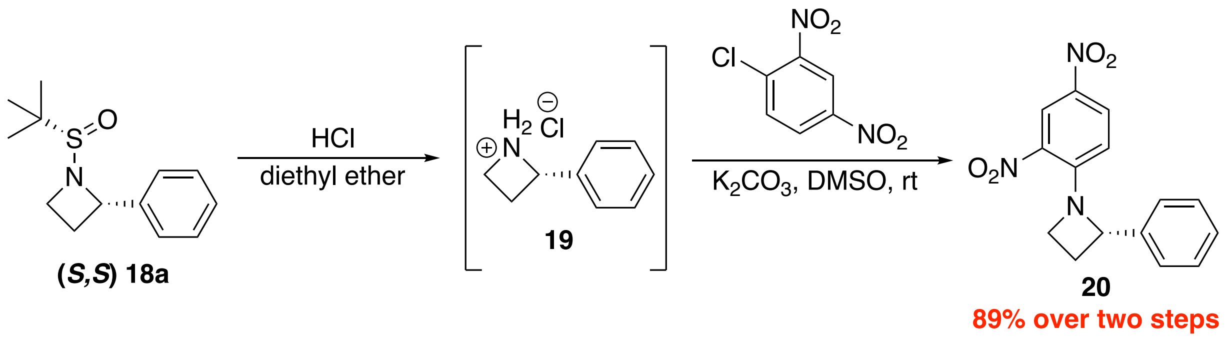

A round-bottomed flask equipped with a magnetic stir bar was charged with azetidine **18a** (100 mg, 0.421 mmol, 1.0 eq) and diethyl ether (8.43 mL, 0.05 M). The solution was stirred and was cooled in an ice bath. 4 M HCl in dioxane was added to the solution via syringe (0.42 mL, 1.69 mmol, 4.0 eq.), and the reaction immediately became cloudy. Within a minute, the ice bath was removed, and the reaction was allowed to stir at room temperature for an additional 30 minutes.

After 30 minutes, the reaction was diluted with 10 mL of diethyl ether, and it was passed through a syringe with a cotton plug. The round-bottomed flask was washed an additional two times with diethyl ether, and the solutions were also passed through the syringe filter (2 X 10 mL). The filtrate was colorless and was discarded. The filtercake was dissolved with dichloromethane, and the solution was passed through the filter and was collected separately. It was concentrated under reduced pressure, and this provided azetidine hydrochloride **19** as an off-white solid. This was taken on crude into the next reaction, but the proton and carbon NMR data for this crude product is also included below and is tabulated here. ^{1}H NMR (400 MHz, CHLOROFORM-*D*) δ 9.83 (s, br, 1H), 9.65 (s, br, 1H), 7.44-7.40 (m, 2H), 7.32-7.22 (m, 3H), 5.43-5.33 (m, 1H), 3.98-3.86 (m, 1H), 3.80-3.70 (m, 1H), 2.80-2.71 (m, 1H), 2.60-2.51 (m, 1H). ^{13}C{1H} NMR (101 MHz, CHLOROFORM-*D*) δ 134.7, 129.6, 128.9, 127.4, 62.3, 41.9, 26.4.

To a round bottomed flask equipped with a magnetic stir bar was added the crude azetidine hydrochloride salt **19**, which was dissolved in DMSO (0.75 mL, 0.56 M). Potassium carbonate (116 mg, 0.843 mmol, 2.0 eq) followed by 2,4-dinitrochlorobenzene (187 mg, 0.927 mmol, 2.2 eq) were added to the mixture at room temperature. The reaction was stirred overnight at room temperature.

The reaction mixture was partitioned between ethyl acetate (30 mL) and saturated bicarbonate (70 mL). The organic was extracted from the mixture with ethyl acetate (3 X 30 mL), and the

combined organic was washed with saturated brine (50 mL), dried with sodium sulfate, filtered, and concentrated under reduced pressure. The crude was purified by flash column chromatography (gradient 10% → 25% ethyl acetate in hexanes), yielding the desired product (yellow solid, 112 mg, 89% yield). ^{1}H NMR (400 MHz, CHLOROFORM-*D*) δ 8.62 (d, J = 2.6 Hz, 1H), 8.04 (dd, J1 = 9.4 Hz, J2 = 2.6 Hz, 1H), 7.42-7.28 (m, 5H), 6.56 (d, J = 9.4 Hz, 1H), 5.47 (dd, J1 = 8.8 Hz, J2 = 3.2 Hz, 1H), 4.51 (tdd, J1 = 9.6 Hz, J2 = 5.6 Hz, J3 = 1.3 Hz, 1H), 3.99-3.84 (m, 1H), 3.01-2.88 (m, 1H), 2.42-2.30 (m, 1H). $^{13}C\{1H\}$ NMR (101 MHz, CHLOROFORM-*D*) δ 150.0, 139.8, 136.3, 133.5, 129.4, 128.6, 127.8, 125.9, 123.6, 115.3, 68.9, 51.9, 26.8. HRMS (ESI^{+}) $C_{15}H_{14}N_{3}O_{4}^{+}$ $[M+H]^{+}$: calc. mass 300.0979, found 300.0986. $[\alpha]_D^{20}$ = -985.6° (*c* 0.010, CH_2Cl_2).

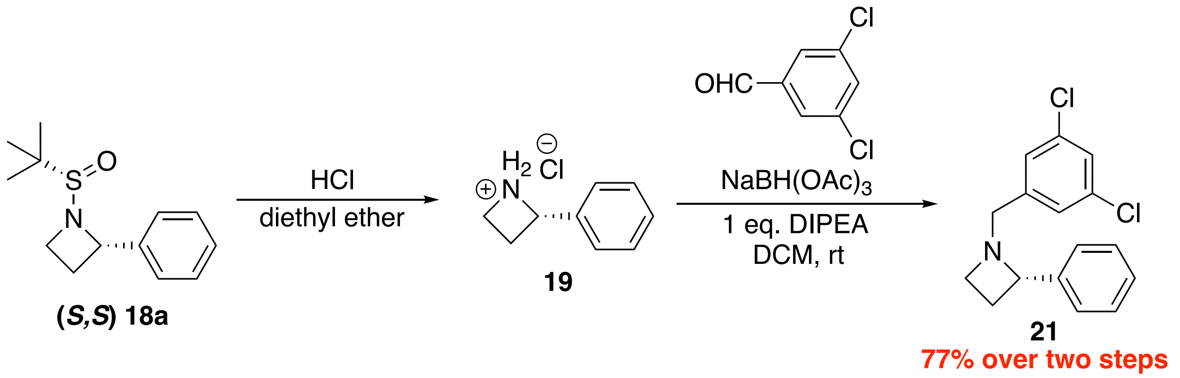

A round-bottomed flask equipped with a magnetic stir bar was charged with azetidine **18a** (100 mg, 0.421 mmol, 1.0 eq) and diethyl ether (8.43 mL, 0.05 M). The solution was stirred and was cooled in an ice bath. 4 M HCl in dioxane was added to the solution via syringe (0.42 mL, 1.69 mmol, 4.0 eq.), and the reaction immediately became cloudy. Within a minute, the ice bath was removed, and it was allowed to stir at room temperature for an additional 30 minutes.

After 30 minutes, the reaction was diluted with 10 mL of diethyl ether, and it was passed through a syringe with a cotton plug. The round-bottomed flask was washed an additional two times with diethyl ether, and the solutions were also passed through the syringe filter (2 X 10 mL). The filtrate was colorless and was discarded. The filtercake was dissolved with dichloromethane, and the solution was passed through the filter and was collected separately. It was concentrated under reduced pressure, and this provided azetidine hydrochloride **19** as an off-white solid. Its tabulated spectra are described two pages before this one.

To a round bottomed flask equipped with a magnetic stir bar was added the crude azetidine hydrochloride salt **19**, which was dissolved in dichloromethane (2.1 mL, 0.2 M). 3,5-dichlorobenzaldehyde was added to the mixture as a solid (111 mg, 0.632 mmol, 1.5 eq) and diisopropylethylamine (73 µL, 0.421 mmol, 1.0 eq) were added to the mixture followed by sodium triacetoxyborohydride (288 mg, 1.264 mmol, 3.0 eq). The reaction was stirred for 18 hours at room temperature.

The reaction mixture was partitioned between ethyl acetate (30 mL) and saturated bicarbonate (50 mL). The organic was extracted from the mixture with ethyl acetate (3 X 30 mL), and the combined organic was washed with saturated brine (50 mL), dried with sodium sulfate, filtered, and concentrated under reduced pressure. The crude was purified by flash column chromatography (gradient 0% → 20% ethyl acetate in hexanes), providing the desired product, which coeluted with the excess aldehyde starting material. To remove the aldehyde, the mixture was dissolved in diethyl ether (15 mL), and 200 uL of 4 M HCl in dioxane was added to

the mixture. This caused the desired product **21** to precipitate as a hydrochloride salt, which was separated by filtration. The solid was then free-based using an extraction, where it was partitioned between ethyl acetate and saturated sodium bicarbonate. The ethyl acetate layer was collected, dried with sodium sulfate, filtered, and concentrated to provide the desired product **21** (95 mg, 77% yield). ^{1}H NMR (400 MHz, CHLOROFORM-*D*) δ 7.47-7.43 (m, 2H), 7.38-7.33 (m, 2H), 7.29-7.24 (m, 1H), 7.21 (s, br, 3H), 4.13 (t, J = 8.1 Hz, 1H), 3.76 (d, J = 13.6 Hz, 1H), 3.43-3.35 (m, 2H), 2.86 (ddd, J1 = 9.7 Hz, J2 = 7.7 Hz, J3 = 6.5 Hz, 1H), 2.34 (dtd, J1 = 9.5 Hz, J2 = 7.5 Hz, J3 = 2.0 Hz, 1H), 2.15 (tt, J1 = 10.0 Hz, J2 = 8.1 Hz, 1H). ^{13}C{1H} NMR (101 MHz, CHLOROFORM-*D*) δ 142.9, 142.2, 134.7, 128.4, 127.5, 127.1, 127.0, 126.8, 69.2, 61.2, 61.4, 27.8. HRMS (ESI^{+}) $C_{16}H_{16}NCl_2^{+}$ [M+H]$^{+}$: calc. mass 292.0654, found 292.0652. $[\alpha]_D^{20}$ = -77.6° (*c* 0.010, CH_2Cl_2).

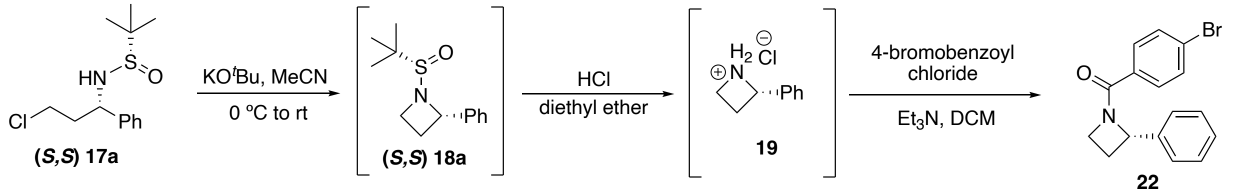

A round bottomed flask equipped with a magnetic stir bar was charged with **17a** (140 mg, 0.511 mmol, 1.0 eq), and was dissolved in acetonitrile (5.11 mL, 0.1 M). The solution was cooled in an ice bath, potassium tert-butoxide was added (86 mg, 0.767 mmol, 1.5 eq), and the reaction was removed from the ice bath. It was stirred at room temperature for one hour. The reaction was diluted with diethyl ether (25 mL), and it was passed through a syringe with a cotton plug into a round bottomed flask. The reaction flask was washed out with additional diethyl ether, which was then passed through the syringe filter (2 X 12.5 mL). This produced a clear solution of roughly 55 mL, and this solution was stirred on a magnetic stir plate and was cooled in an ice bath. HCl was added to the reaction (4 M solution in dioxane, 0.51 mL, 2.045 mmol, 4.0 eq), and it immediately became cloudy. After two minutes, it was removed from the ice bath and was left at room temperature to stir for an additional 30 minutes. This led to an insoluble residue coating the bottom of the flask (compound **19**). The diethyl ether was decanted, leaving the residue behind, and the residue was further washed with diethyl ether (2 X 25 mL). The residue was then dissolved in dichloromethane (5.11 mL, 0.1 M) in preparation for acid chloride acylation. The solution was cooled in an ice bath and triethylamine was added (0.53 mL, 3.835 mmol, 7.5 eq), which free-based the amine, producing a clearer solution. To this stirred solution was added the 4-bromobenzoyl chloride (280.5 mg, 1.278 mmol, 2.5 eq) as a solid, and this led to almost immediate formation of a precipitate, and the reaction was stirred for an additional 30 minutes. The reaction was then diluted with ethyl acetate (50 mL), which was used to facilitate transfer to a separatory funnel. The organic layer was washed with saturated sodium bicarbonate (2 X 30 mL), 1 M HCl (2 X 30 mL), and saturated brine (30 mL). The organic layer was dried with sodium sulfate, filtered, and concentrated under reduced pressure. The crude material was purified by flash column chromatography (gradient 15% → 30% ethyl acetate in hexanes), yielding the desired product (white solid, 77 mg, 48% yield). ^{1}H NMR (400 MHz, CHLOROFORM-*D*) at room temperature δ 7.65-7.10 (m, 9H), 5.70-5.25 (m, 1H), 4.54-4.08 (m, 2H), 2.83 (s, br, 1H), 2.40-2.13 (m, 1H). NMR at 55 °C: δ 7.55-7.22 (m, 9H), 5.48 (s, br, 1H), 4.45-4.35 (m, 1H), 4.30-4.17 (m, 1H), 2.90-2.75 (m, 1H), 2.35-2.20 (m, 1H). ^{13}C{1H} NMR (101 MHz, CHLOROFORM-*D*) at 55 °C: δ 170.4, 142.1, 132.7, 131.6, 129.7, 128.9, 127.9, 126.0, 125.6, 65.2 (br), 49.5 (br), 26.2. HRMS (ESI$^+$) $C_{16}H_{15}BrNO^+$ $[M+H]^+$: calc. mass 316.0332, found 316.0330. $[\alpha]_D^{20}$ = -127 (*c* 0.018, CH_2Cl_2).

Determination of Diastereoselectivity

The Diastereomeric ratios (*dr*) of the chlorosulfinamides and azetidines were calculated through ^{1}H NMR, quantitative ^{13}C NMR (increased relaxation time between pulses), and ^{19}F NMR when possible. NMR data was acquired of the crude reaction mixtures as well as different sets of fractions during purification, and this allowed us to determine the *dr* of the reaction, and whether the diastereomers were separable by column chromatography. To illustrate this process, we show an example below where ^{1}H NMR, quantitative ^{13}C NMR, and ^{19}F NMR are all used to examine *dr*, and the data demonstrate that all spectral options provide very similar values for *dr*.

To provide an example of this, we examine the preparation of 4-fluorophenyl chlorosulfinamide **17b**. A crude ^{1}H NMR, quantitative ^{13}C NMR, and ^{19}F NMR were taken before purification, and various sets of fractions were evaluated after purification to compare the signals to determine *dr*. To demonstrate the agreement between the different NMR data, we show below different NMR signals from various samples to demonstrate consistency between the NMR methods.

Below, we show a fraction set that was close to a 1:1 ratio, a fraction that was >90:10, a fraction that was >95:5, and the crude mixture, which was 85:15.

The set of fractions below (**Figure S1**) shows close to a 1:1 ratio between the two diastereomers. Through ^{19}F NMR, two major signals were observed, and each were integrated, giving values of 1.00 and 1.27. These signals represented the same fluorine highlighted in red on the two different diastereomers. Likewise, quantitative ^{13}C NMR was used to examine the DR, shown by the two middle panes and carbons in red. The minor diastereomer signals were set to an integration value of 1.00 and the major subsequently integrated to 1.26 and 1.27, respectively. Finally, ^{1}H NMR was used to integrate the most identifiable signal in the spectra, the *tert*-butyl protons. The minor diastereomer integrated to 1.00 and the major to 1.25. Through these integrated values from the three NMR experiments, a *dr* of 44:56 was recorded for this set of fractions.

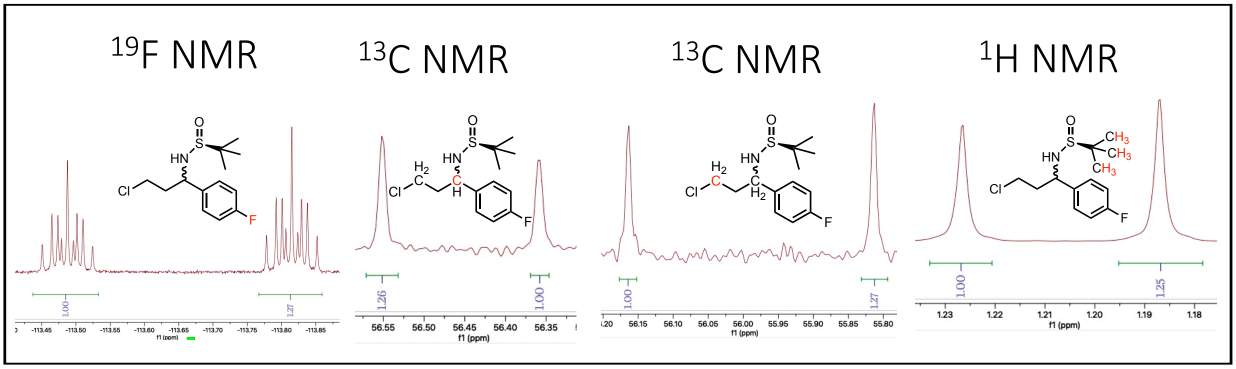

Figure S1. Comparison of NMR data from sample with close to a 1:1 ratio between diastereomers. A fluorine, quantitative carbon, and proton NMR of the mixed fractions are shown above. The ratios between the diastereomers range from 1:1.25 to 1:1.27, which demonstrates a close degree of agreement between the various NMR methods.

The set of fractions below (**Figure S2**) shows a 90:10 ratio between the two diastereomers. Through ^{19}F NMR, two major signals were observed, and each were integrated, giving values of 1.00 and 0.09. These signals represented the same fluorine highlighted in red on the two different diastereomers. Likewise, quantitative ^{13}C NMR was used to examine the *dr*, shown by the two middle panes and carbons in red. The major diastereomer signals were both set to an integration value of 1.00, and the minor signals integrated to 0.09 and 0.08. Finally, ^{1}H NMR was used to integrate the most identifiable signal in the spectra, the *tert*-butyl protons. The major diastereomer integrated to 1.00 and the minor to 0.09. Through these integrated values from the three NMR experiments, a *dr* of 90:10 was recorded for this set of fractions.

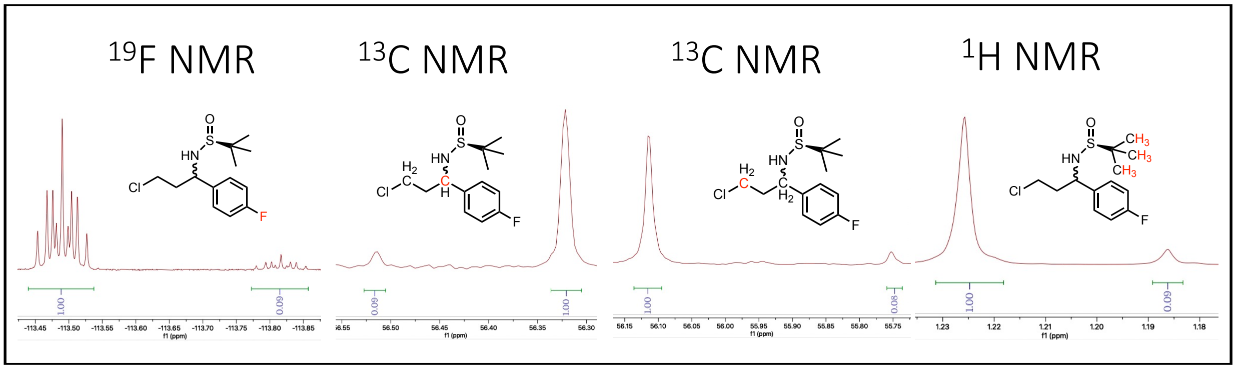

Figure S2. Comparison of NMR data from a sample with an approximately 90:10 ratio between diastereomers. A fluorine, quantitative carbon, and proton NMR of the mixed fractions are shown above. The ratios between the diastereomers range from 1:0.08 to 1:0.09, which demonstrates a close degree of agreement between the various NMR methods.

The set of fractions below (**Figure S3**) shows a >95:5 ratio between the two diastereomers. Through ^{19}F NMR, only the major signal is observed. Likewise, quantitative ^{13}C NMR was used to examine the *dr*, shown by the two middle panes and carbons in red, and only the major carbon signals were observed. Finally, ^{1}H NMR was used to integrate the most identifiable signal in the spectra, the *tert*-butyl protons. Again, only the major diastereomer was observed. Through these integrated values from the three NMR experiments, a *dr* of >95:5 was recorded for this set of fractions.

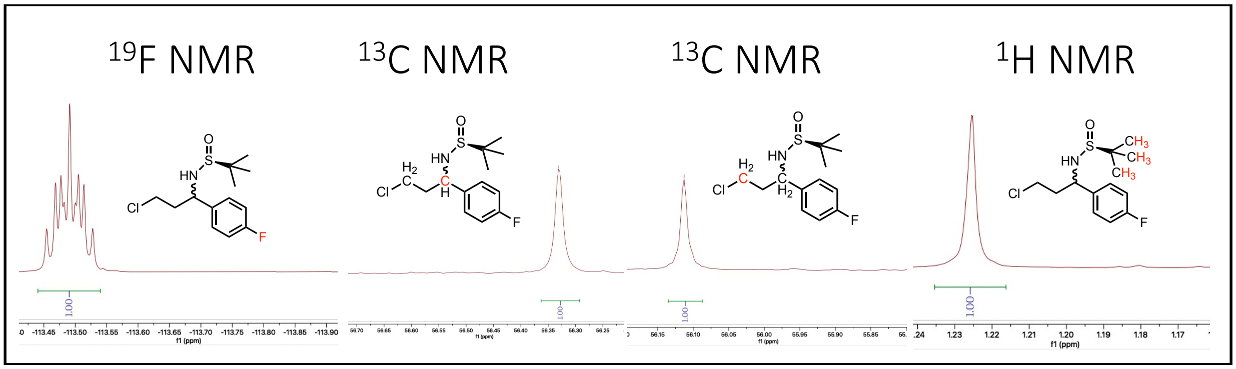

Figure S3. Comparison of NMR data from sample with a >95:5 ratio between diastereomers. A fluorine, quantitative carbon, and proton NMR of the mixed fractions are shown above. Only one diastereomer was recorded in all methods, which demonstrates a close degree of agreement between the various NMR methods.

NMR data of the crude reaction mixture below shows an 85:15 ratio between the two diastereomers. Through ^{19}F NMR, two major signals were observed, and each were integrated, giving values of 1.00 and 0.16. These signals represented the same fluorine highlighted in red on each diastereomers. Likewise, quantitative ^{13}C NMR was used to examine the DR, shown by the two middle panes and carbons in red. The major diastereomers were set to an integration value of 1.00 and the minor signals followed, integrating to 0.15 and 0.15, respectively. Finally, ^{1}H NMR was used to integrate the *tert*-butyl protons. The major diastereomer integrated to 1.00 and the minor to 0.16. Through these integrated values from the three NMR analyses, a diastereomeric ratio of 85:15 was recorded. The NMR data of the crude reaction mixture compared with isolated fractions provided additional certainty that our *dr* calculations were accurate.

In the case of **17b**, the major and minor diastereomers were somewhat separable via column chromatography. However, diastereomers of other chlorosulfinamides (**17**) were not always separable through column chromatography. Note that the splitting pattern on the ^{19}F NMR signal was different, likely due to poor shimming of the sample.

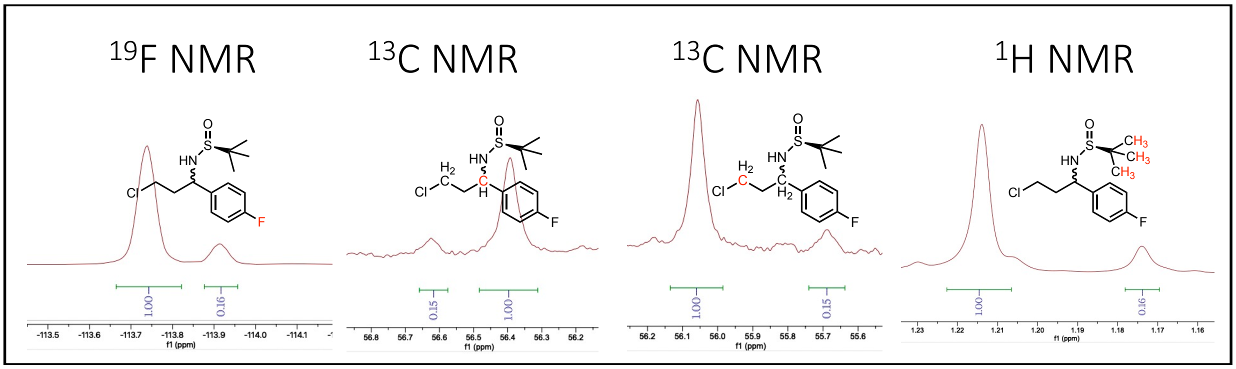

Figure S4. Comparison of NMR data from the crude with close to a 85:15 ratio between diastereomers. A fluorine, quantitative carbon, and proton NMR of the mixed fractions are shown above. The ratios between the diastereomers range from 1:0.15 to 1:16, which demonstrates a close degree of agreement between the various NMR methods.

NMR Spectra:

All spectra were acquired in $CDCl_3$, which is set to 7.26 ppm.

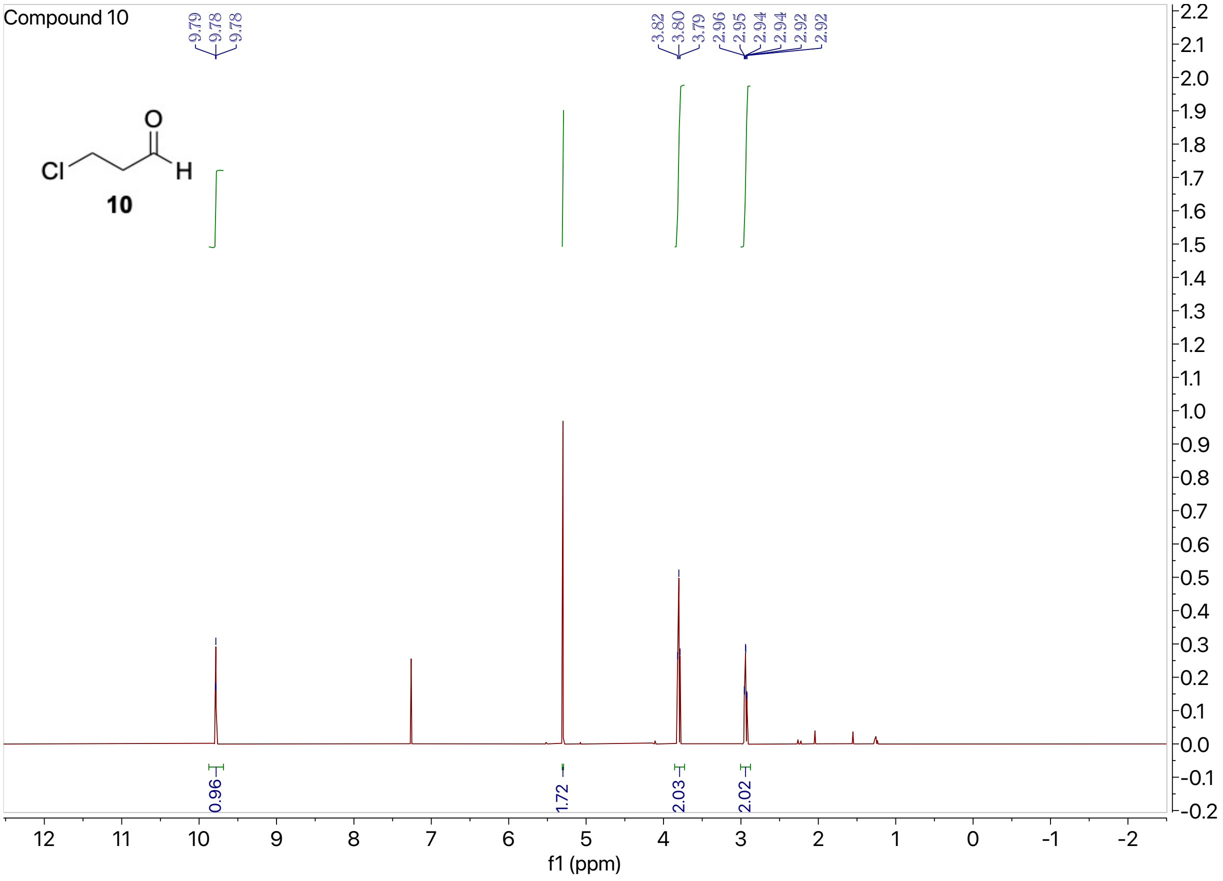

Acquired on a 400 MHz JEOL spectrometer.

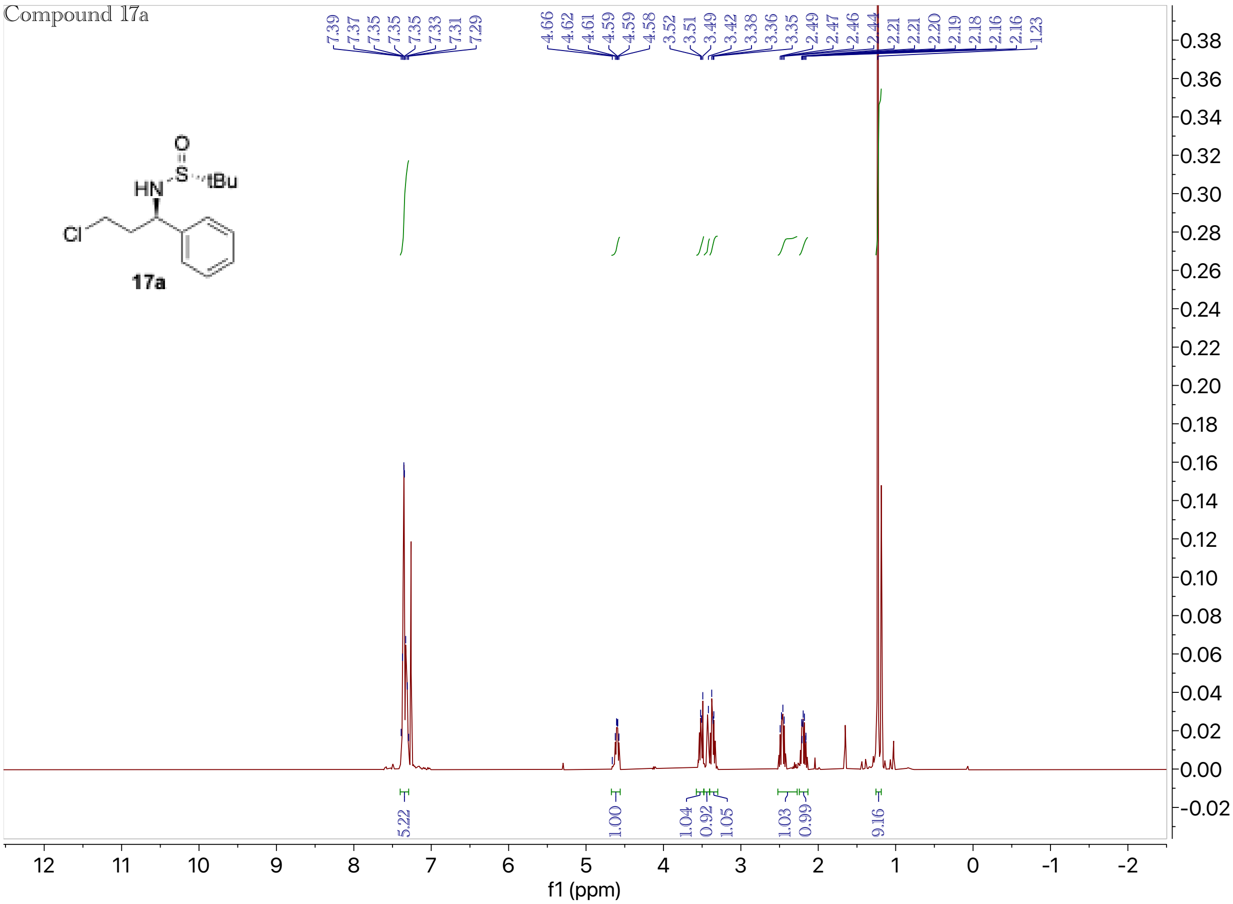

Acquired on a 400 MHz JEOL spectrometer.

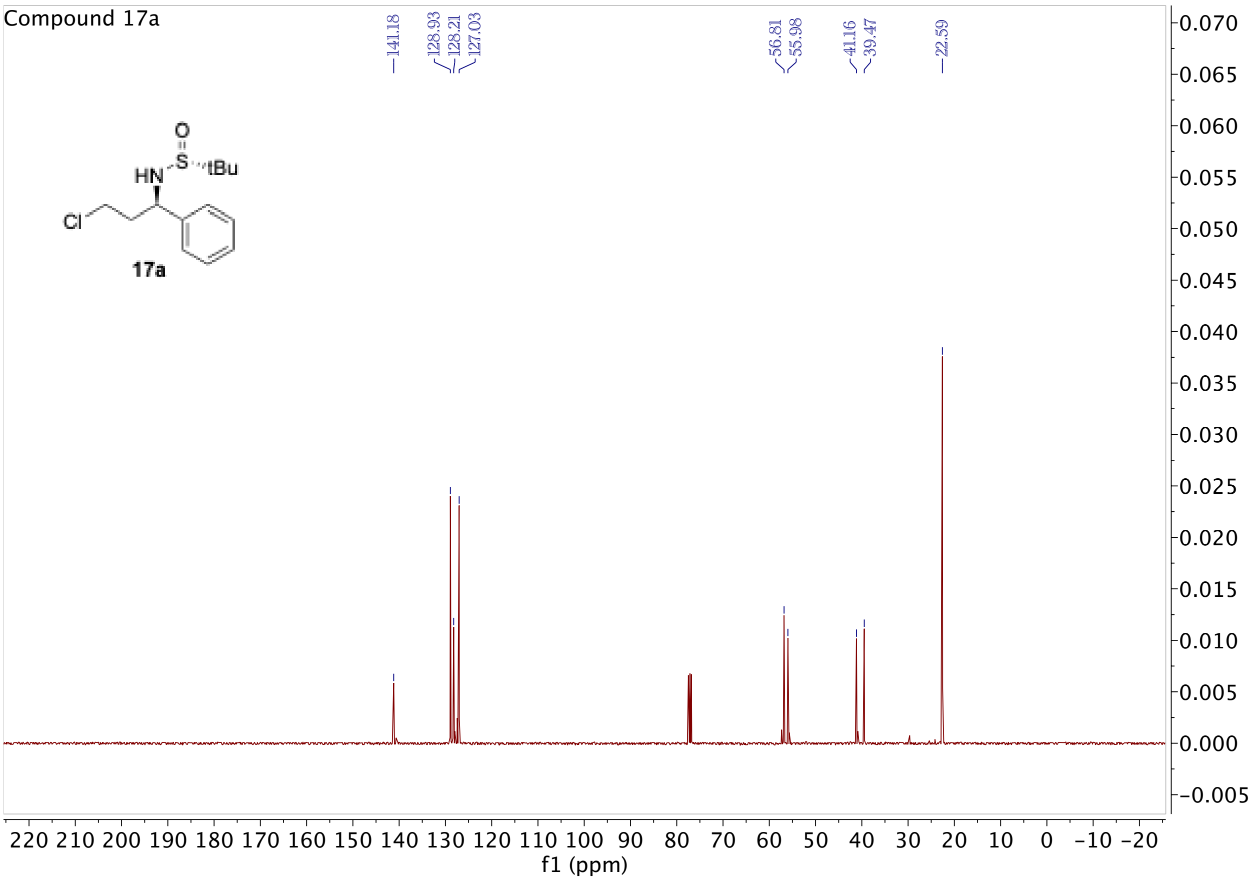

Acquired on a 500 MHz JEOL spectrometer.

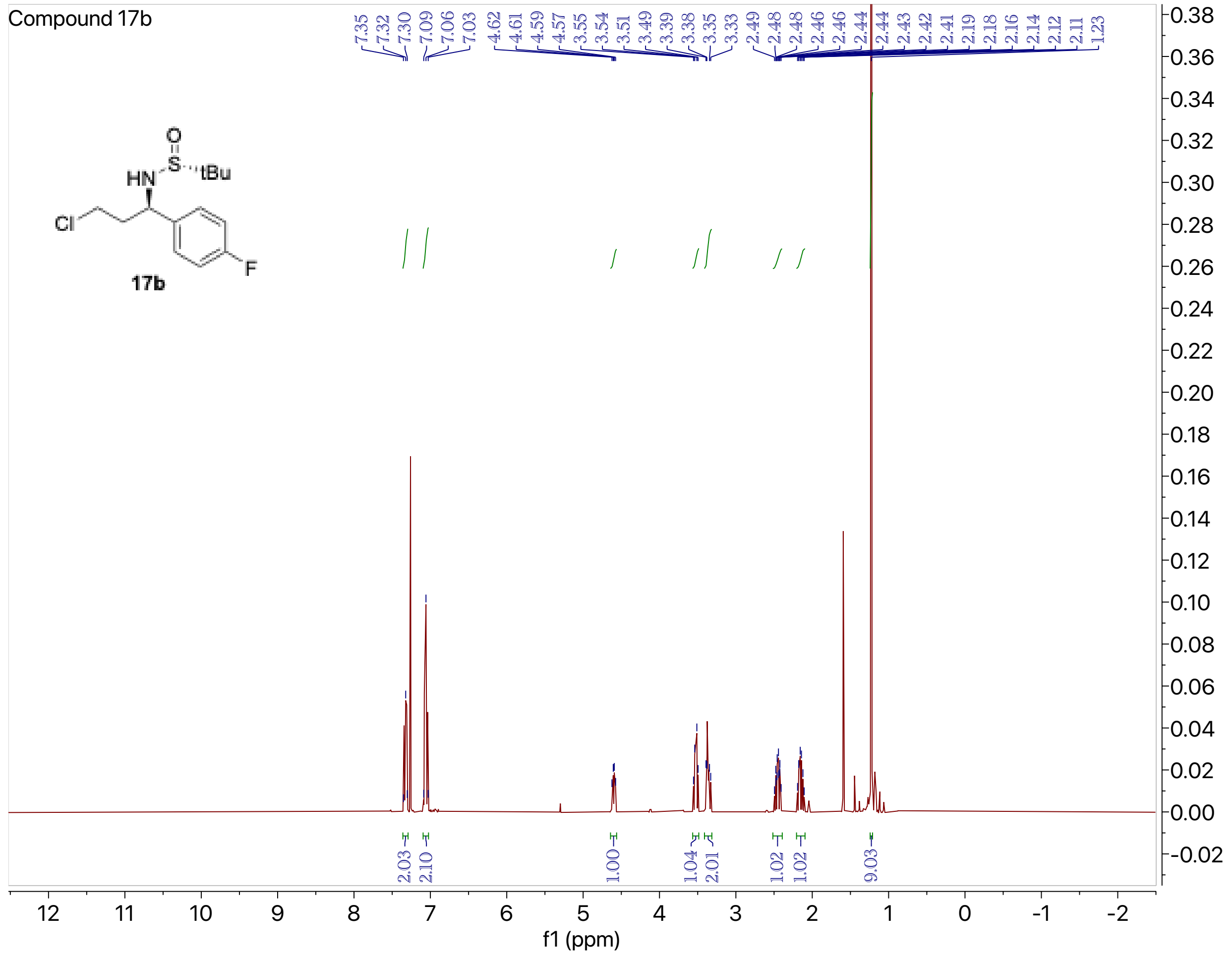

Acquired on a 400 MHz JEOL spectrometer.

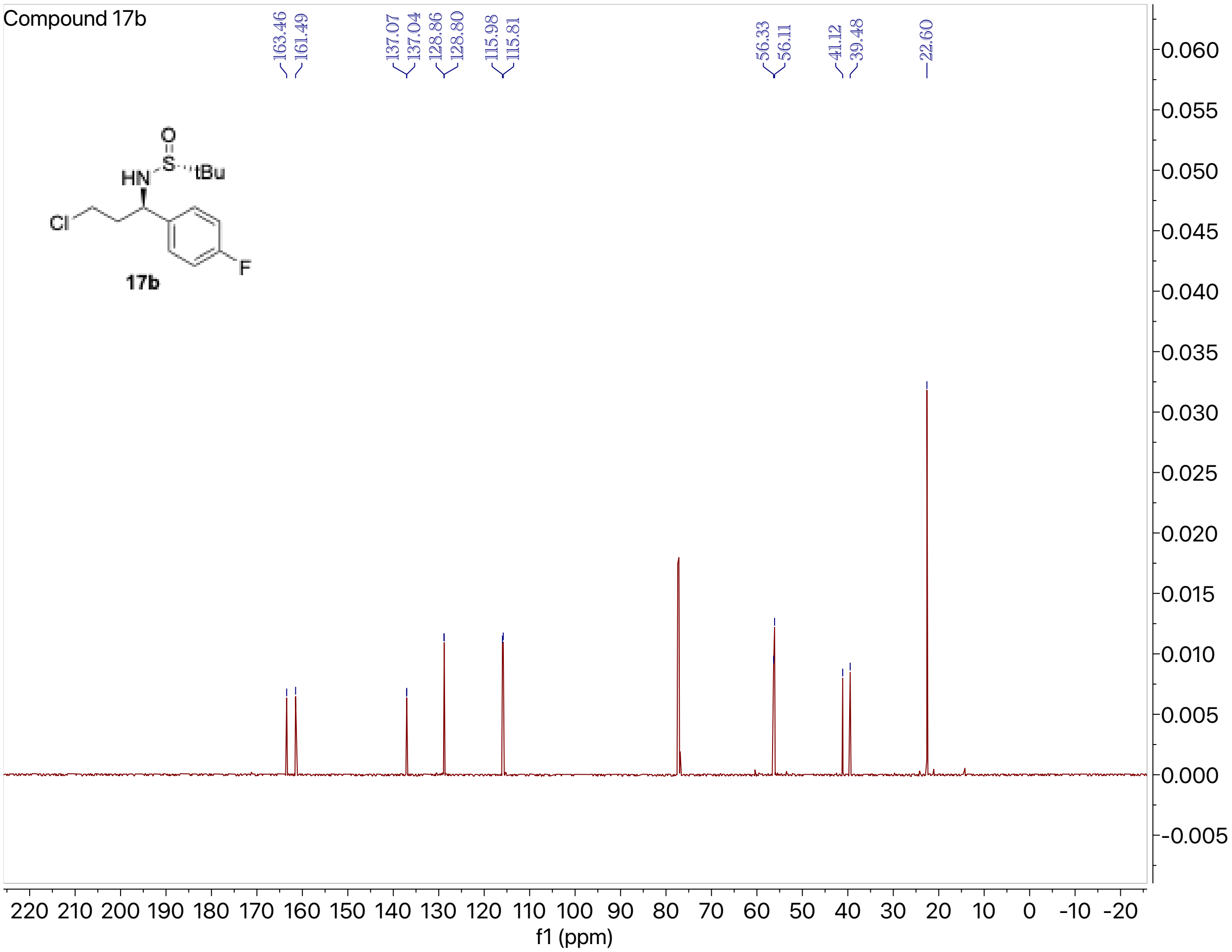

Acquired on a 500 MHz JEOL spectrometer.

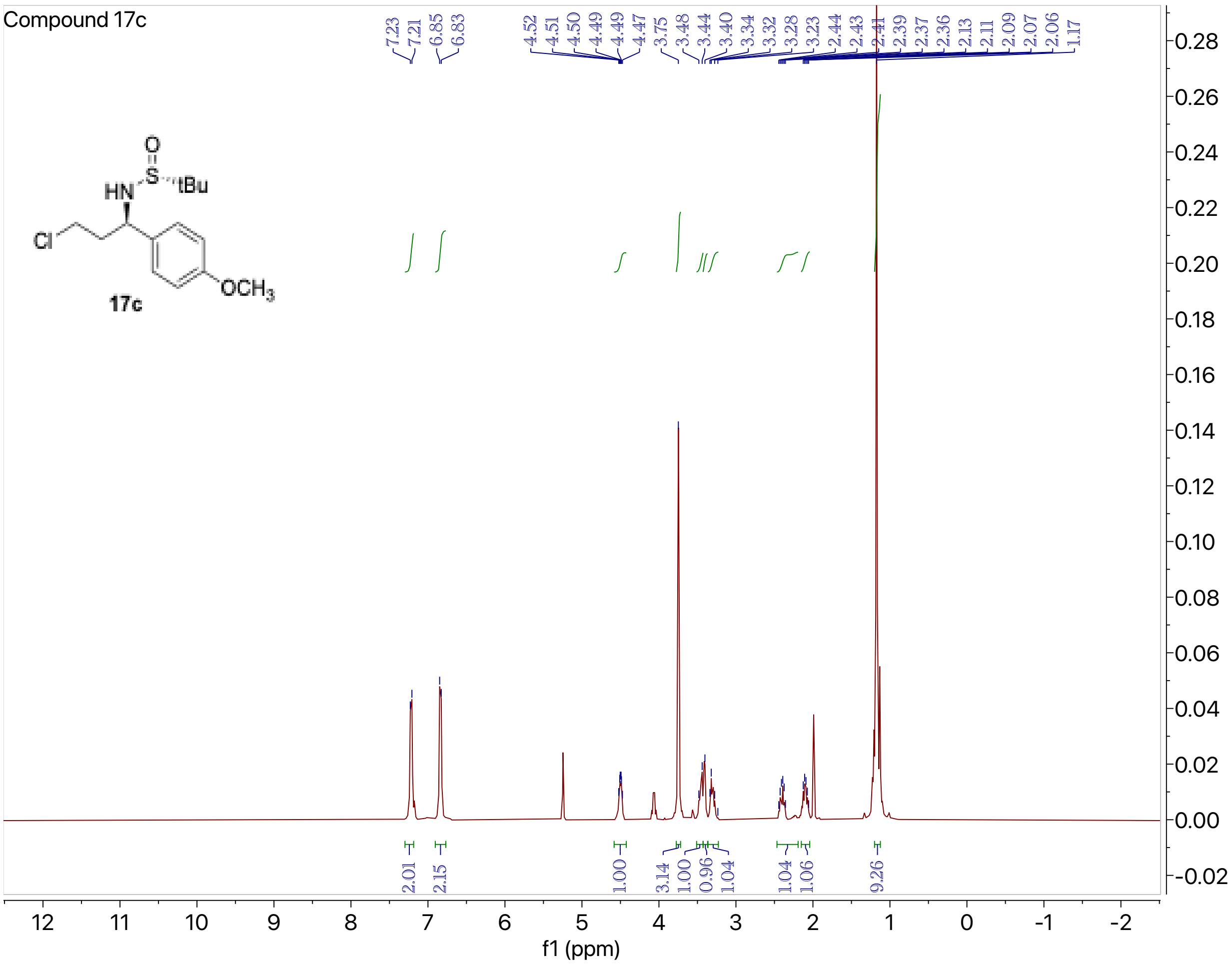

Acquired on a 400 MHz JEOL spectrometer.

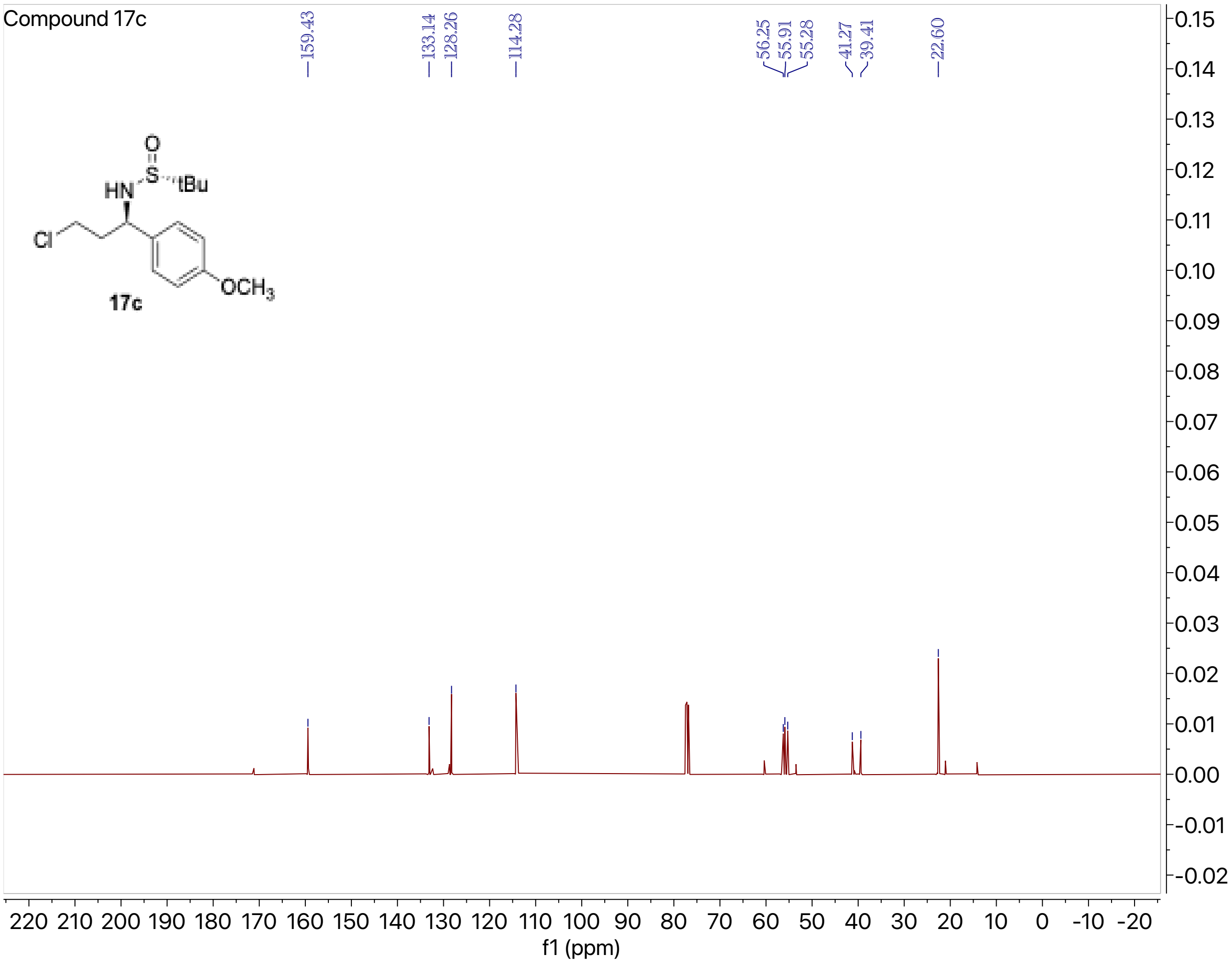

Acquired on a 400 MHz JEOL spectrometer.

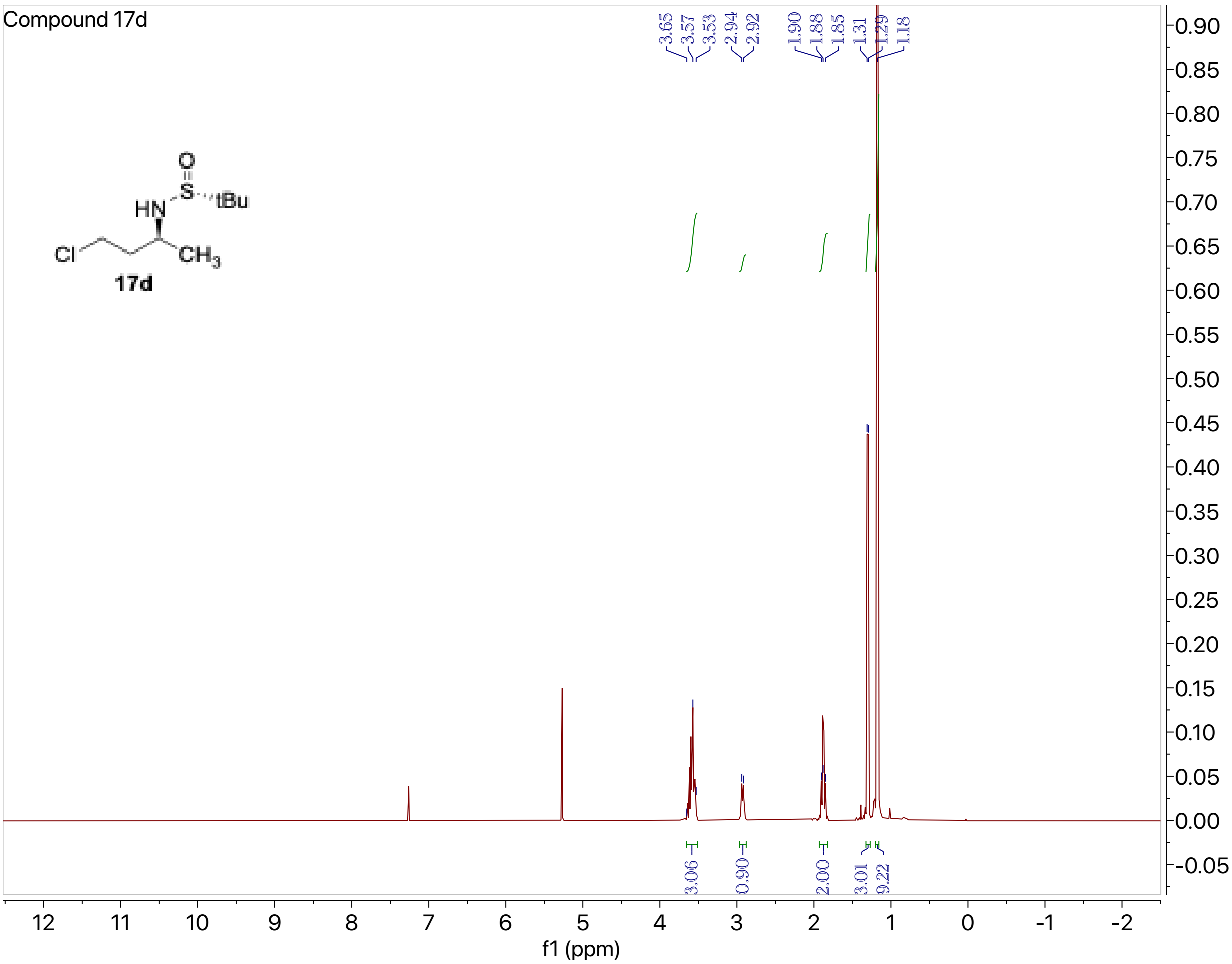

Acquired on a 400 MHz JEOL spectrometer.

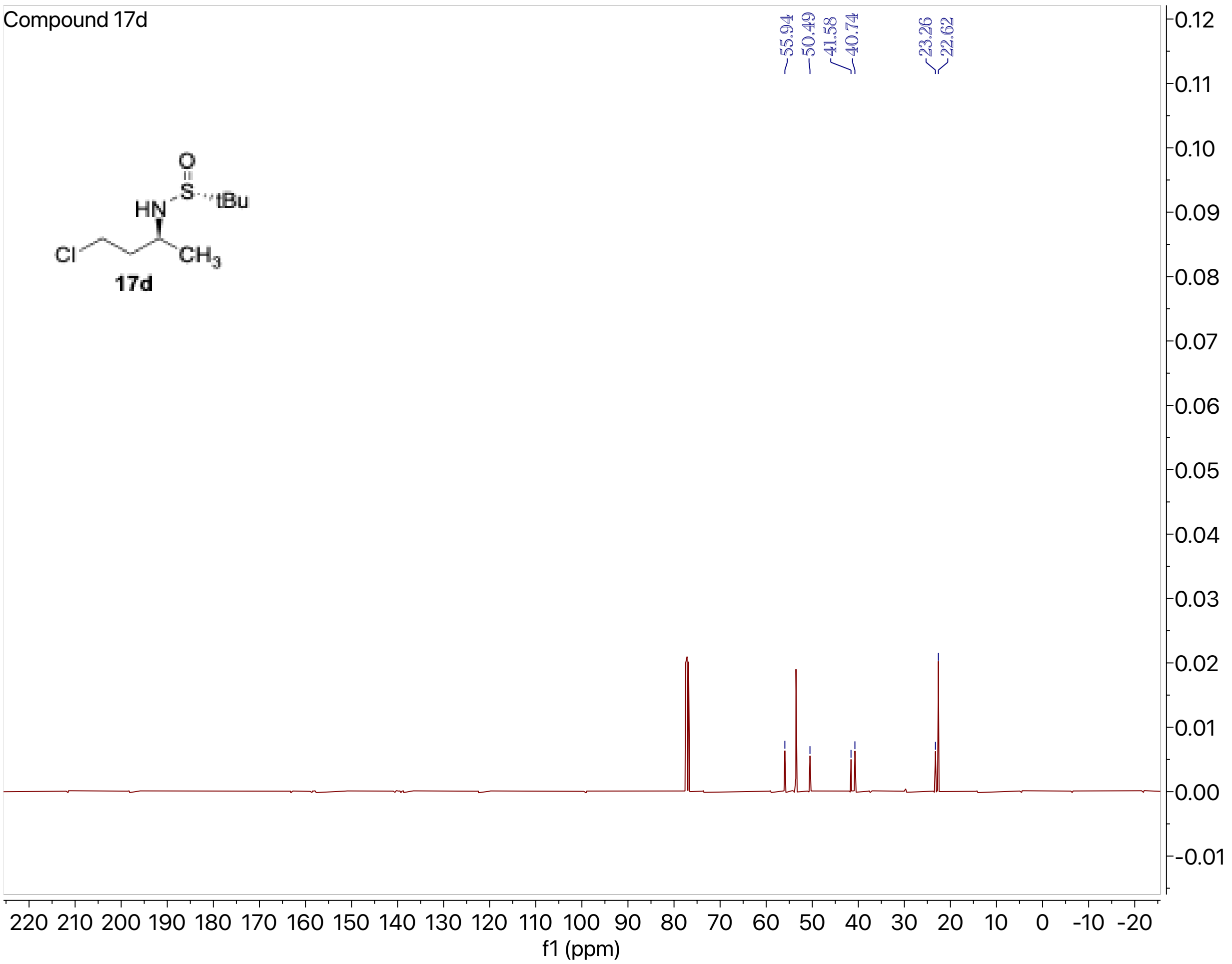

Acquired on a 400 MHz JEOL spectrometer.

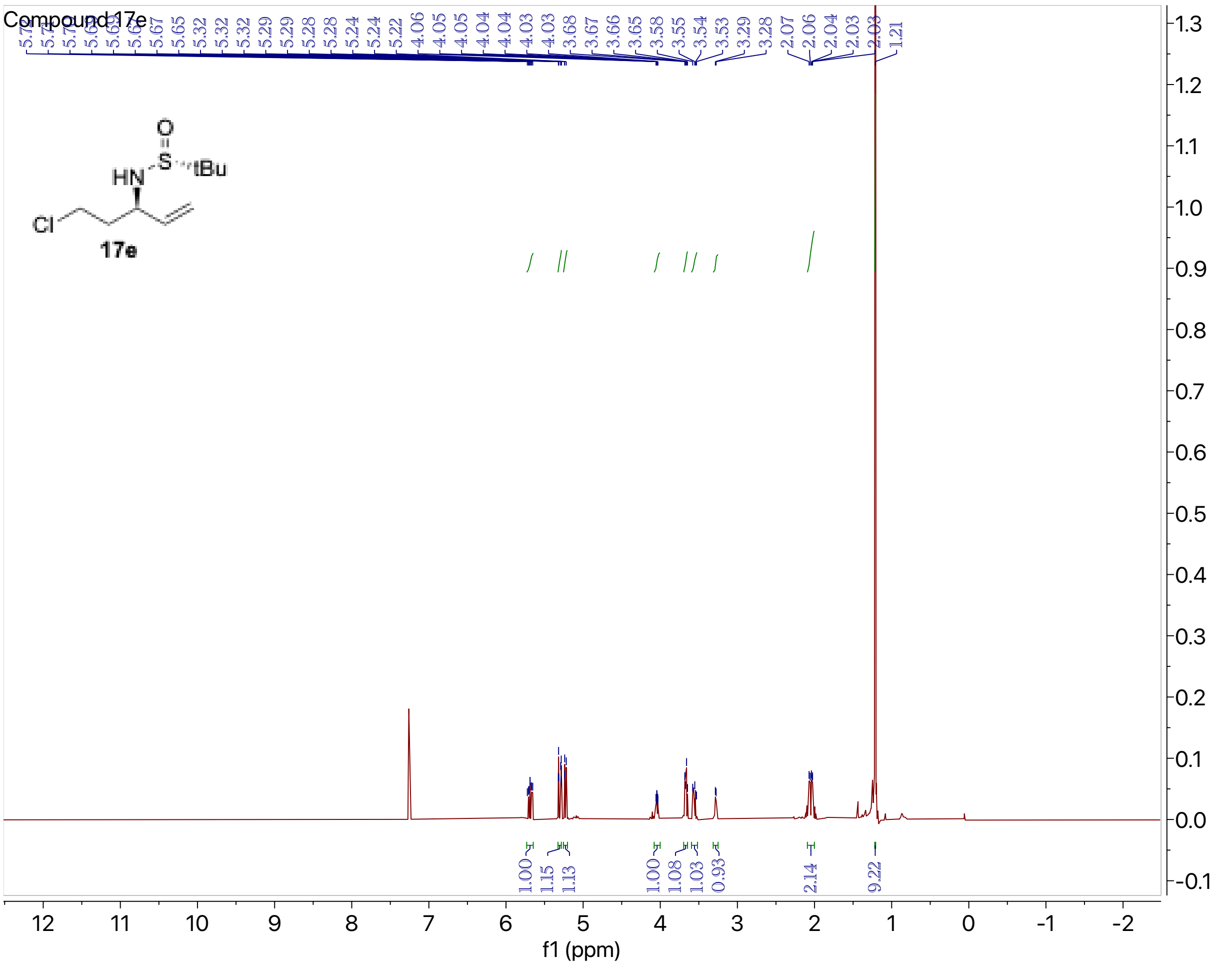

Acquired on a 500 MHz JEOL spectrometer.

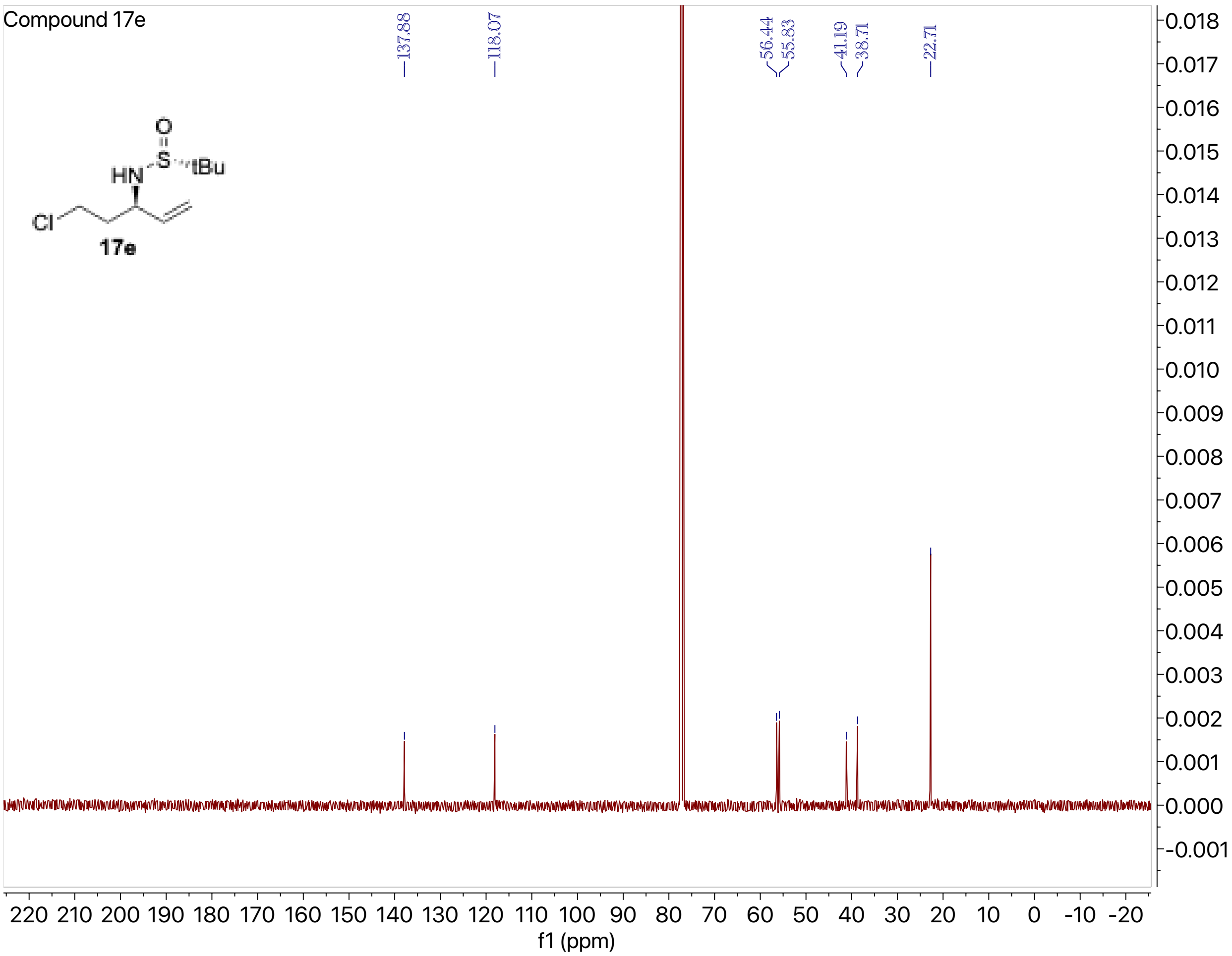

Acquired on a 400 MHz JEOL spectrometer.

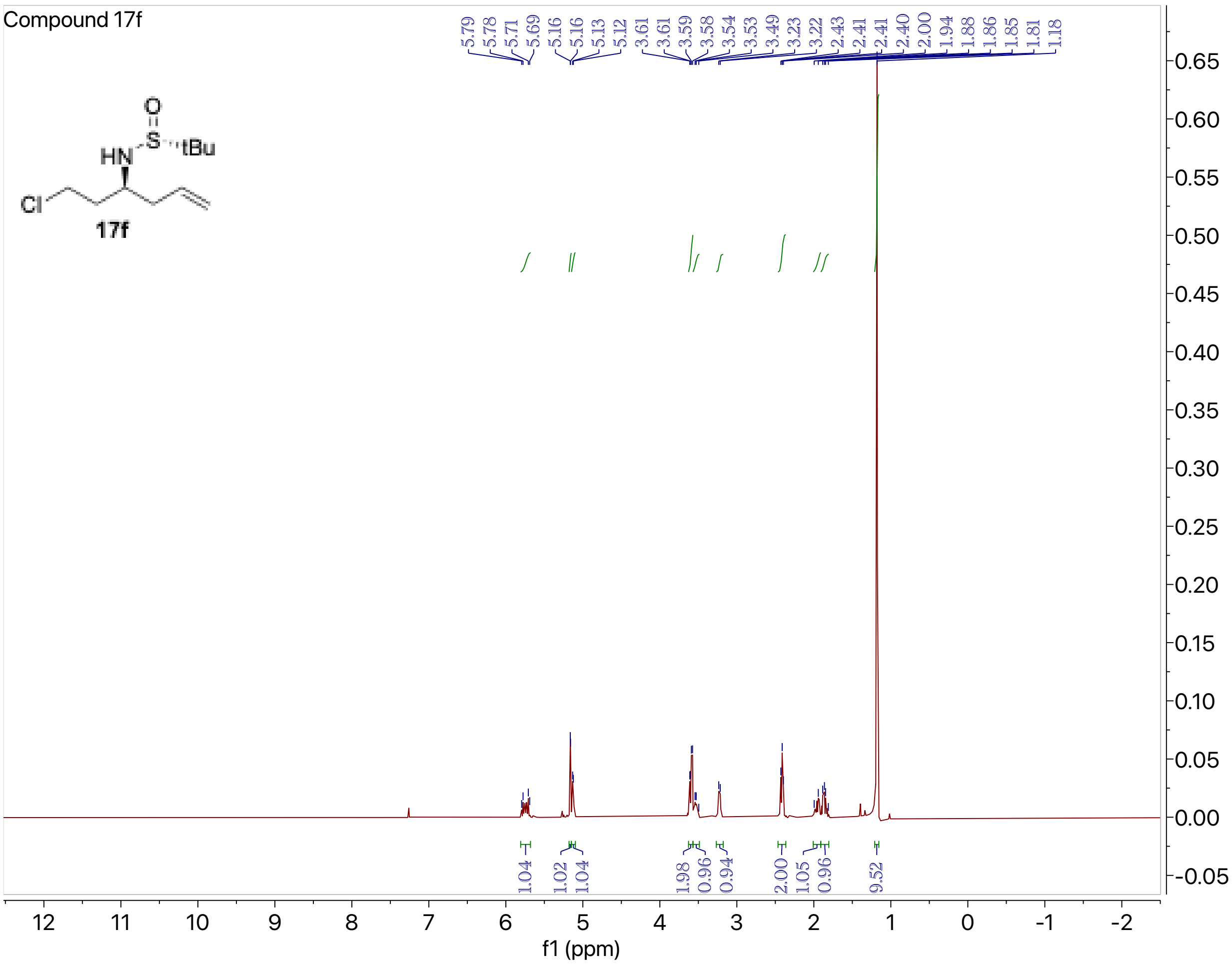

Acquired on a 400 MHz JEOL spectrometer.

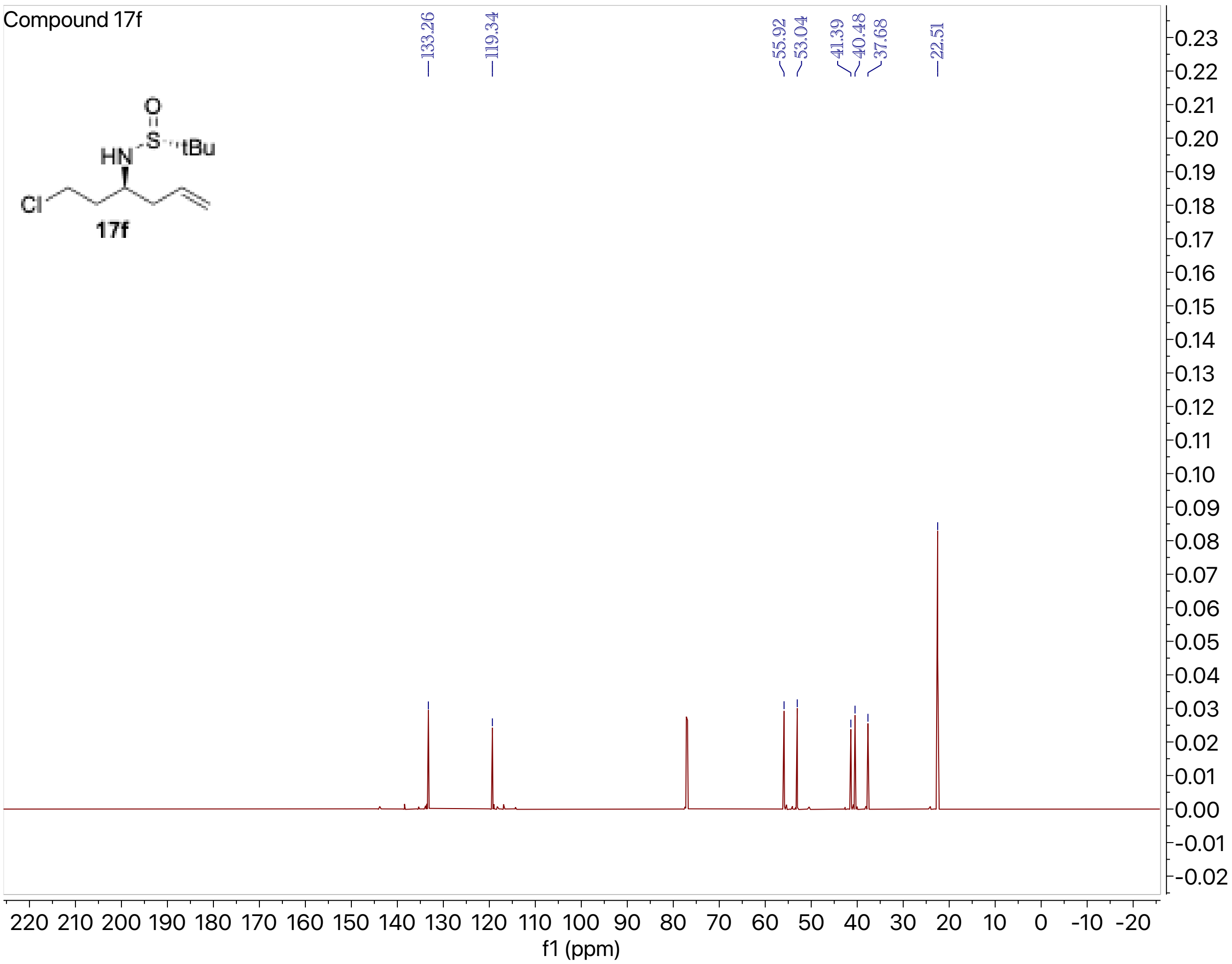

Acquired on a 500 MHz JEOL spectrometer.

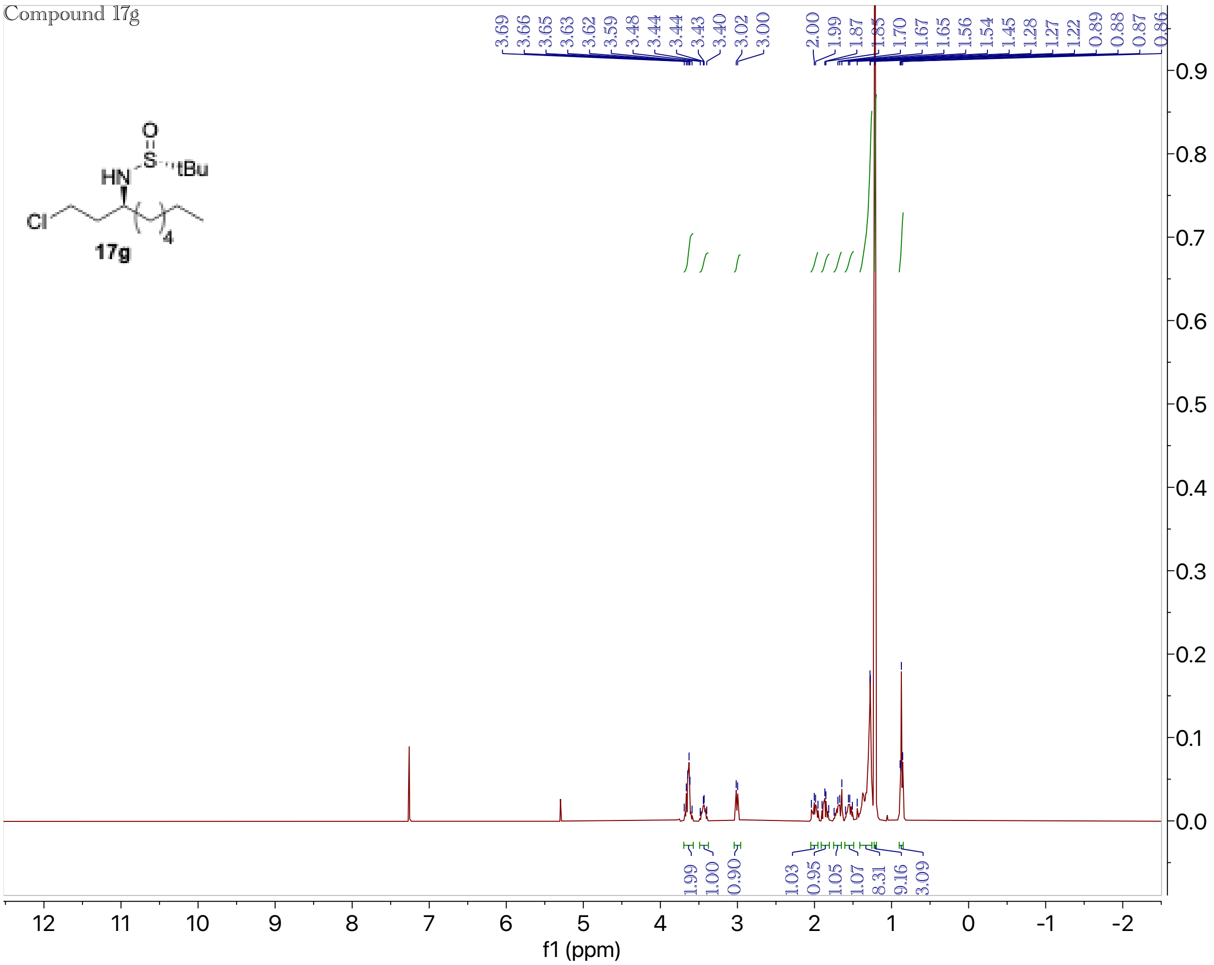

Acquired on a 400 MHz JEOL spectrometer.

Compound 17g

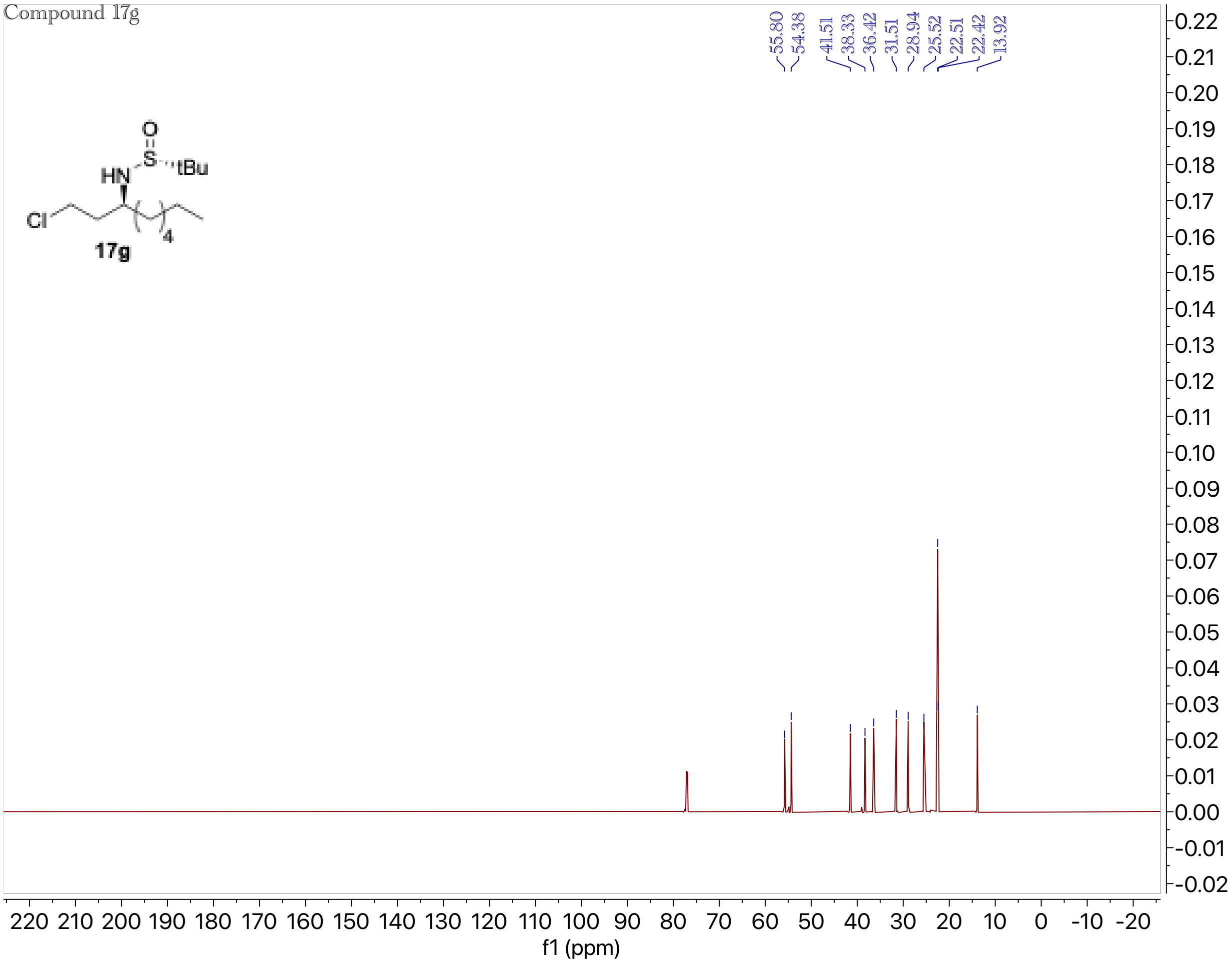

Acquired on a 500 MHz JEOL spectrometer.

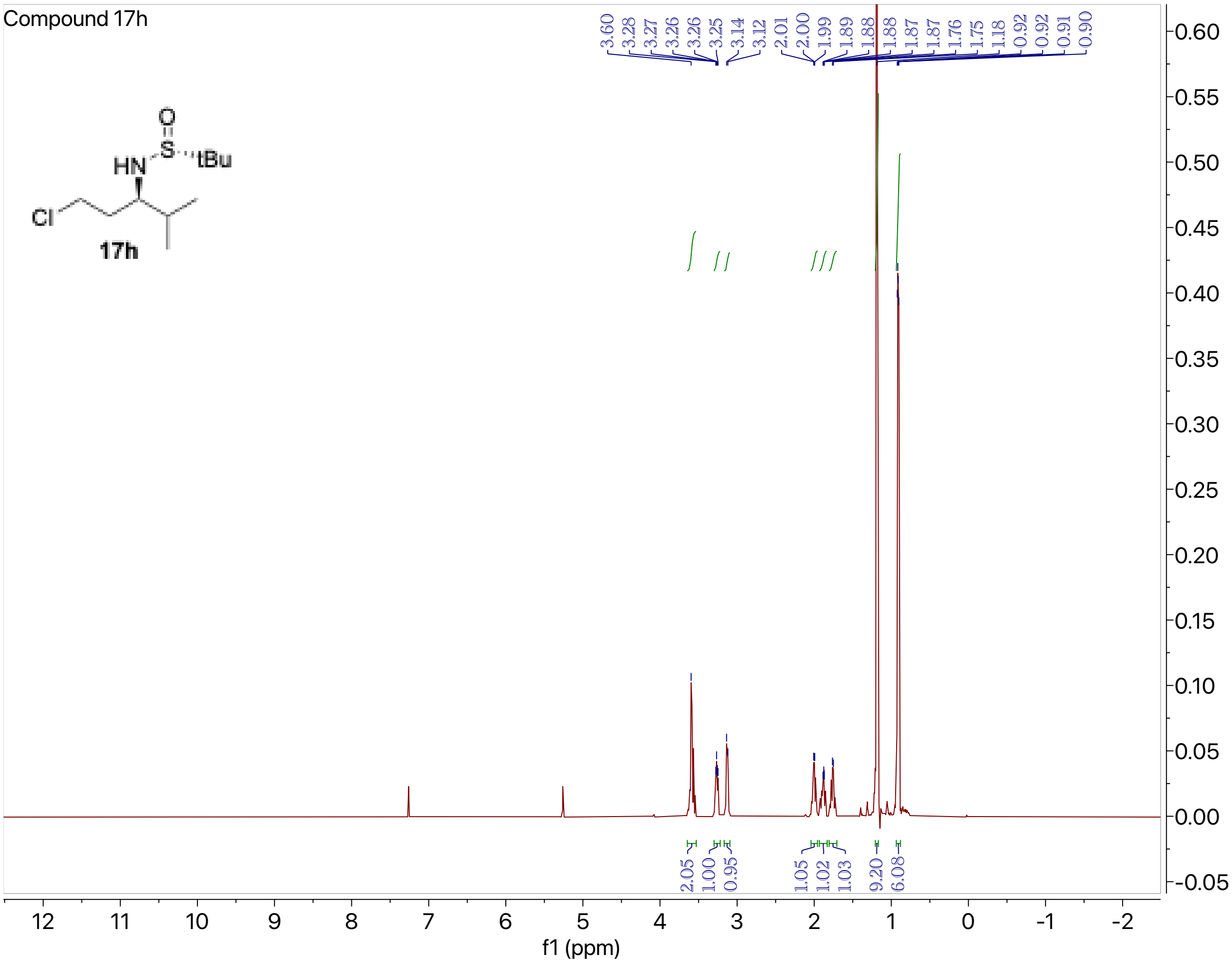

Acquired on a 400 MHz JEOL spectrometer.

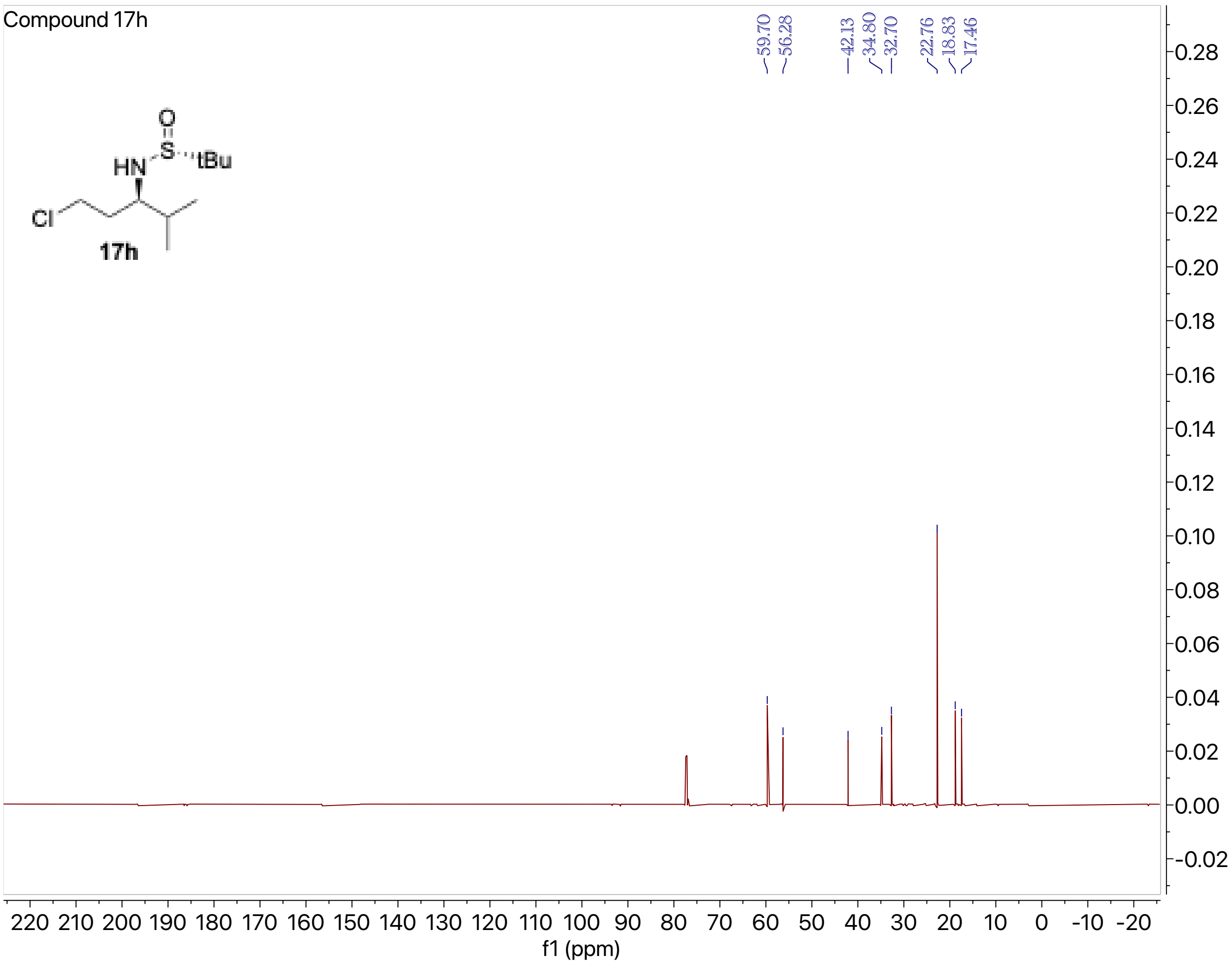

Acquired on a 500 MHz JEOL spectrometer.

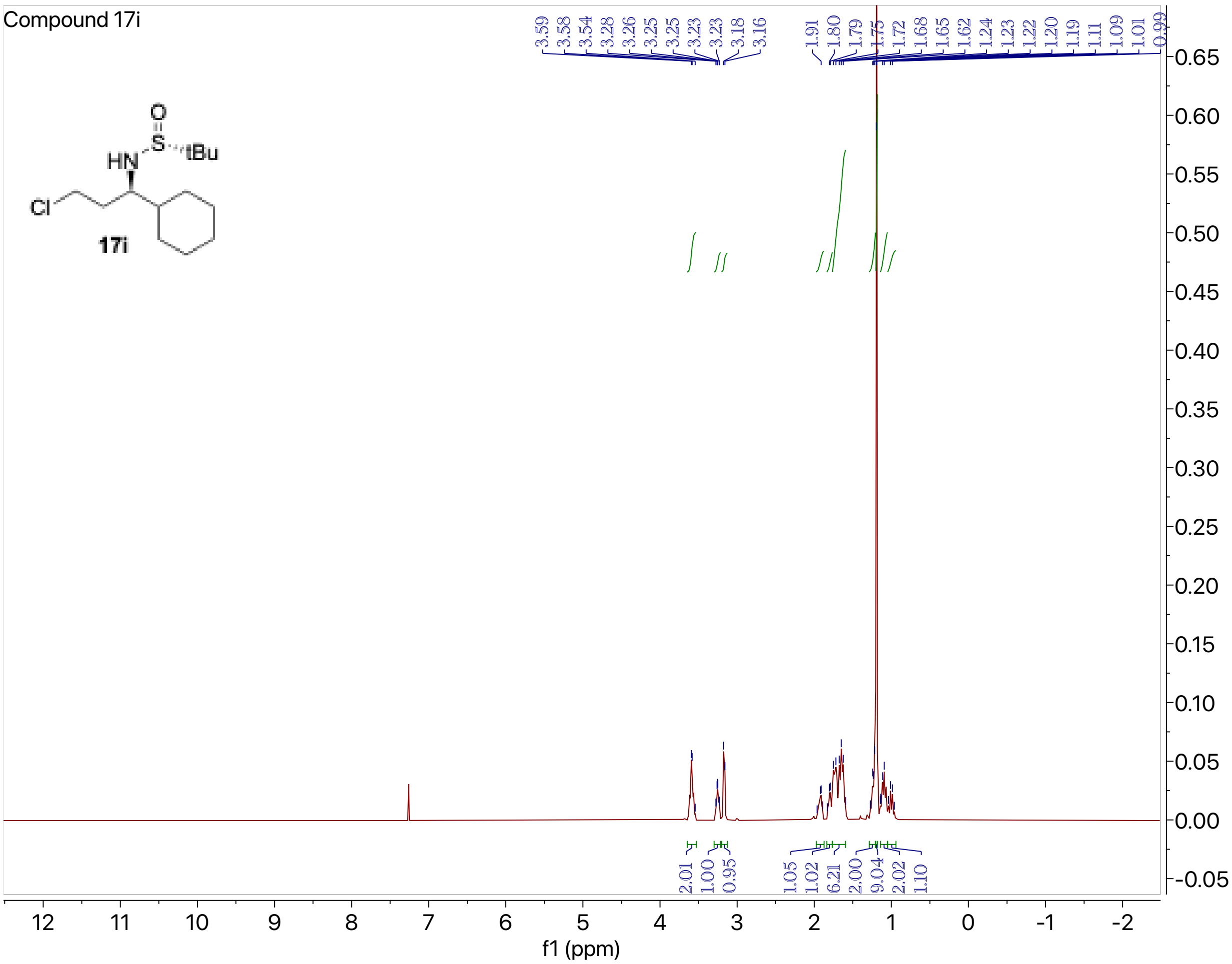

Acquired on a 500 MHz JEOL spectrometer.

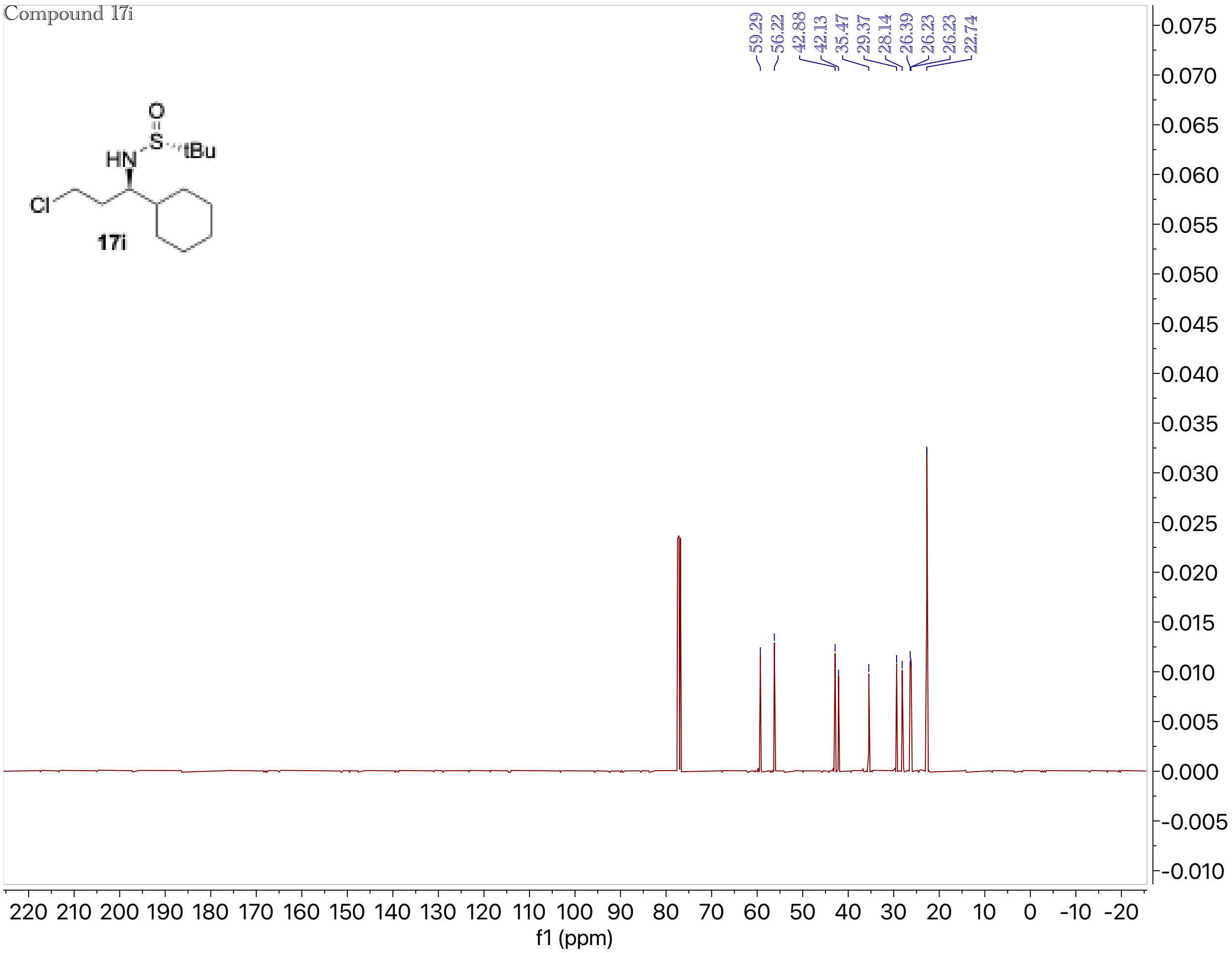

Acquired on a 400 MHz JEOL spectrometer.

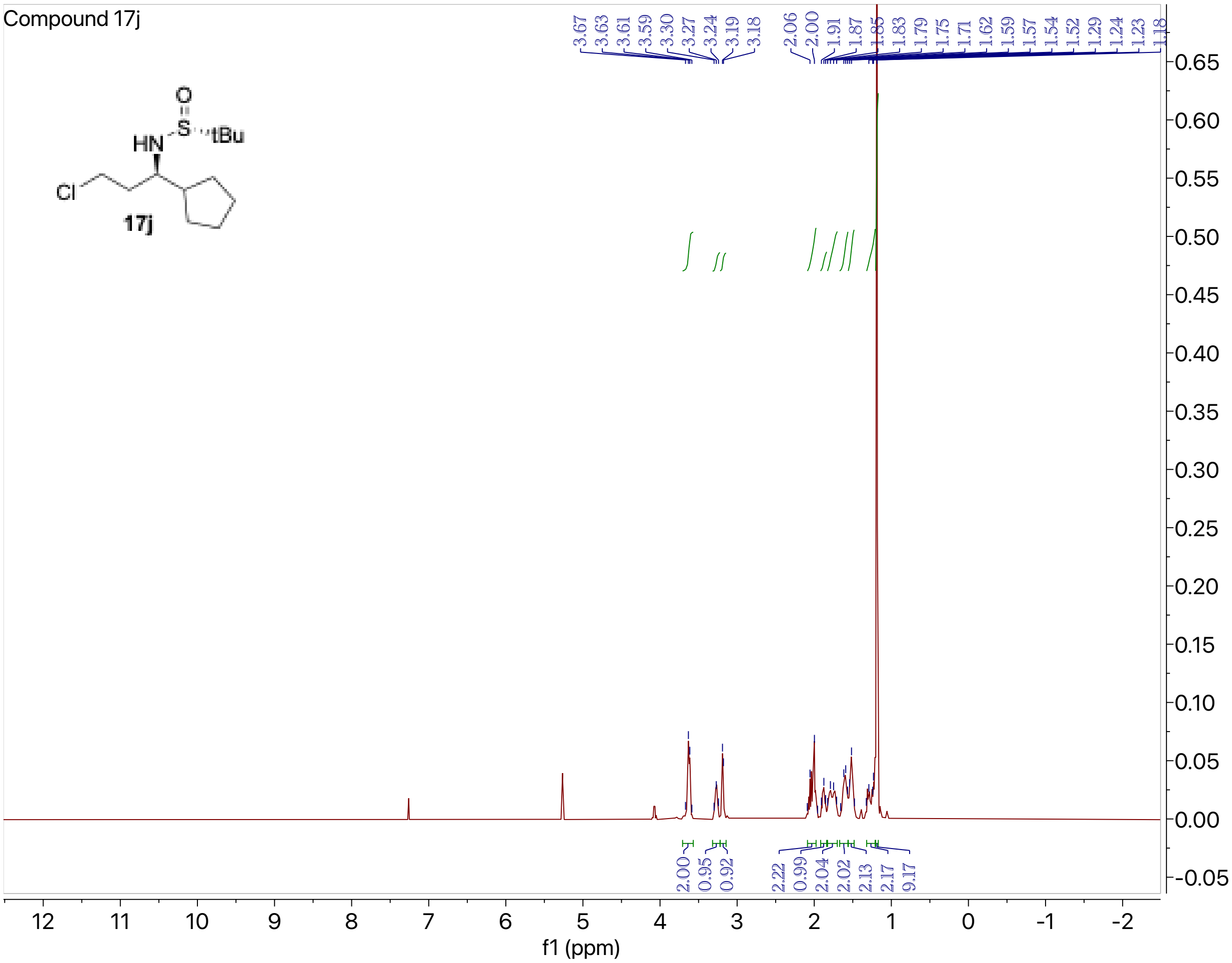

Acquired on a 500 MHz JEOL spectrometer.

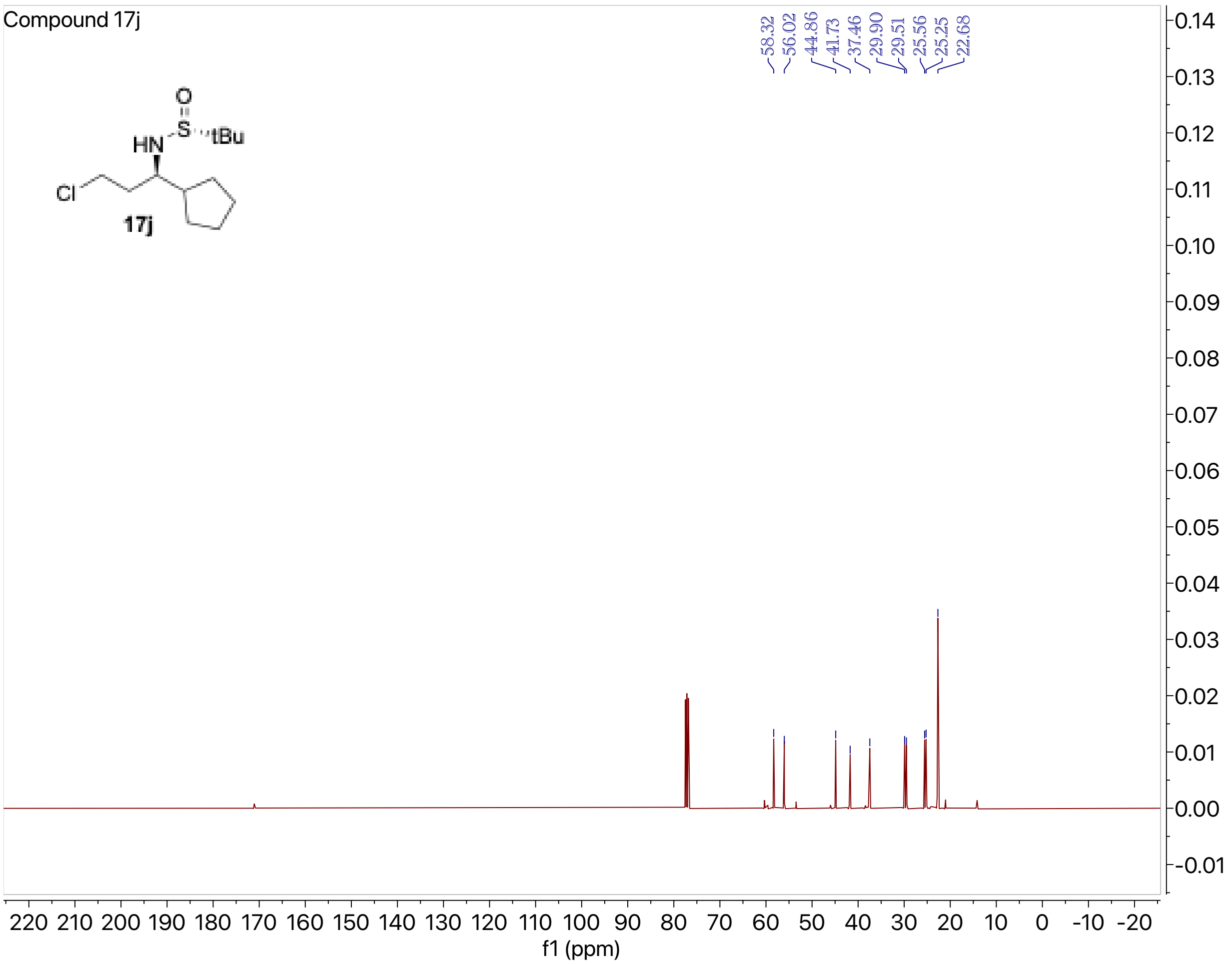

Acquired on a 400 MHz JEOL spectrometer.

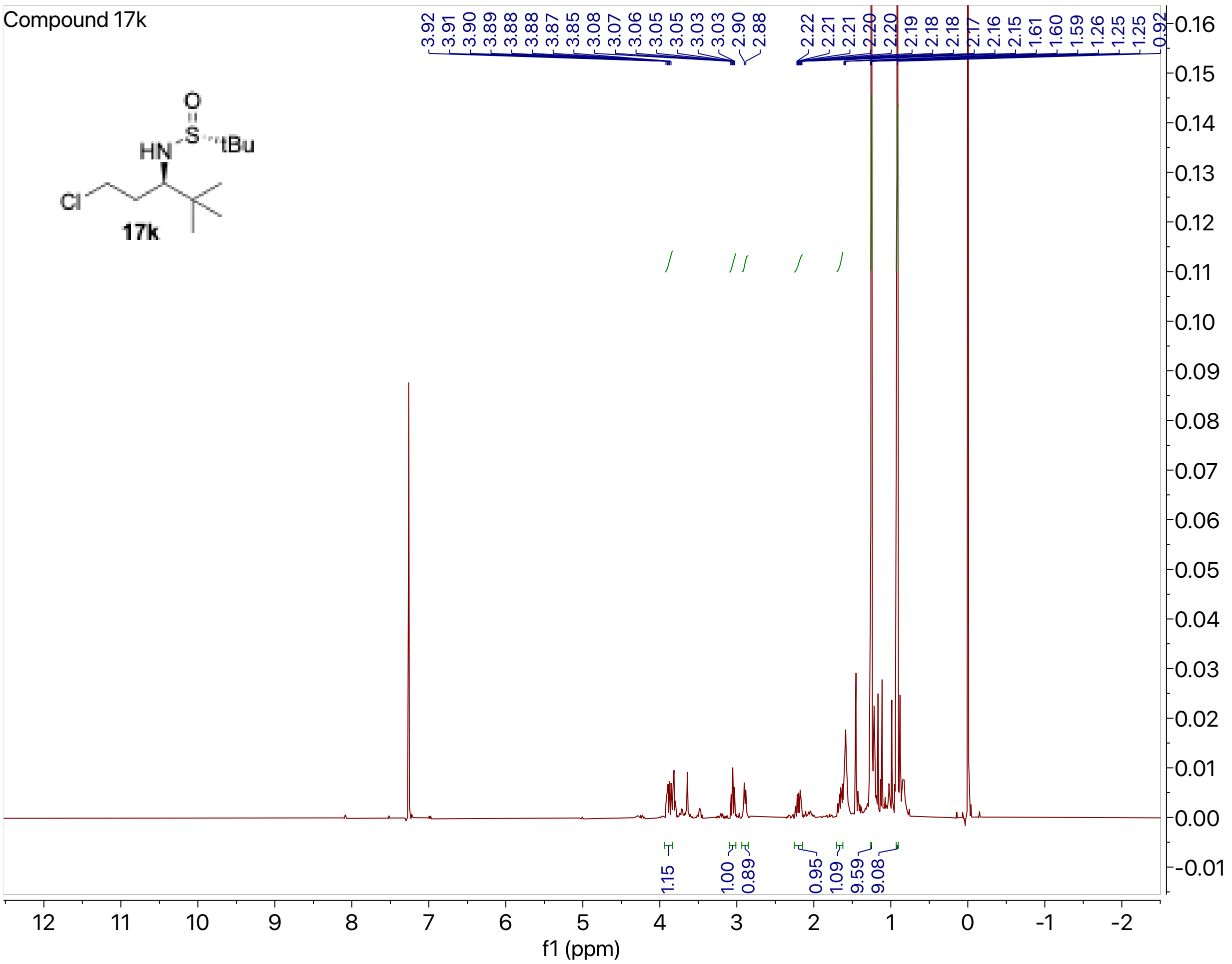

Acquired on a 400 MHz JEOL spectrometer.

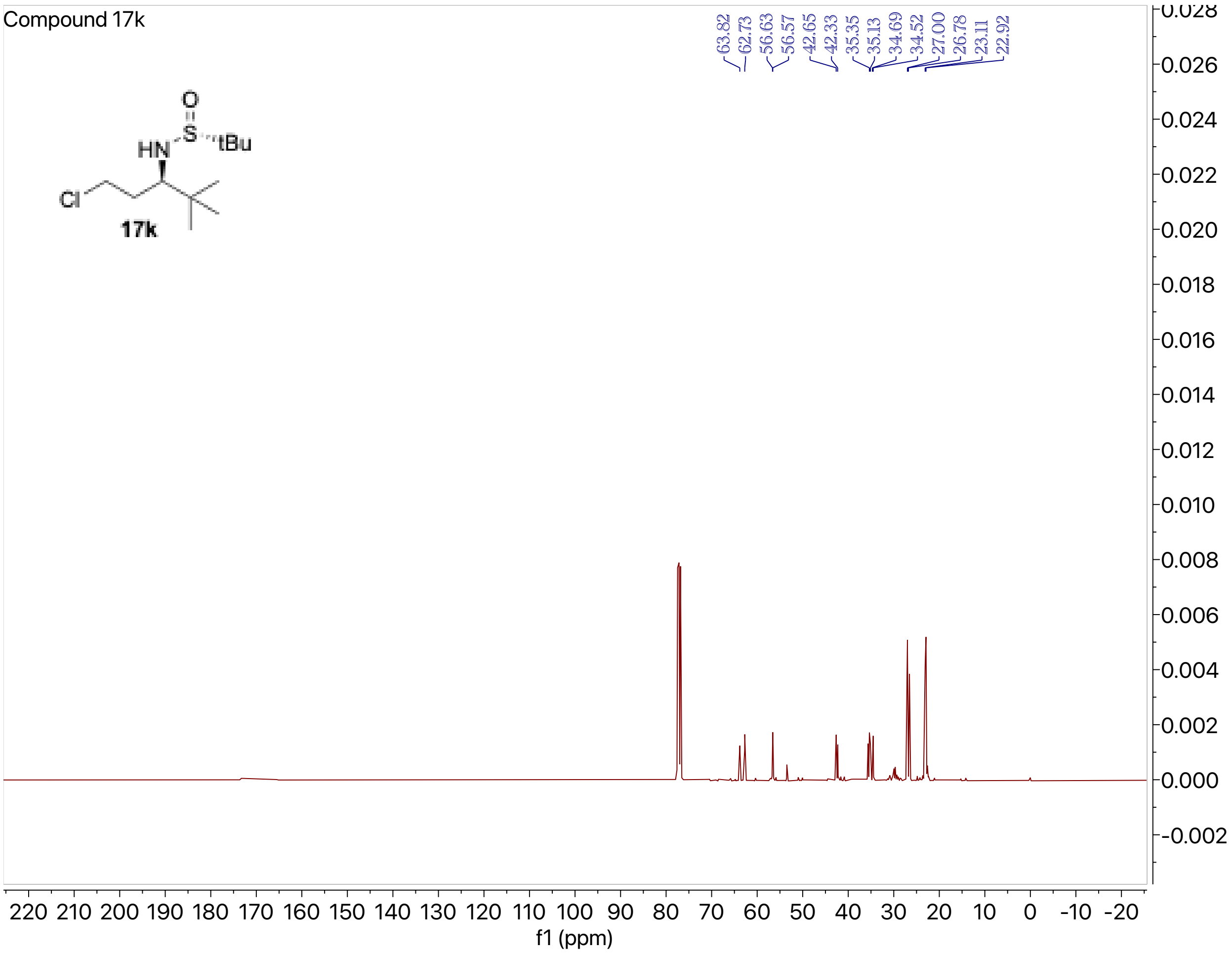

Acquired on a 400 MHz JEOL spectrometer.

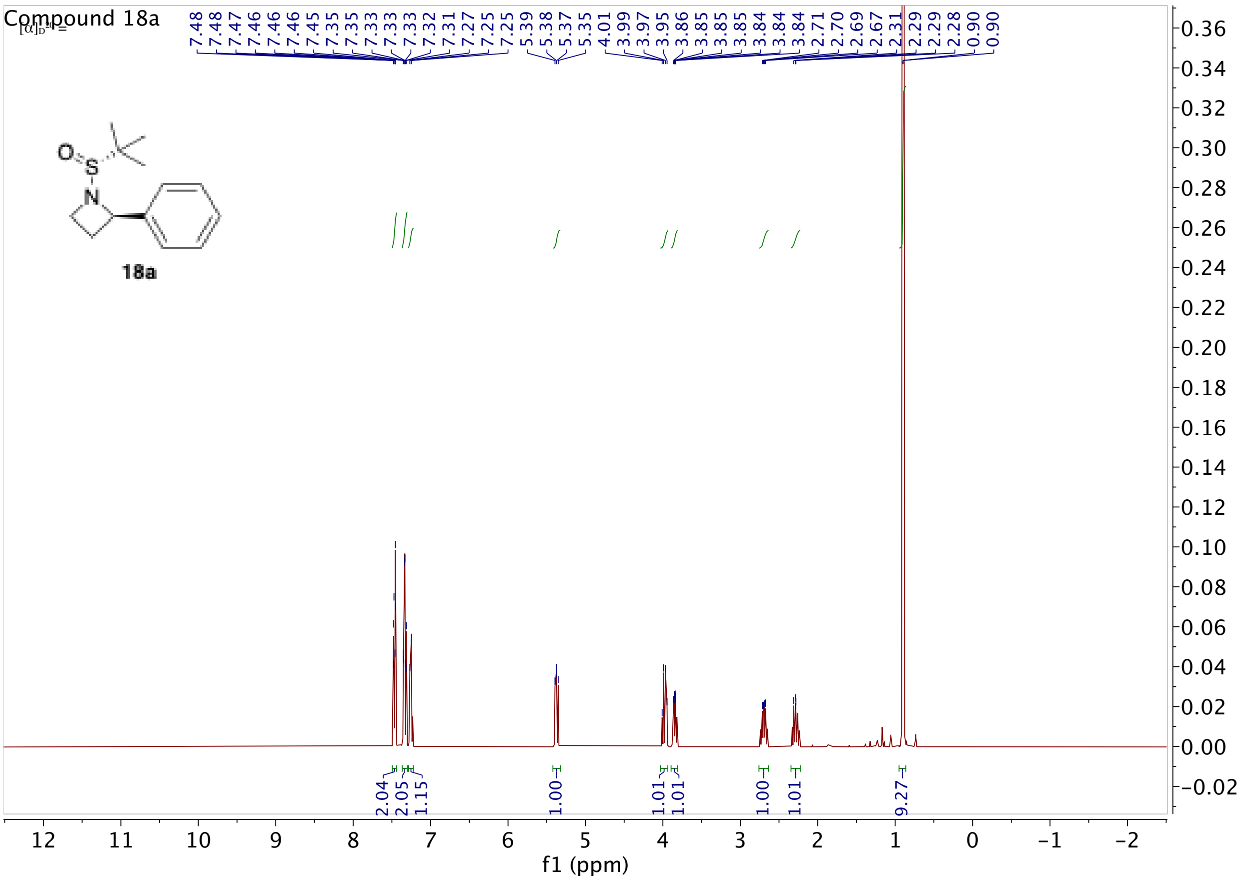

Acquired on a 500 MHz JEOL spectrometer.

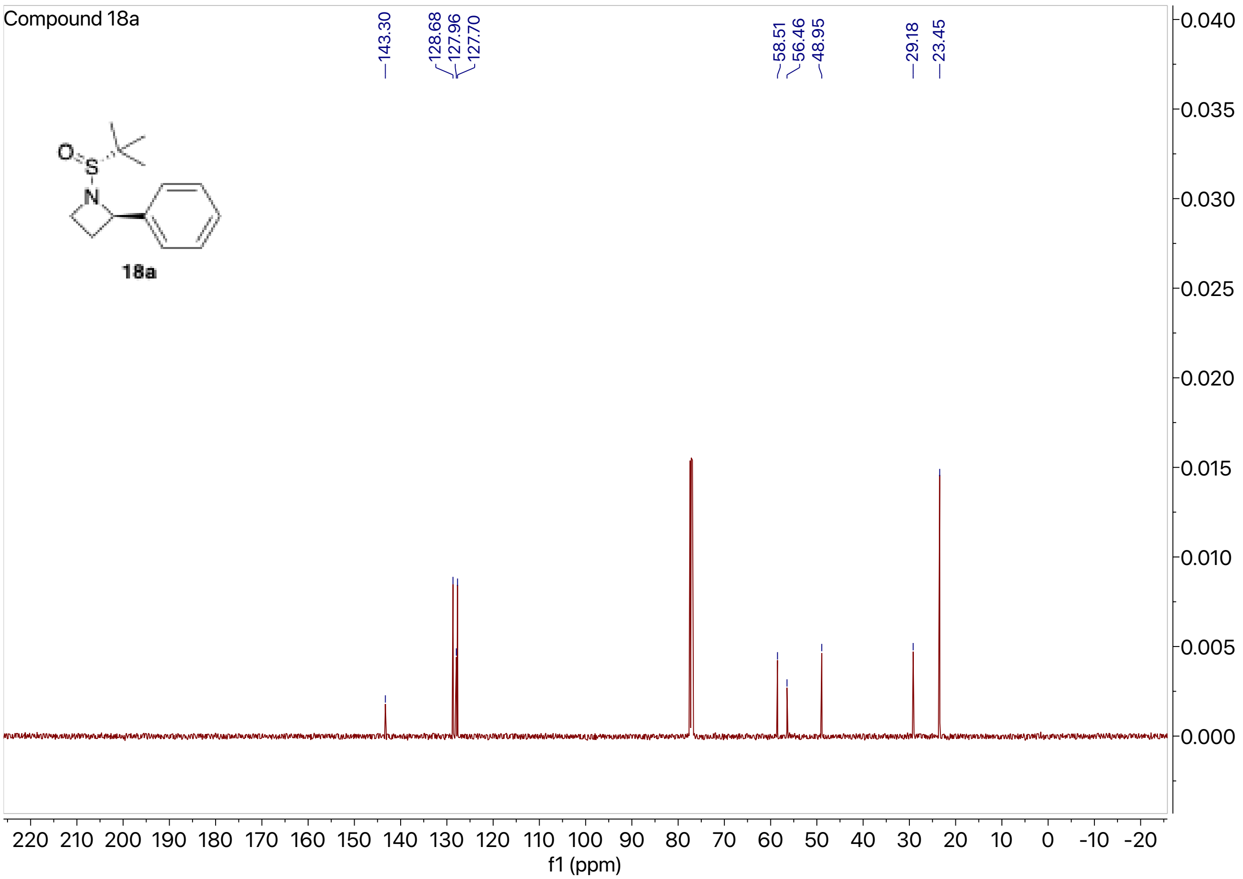

Acquired on a 400 MHz JEOL spectrometer.

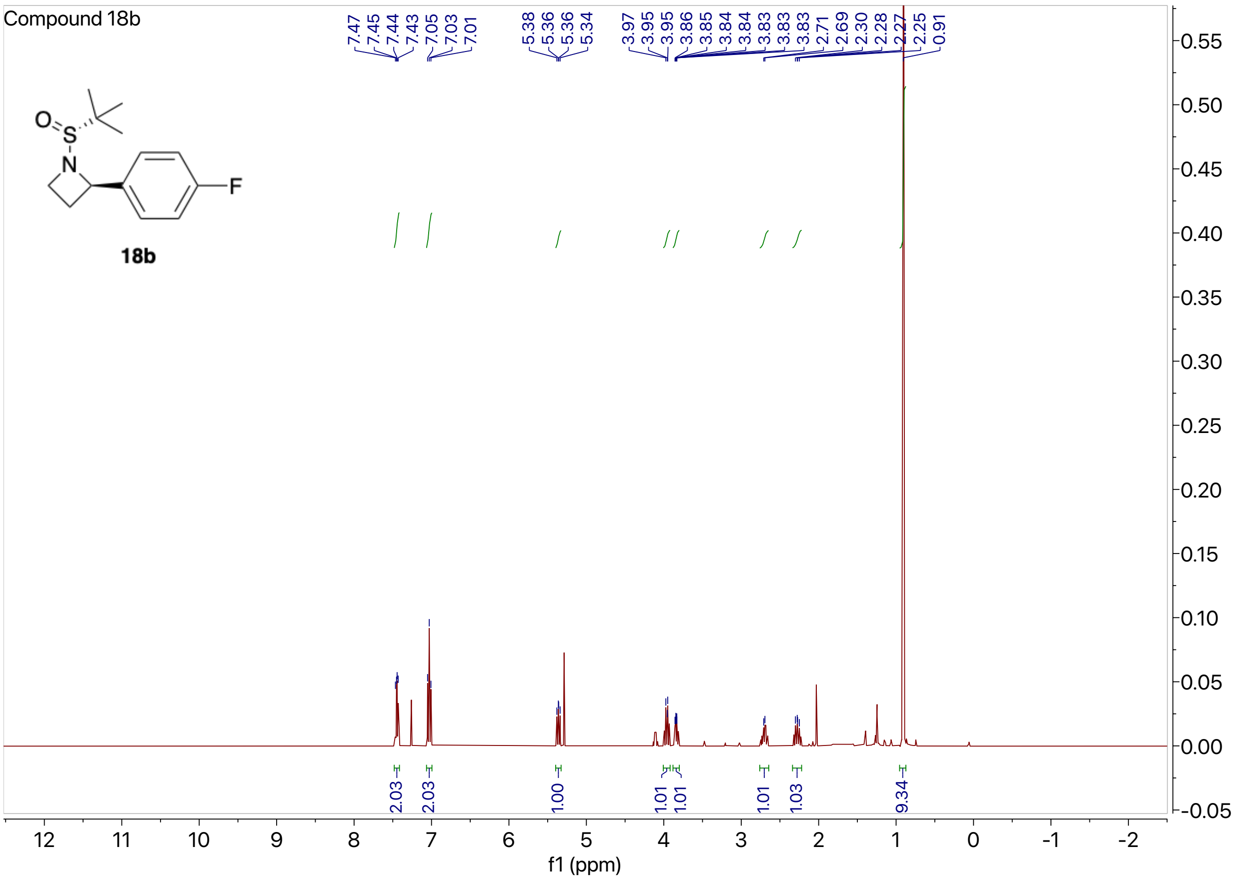

Acquired on a 400 MHz JEOL spectrometer.

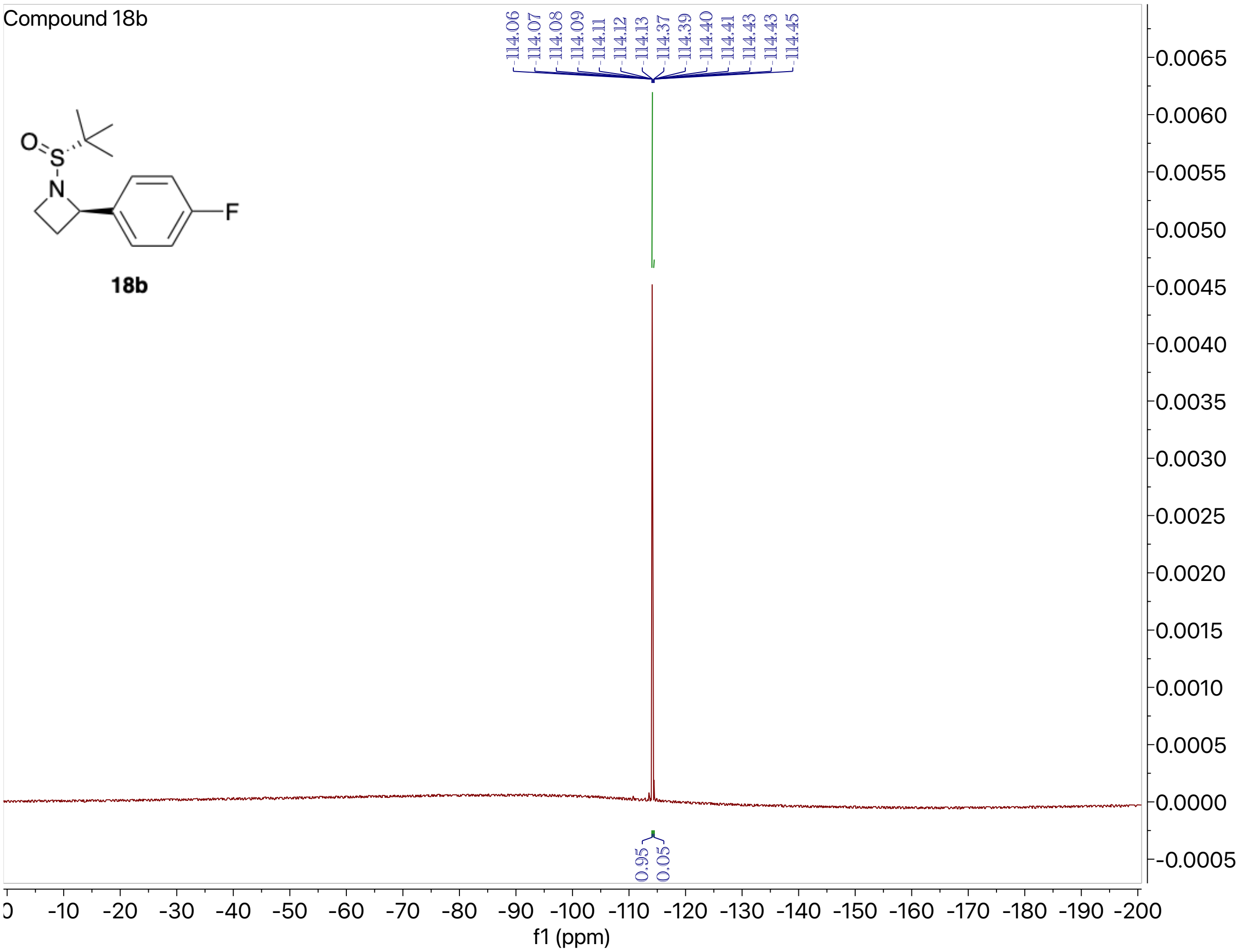

Acquired on a 400 MHz JEOL spectrometer.

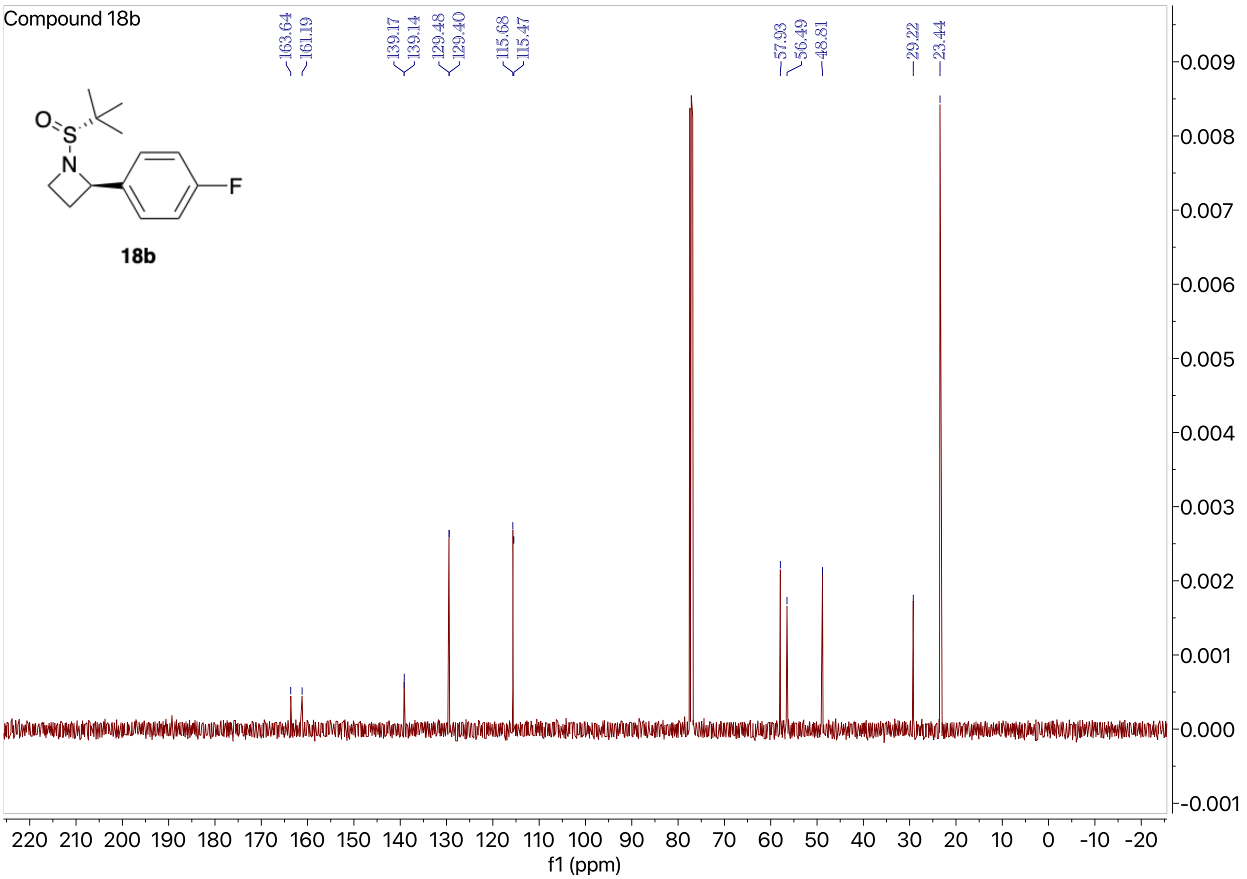

Acquired on a 400 MHz JEOL spectrometer.

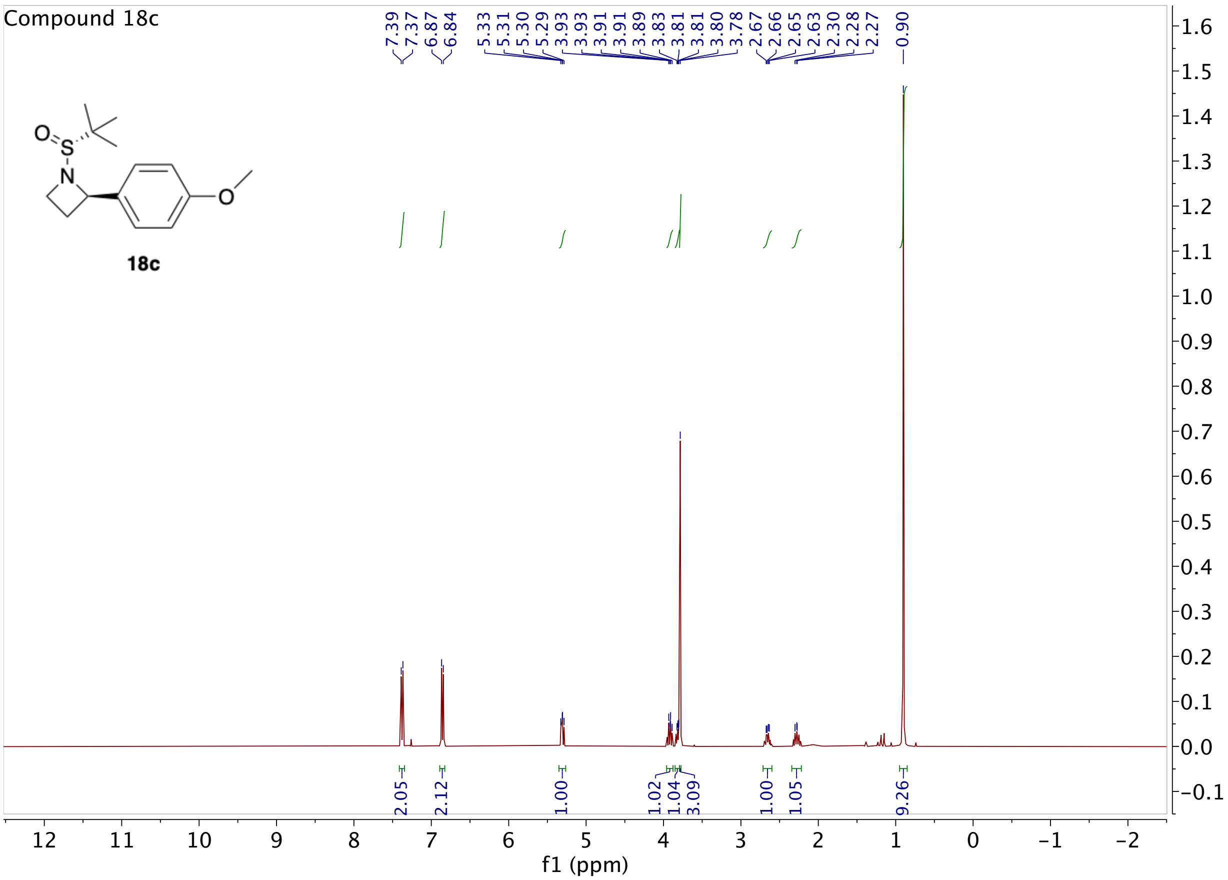

Acquired on a 400 MHz JEOL spectrometer.

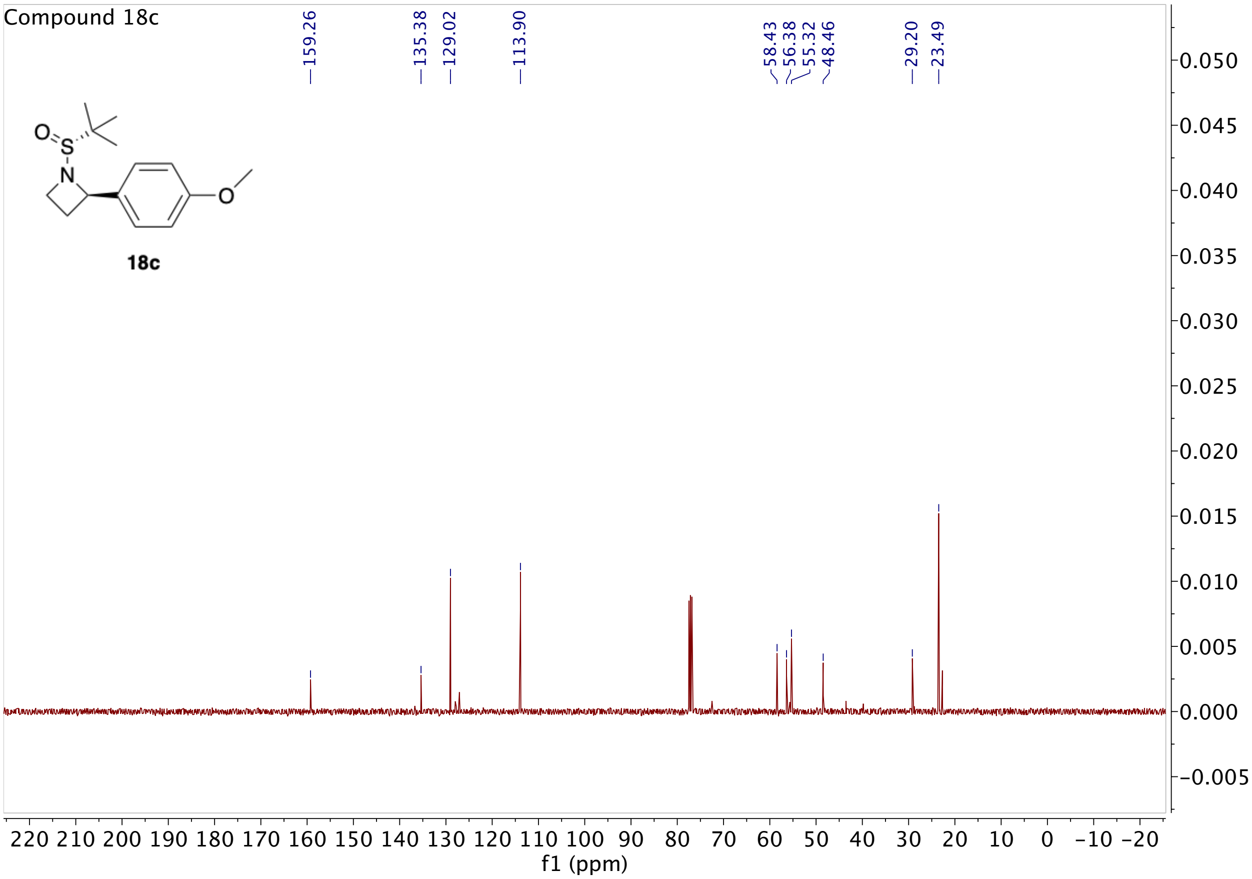

Acquired on a 500 MHz JEOL spectrometer.

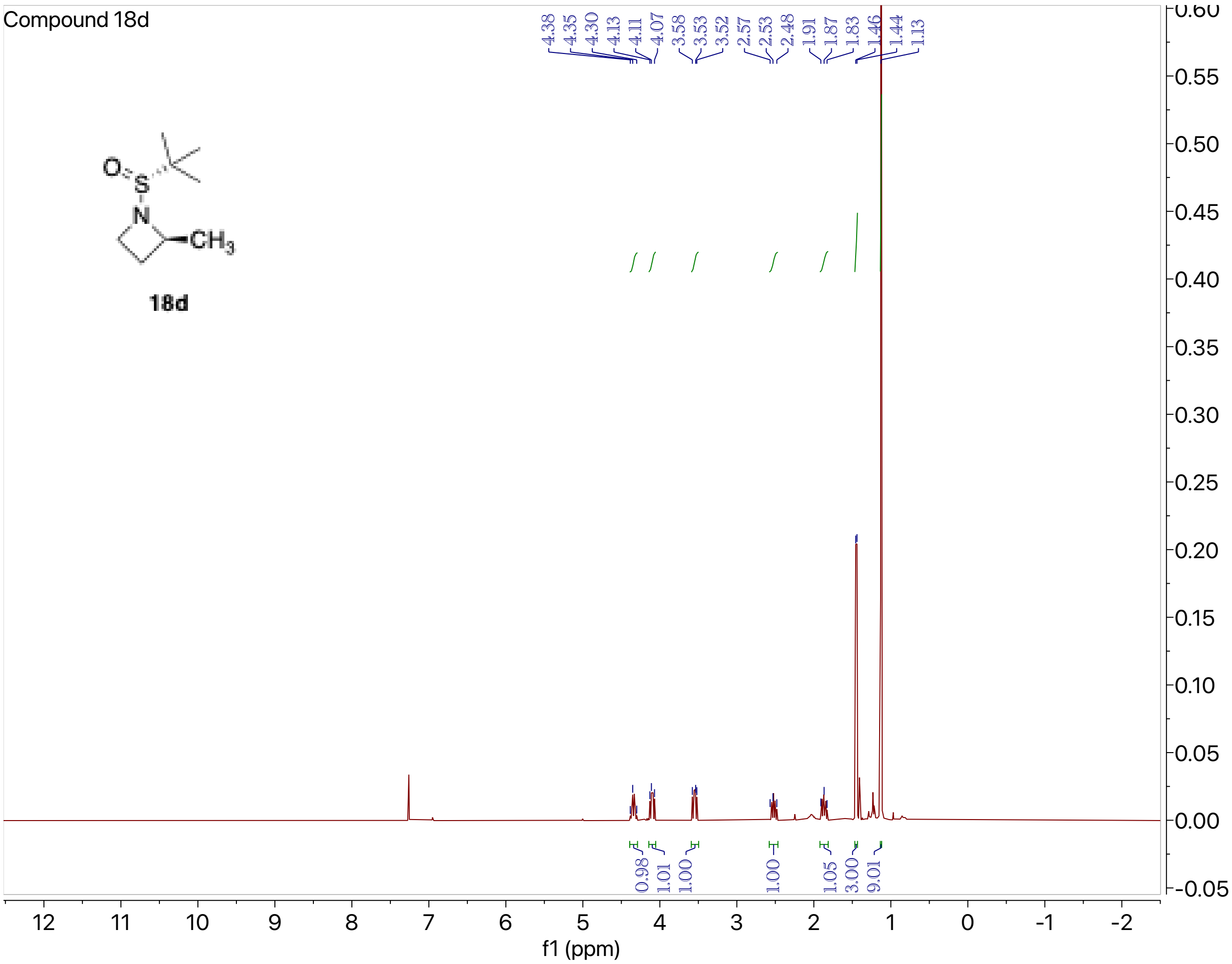

Acquired on a 400 MHz JEOL spectrometer.

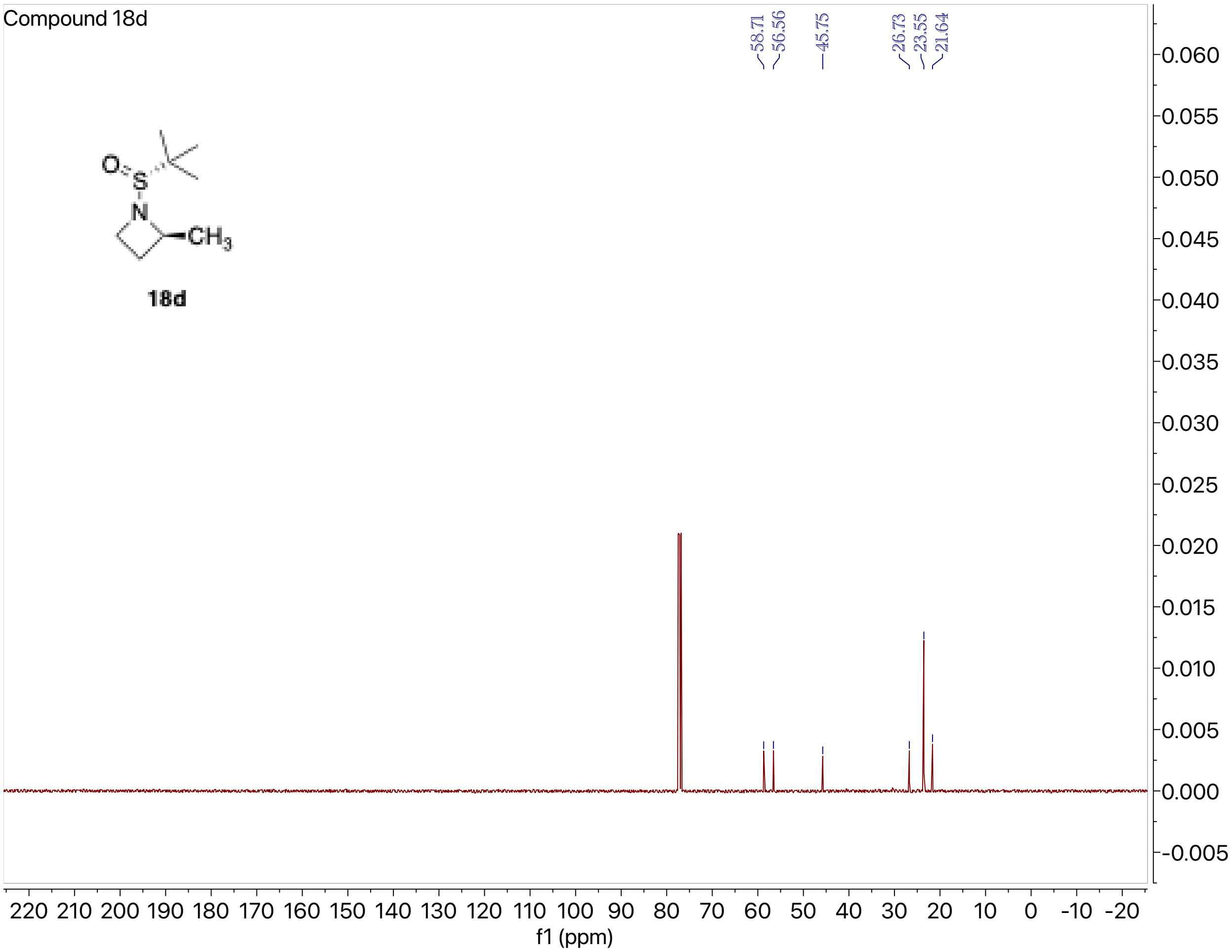

Acquired on a 400 MHz JEOL spectrometer.

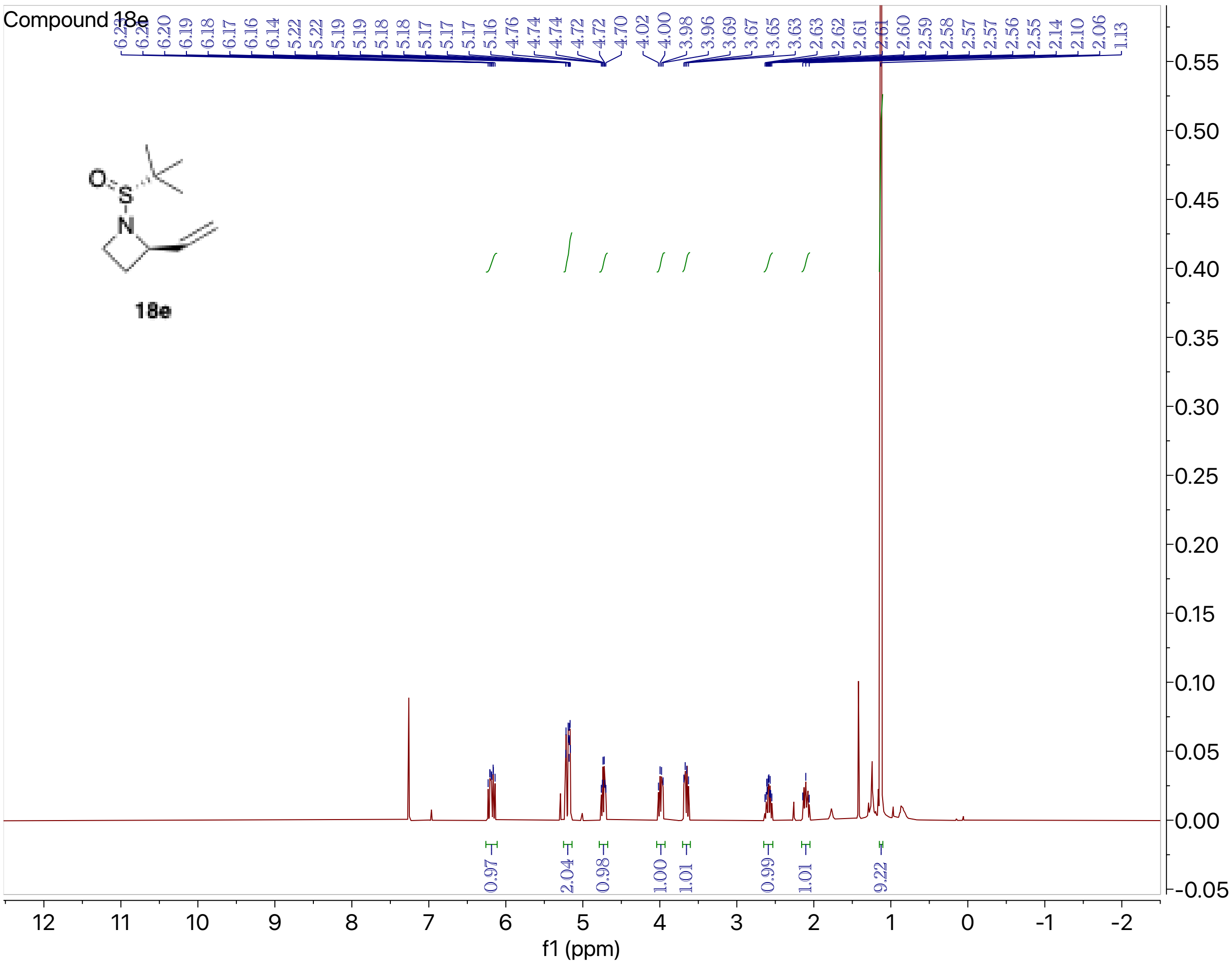

Acquired on a 400 MHz JEOL spectrometer.

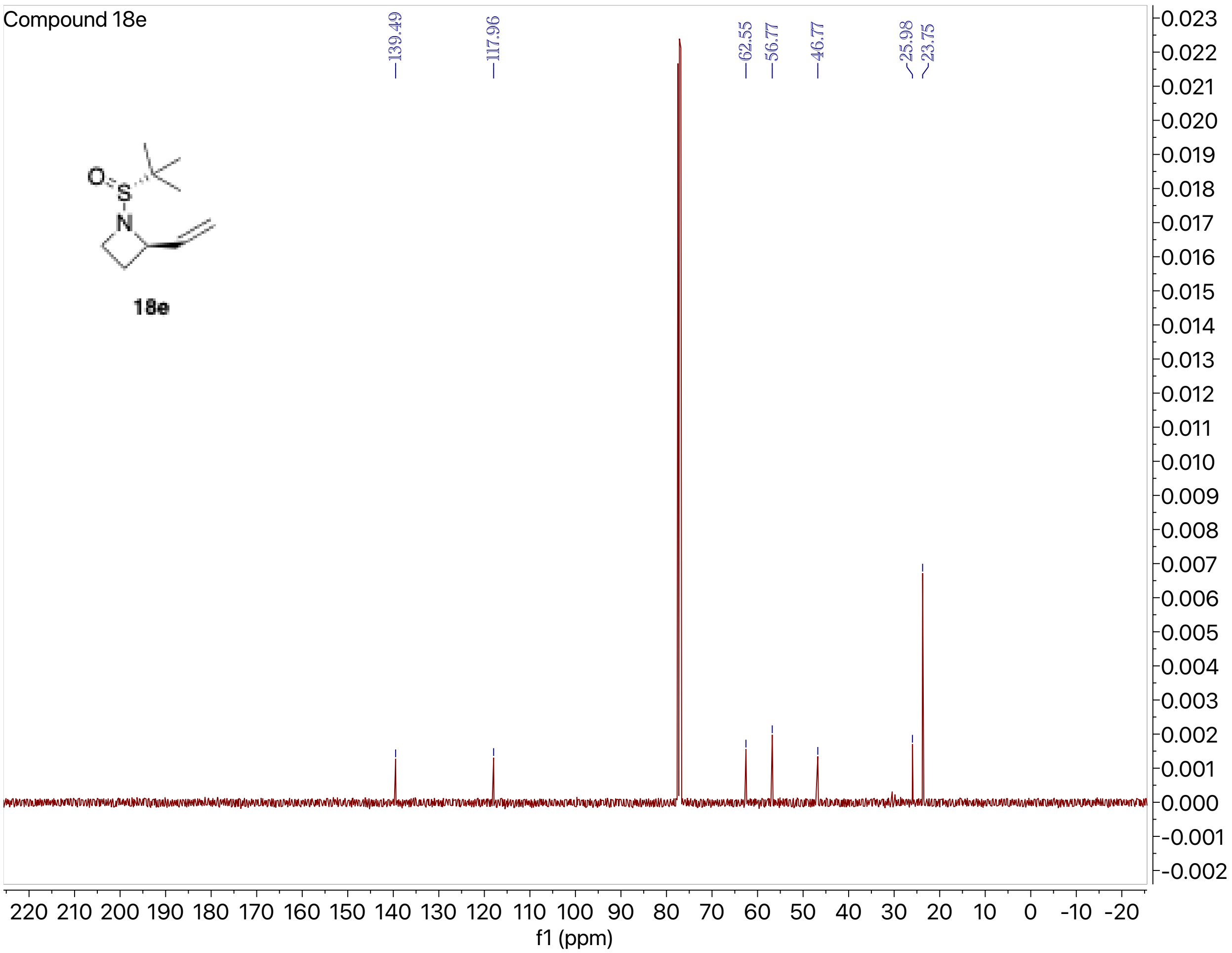

Acquired on a 400 MHz JEOL spectrometer.

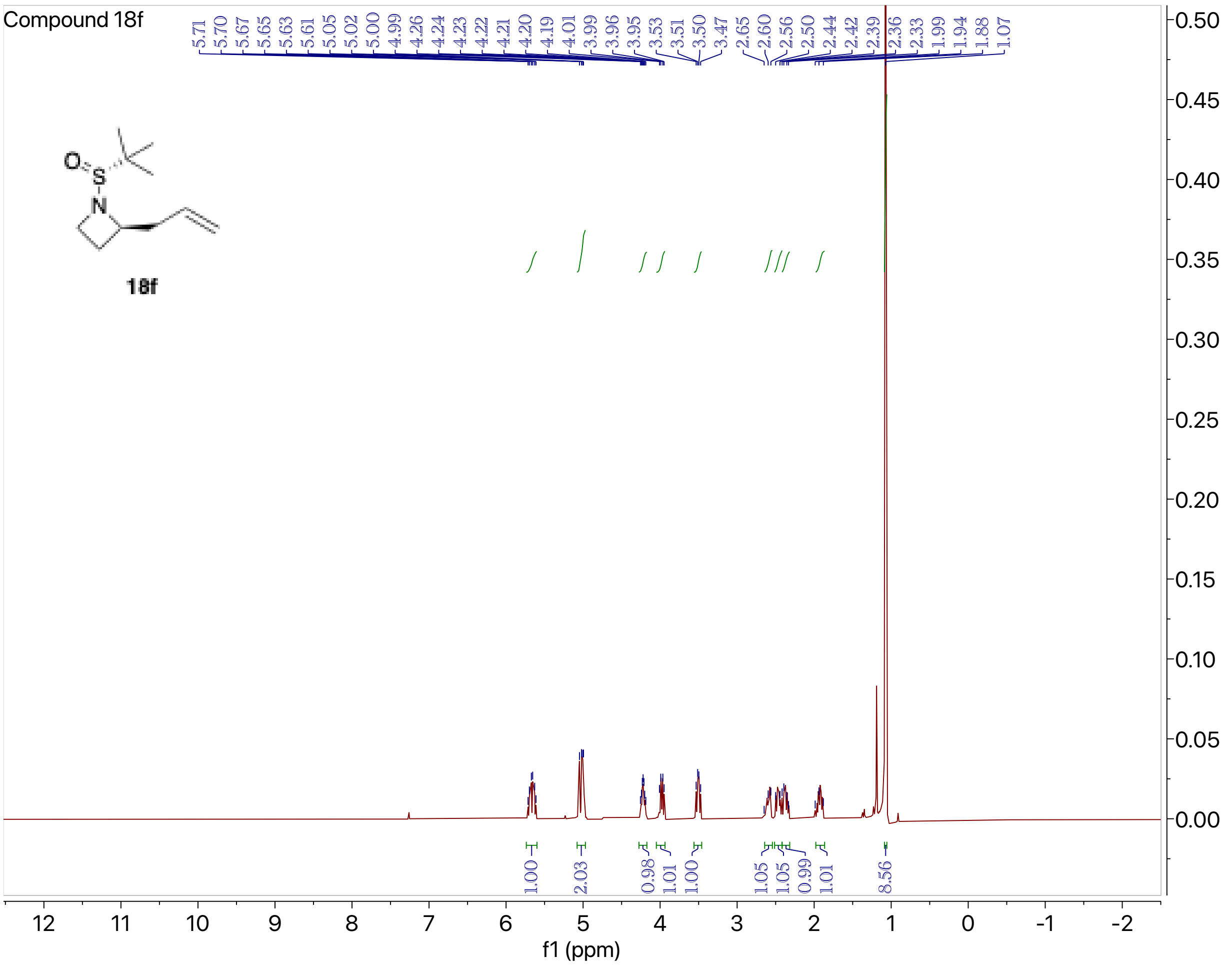

Acquired on a 400 MHz JEOL spectrometer.

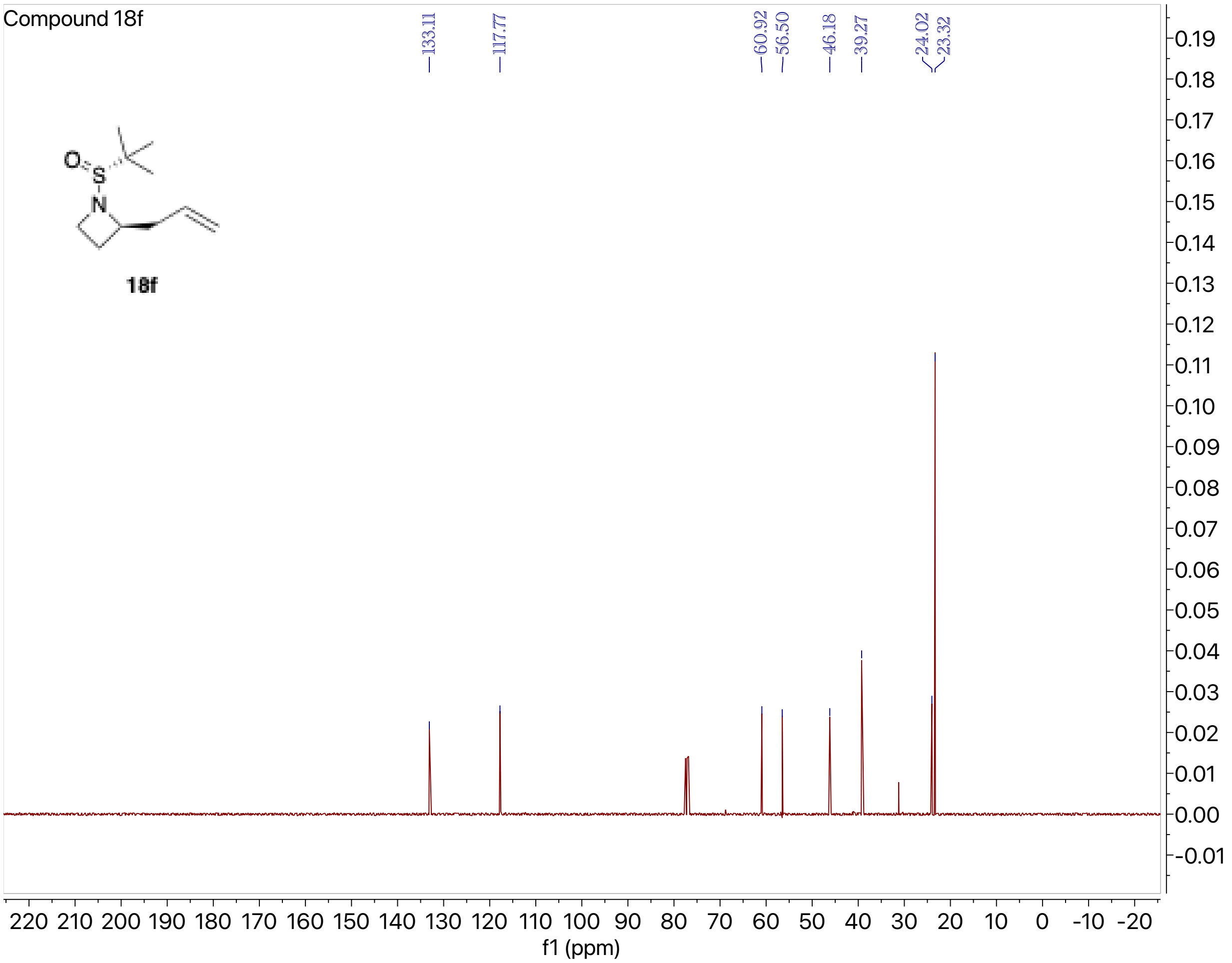

Acquired on a 400 MHz JEOL spectrometer.

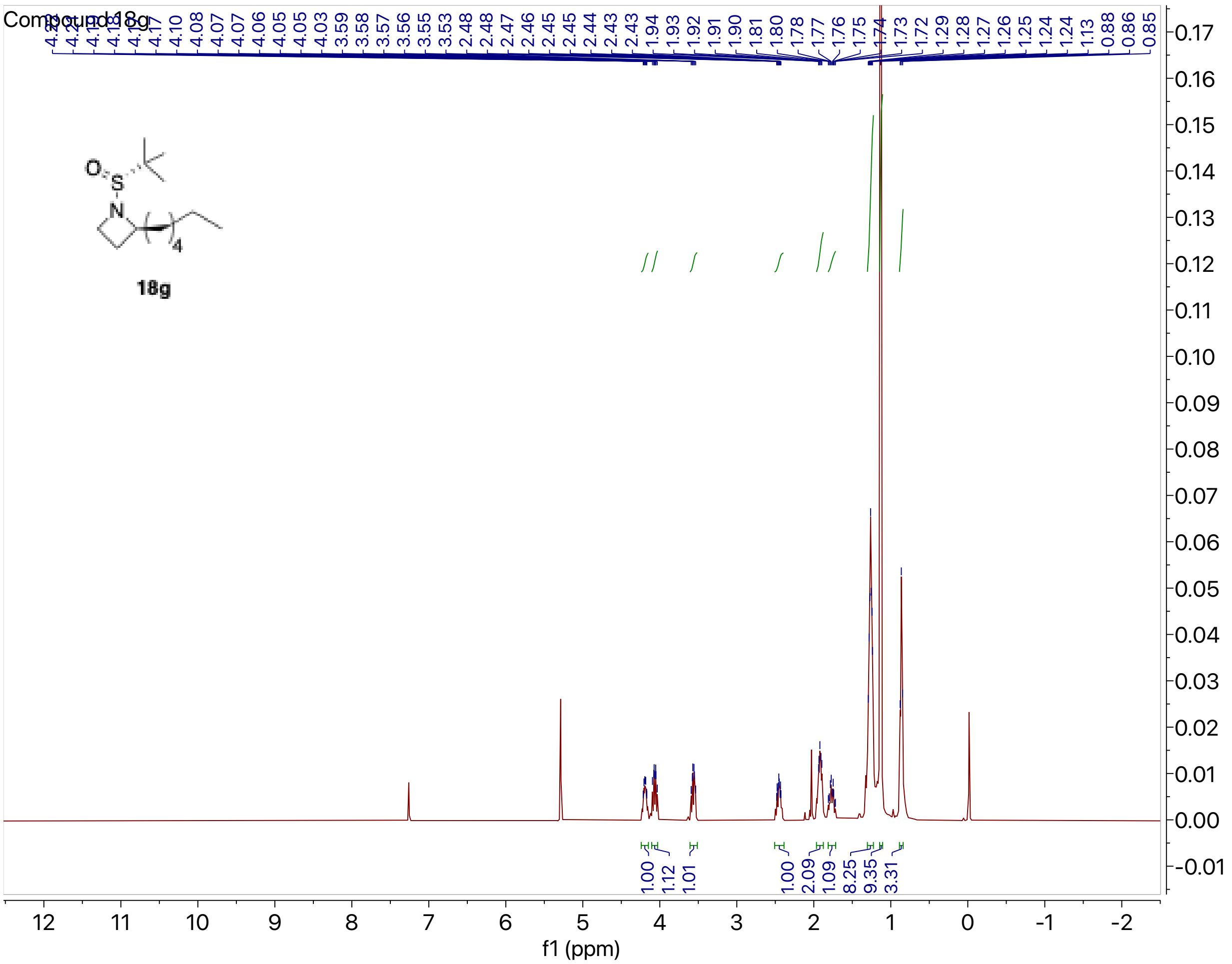

Acquired on a 400 MHz JEOL spectrometer.

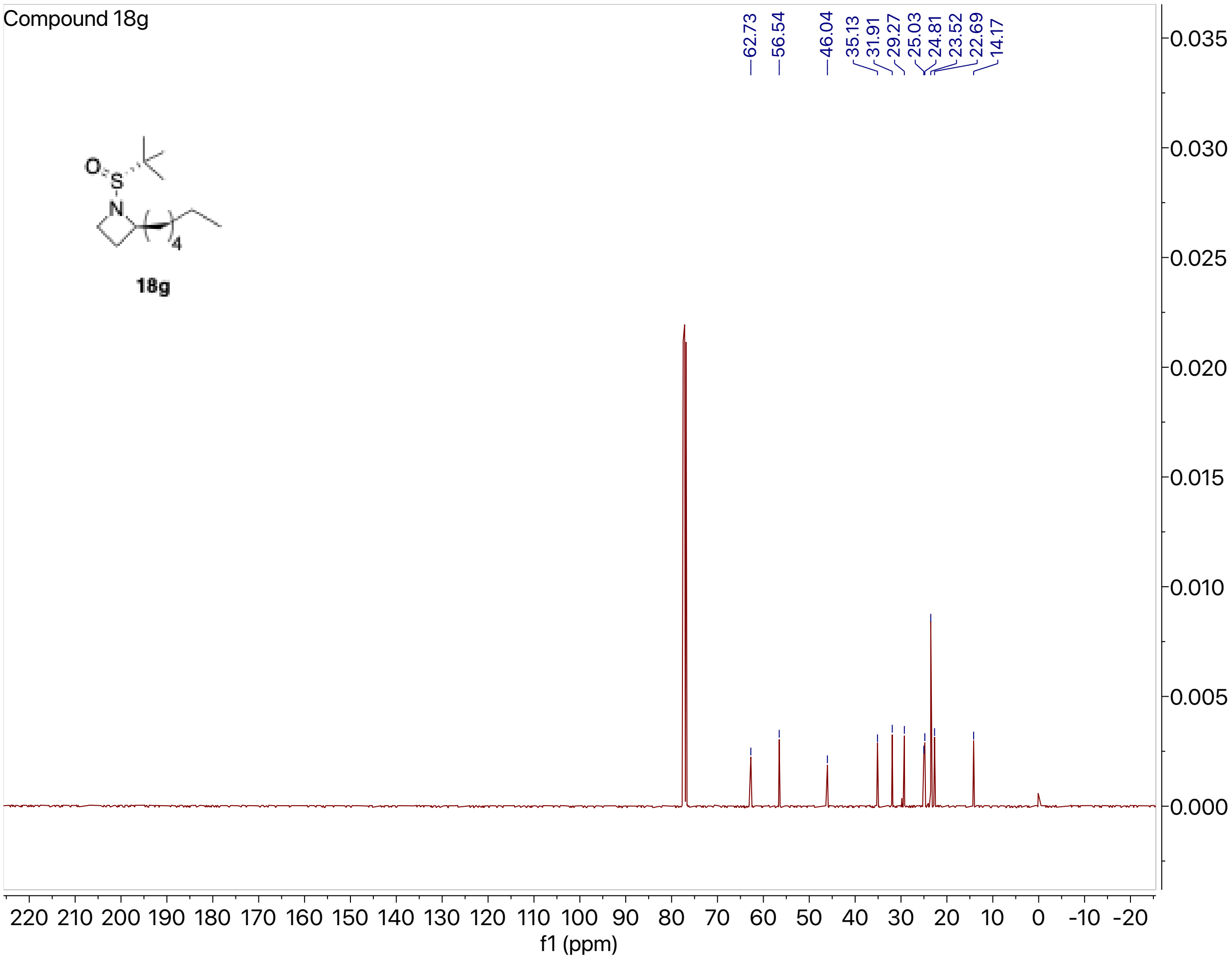

Acquired on a 400 MHz JEOL spectrometer.

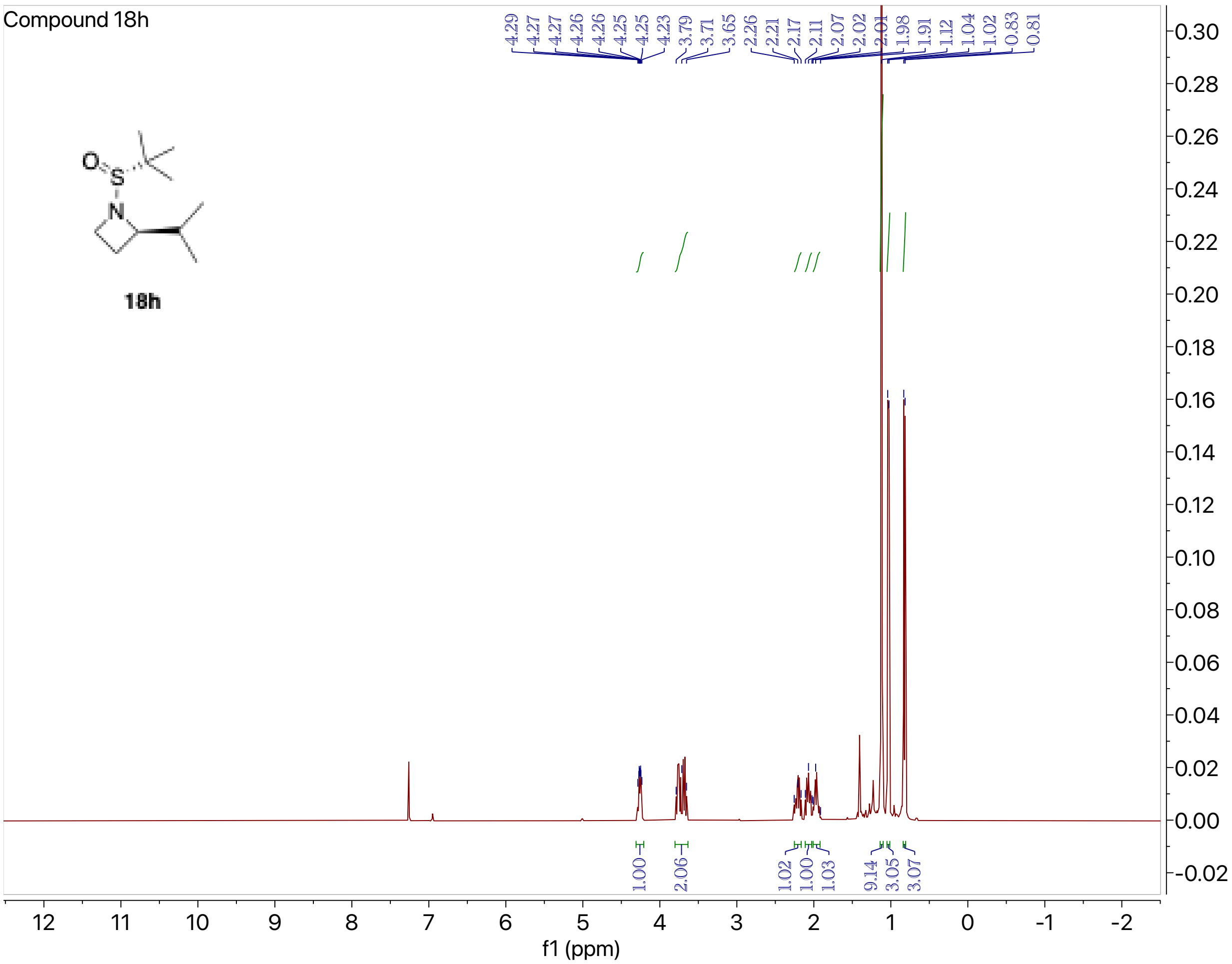

Acquired on a 400 MHz JEOL spectrometer.

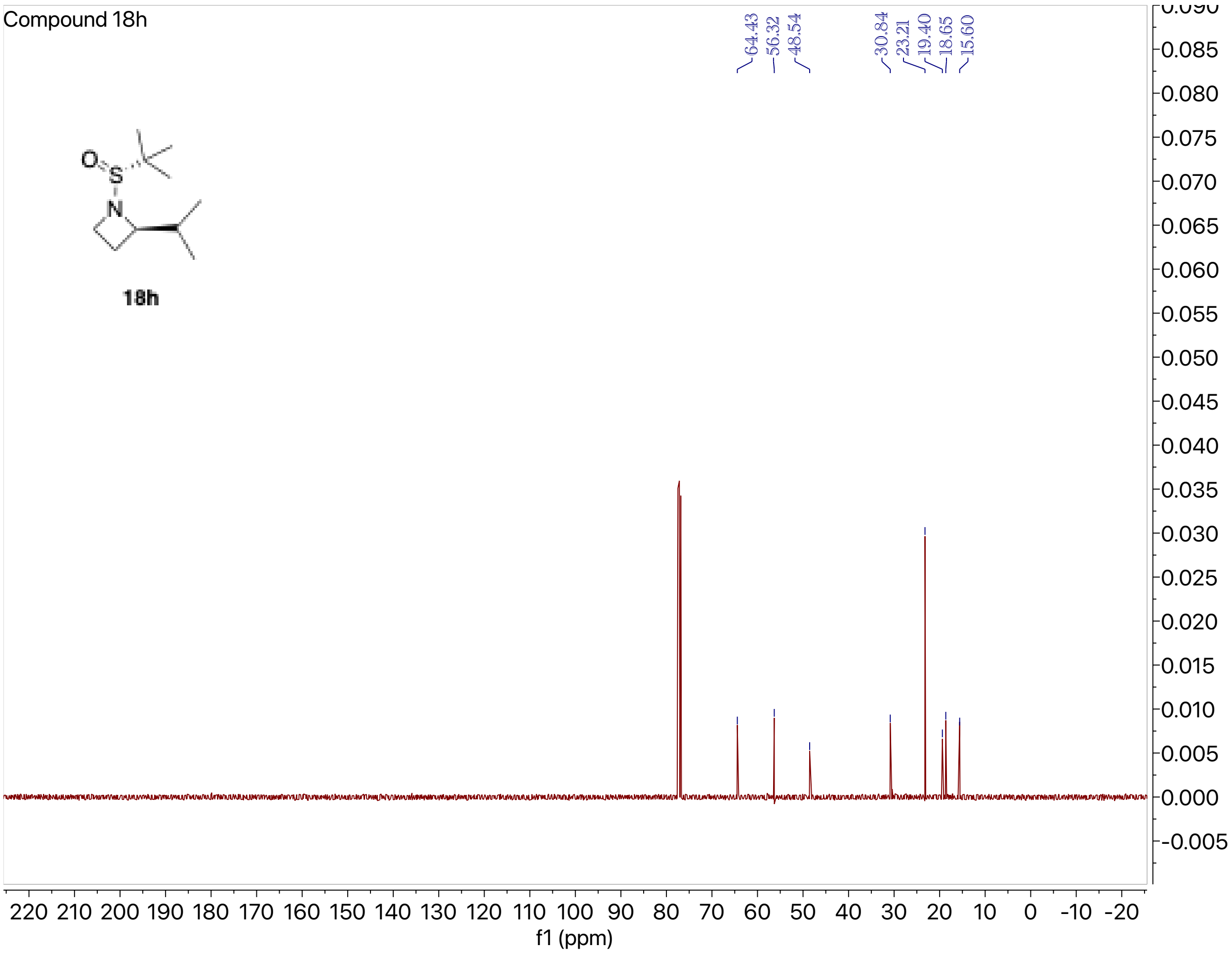

Acquired on a 400 MHz JEOL spectrometer.

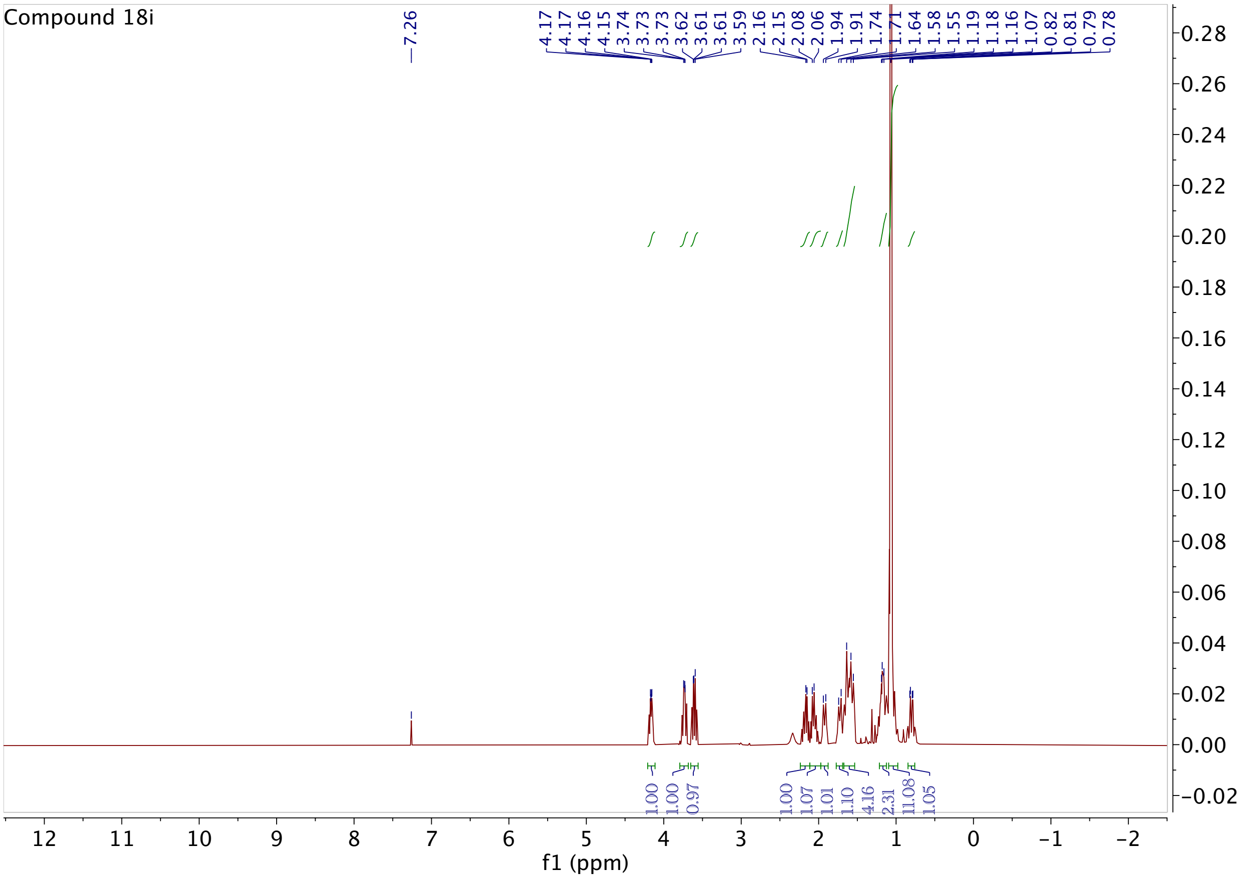

Acquired on a 400 MHz JEOL spectrometer.

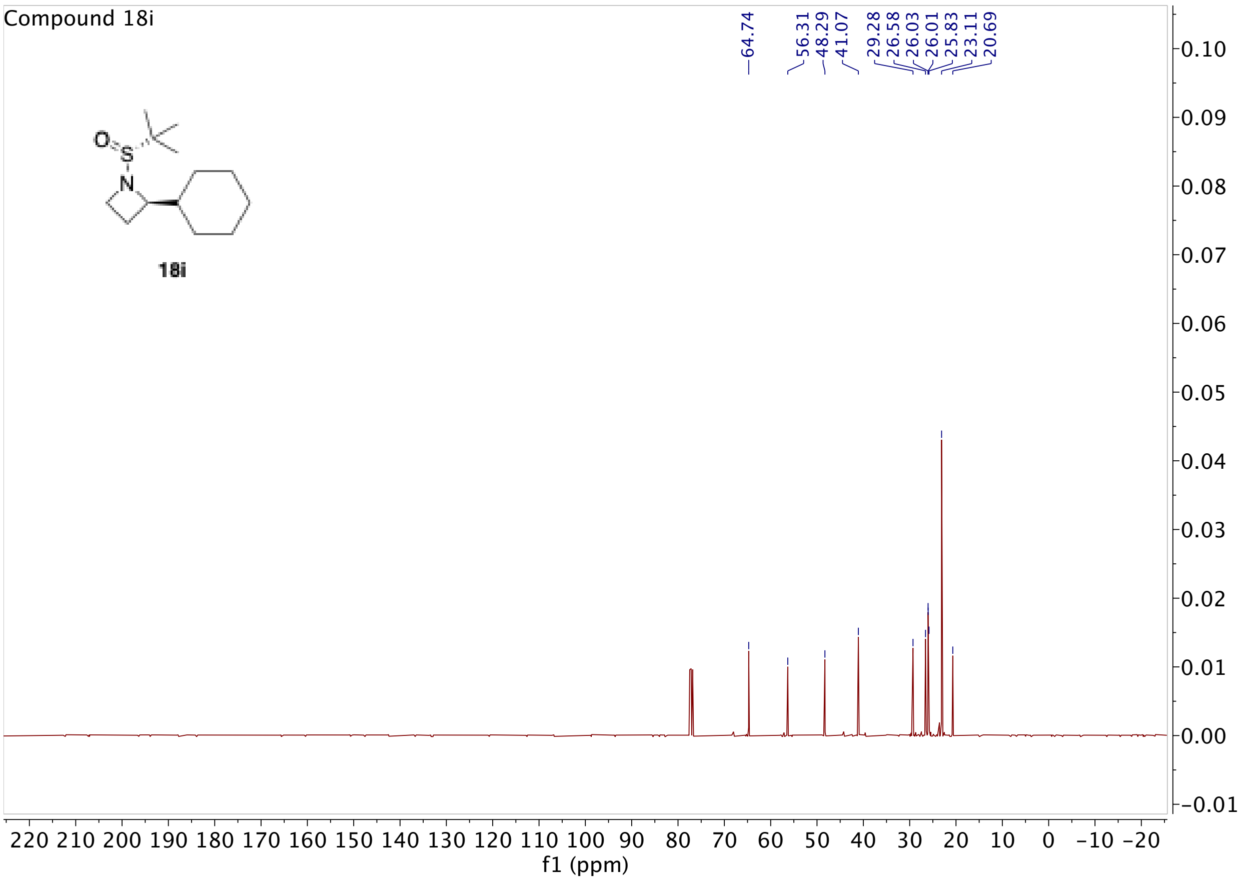

Acquired on a 400 MHz JEOL spectrometer.

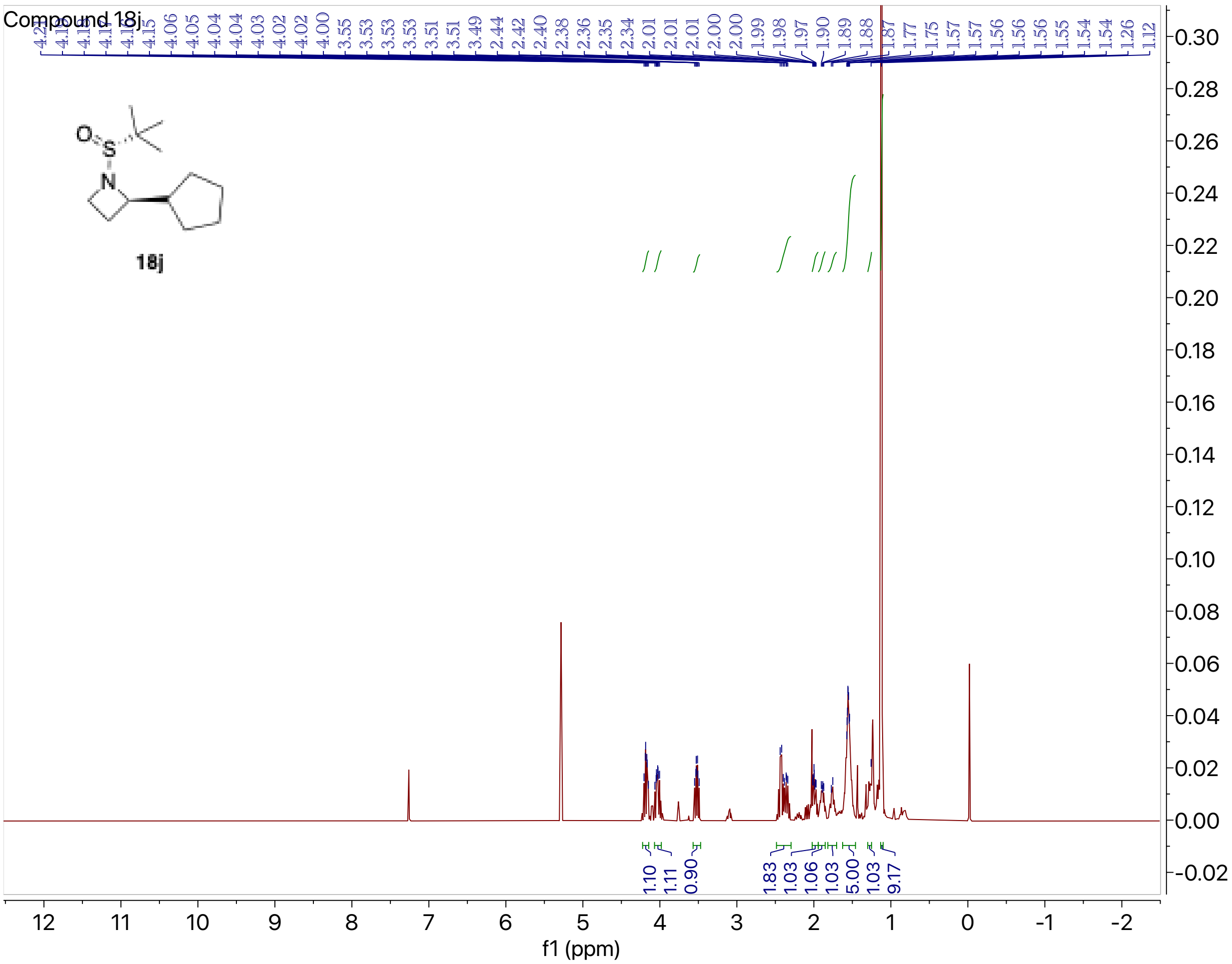

Acquired on a 400 MHz JEOL spectrometer.

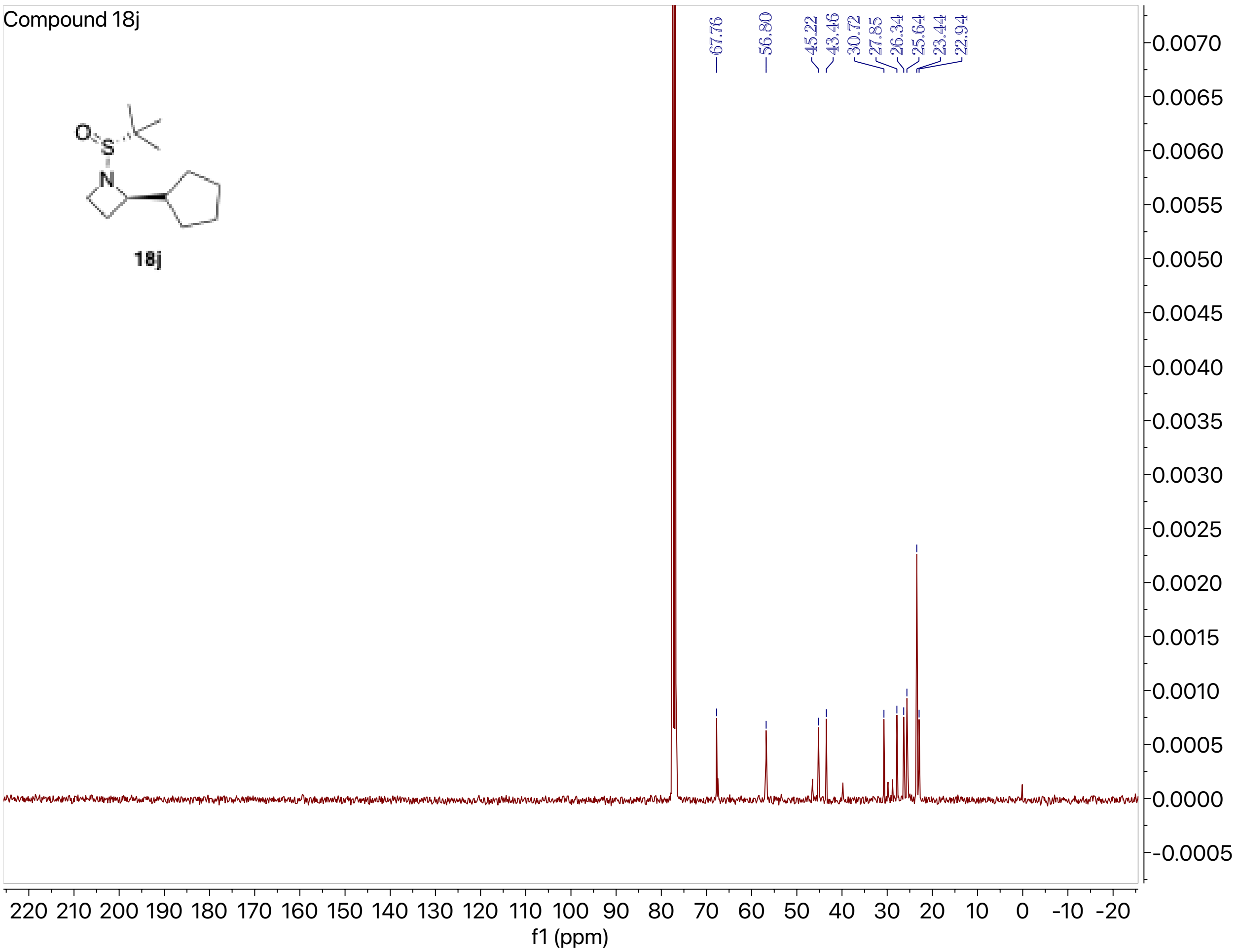

Acquired on a 400 MHz JEOL spectrometer.

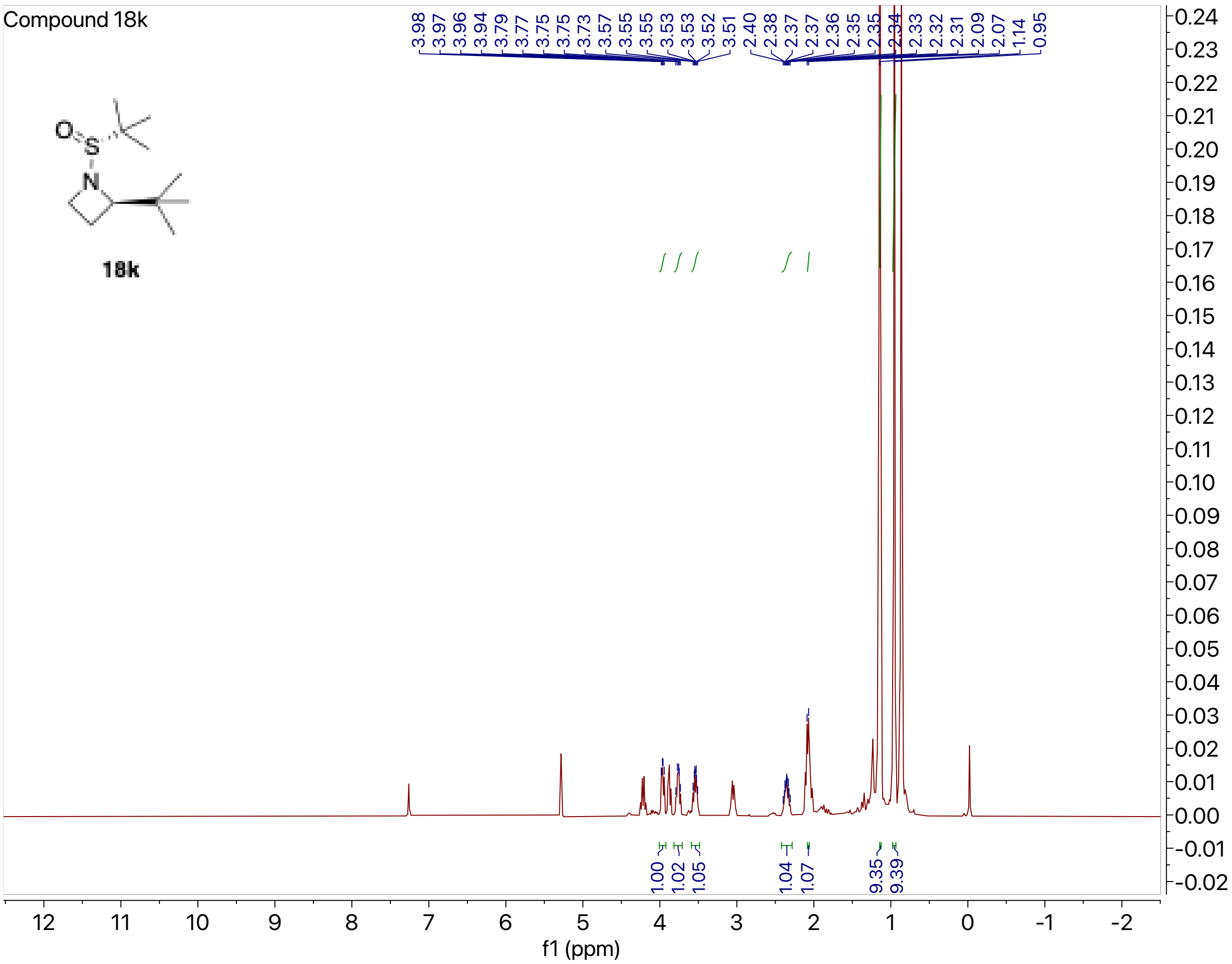

Acquired on a 400 MHz JEOL spectrometer.

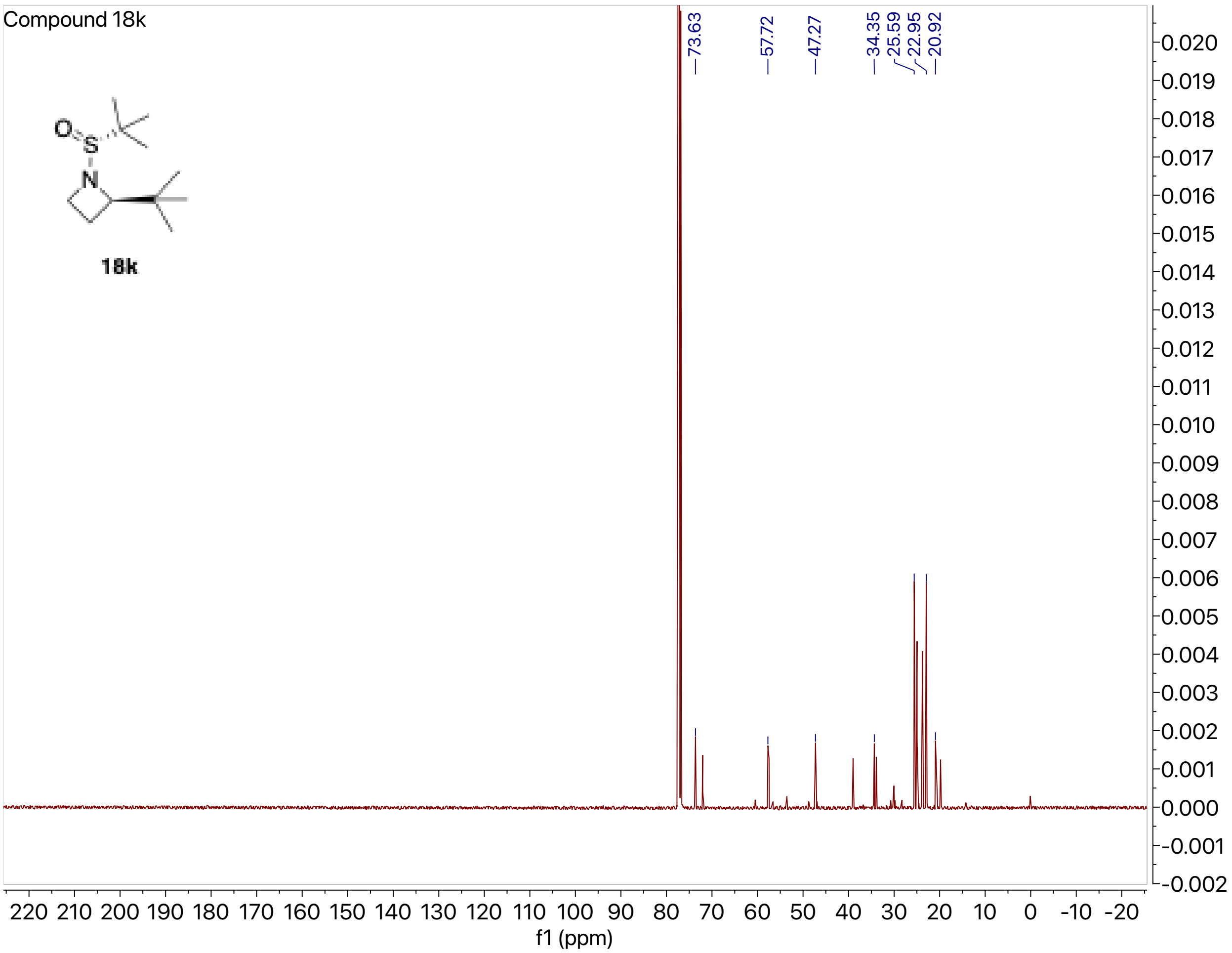

Acquired on a 400 MHz JEOL spectrometer.

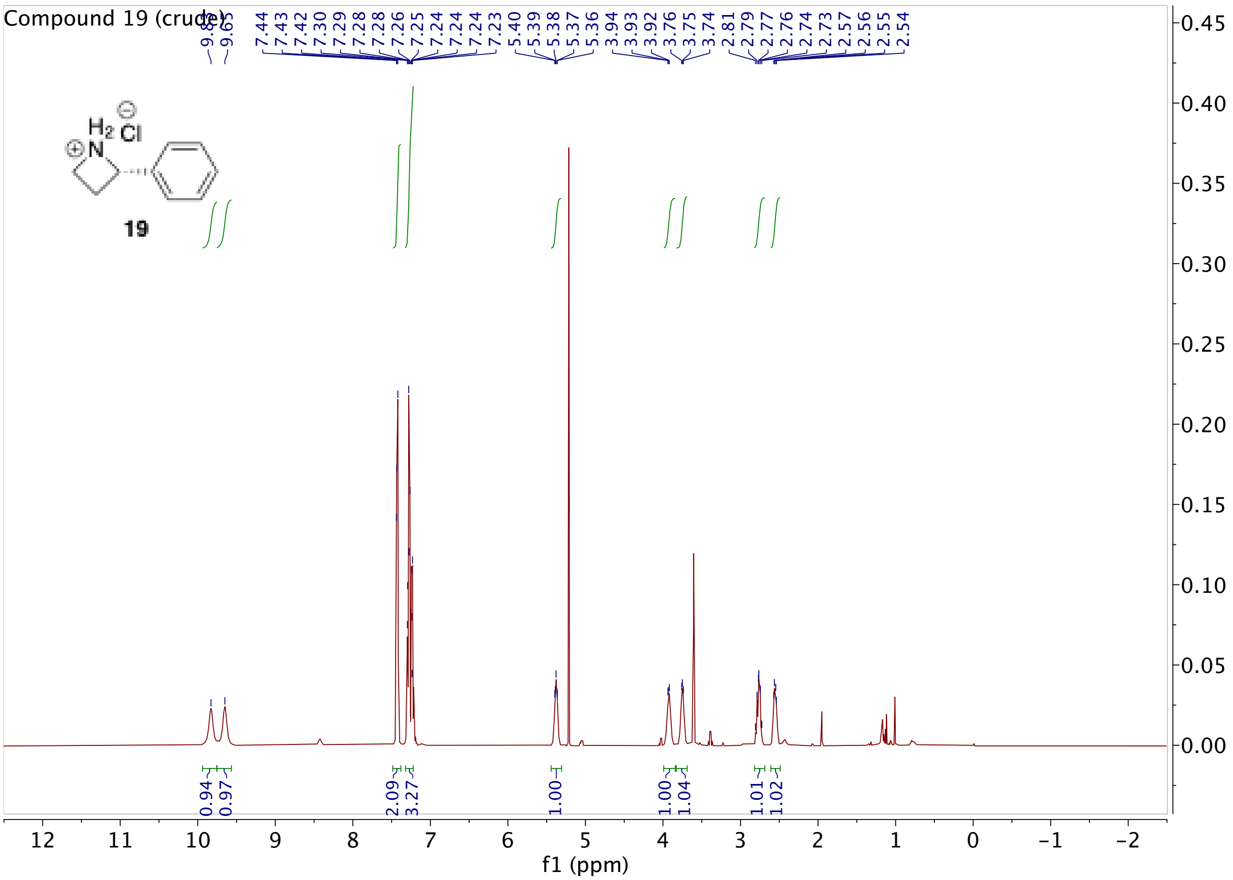

Acquired on a 400 MHz JEOL spectrometer.

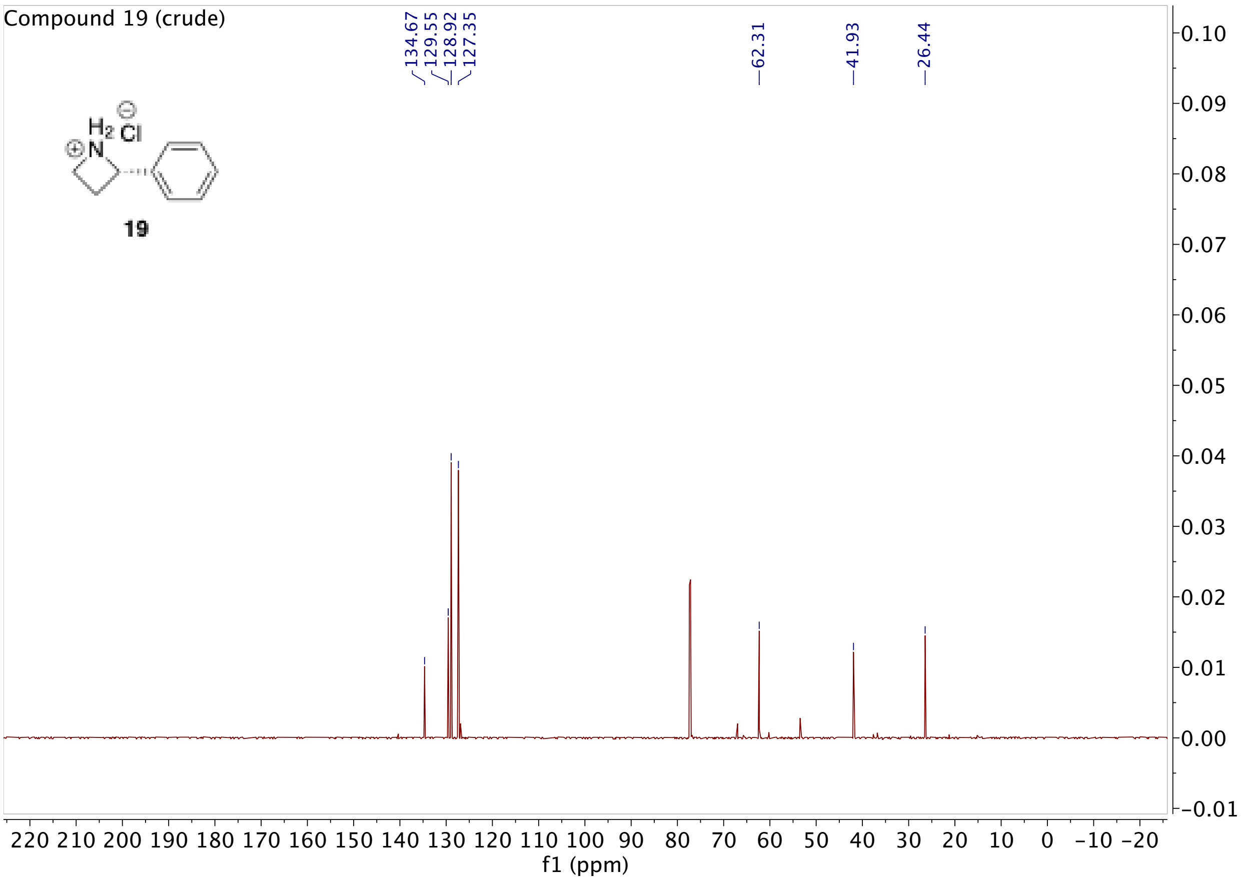

Acquired on a 400 MHz JEOL spectrometer.

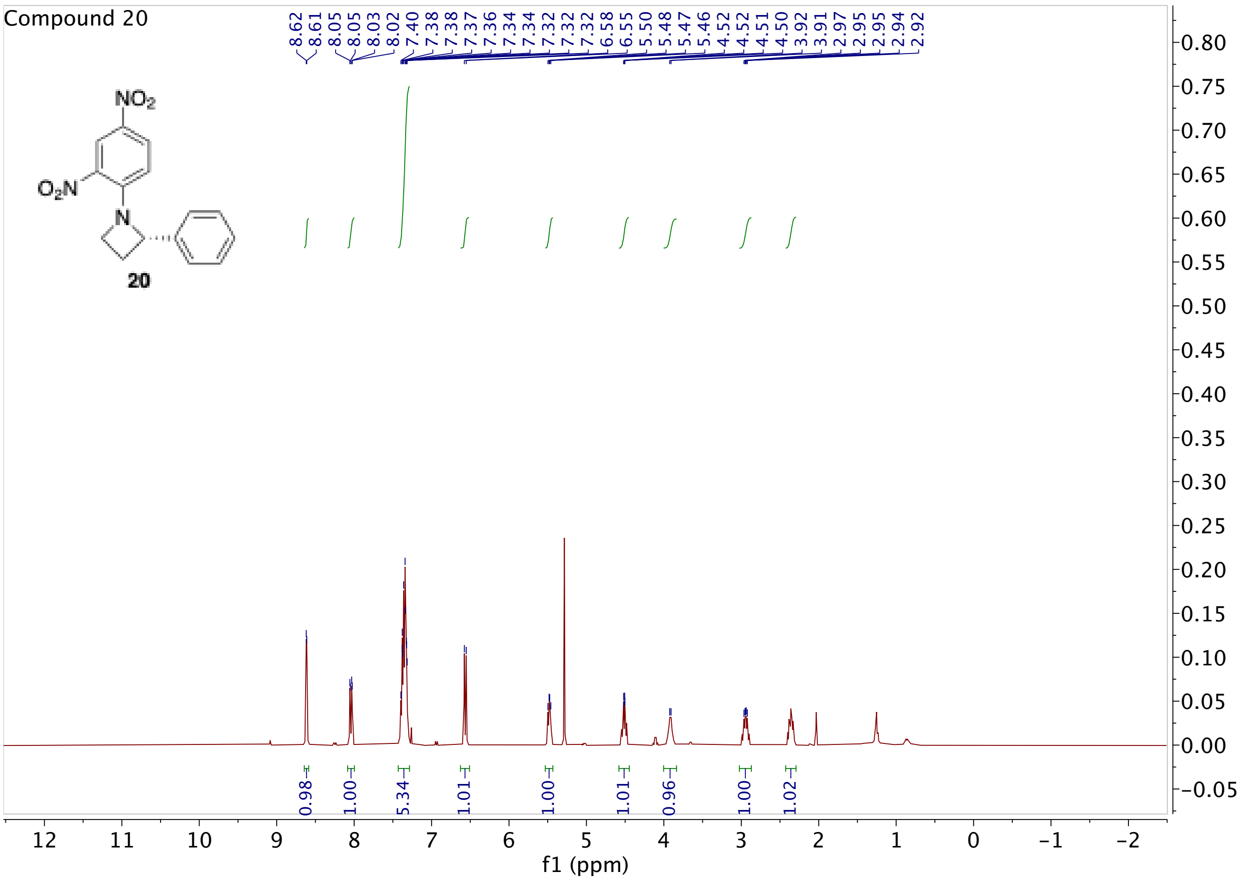

Acquired on a 400 MHz JEOL spectrometer.

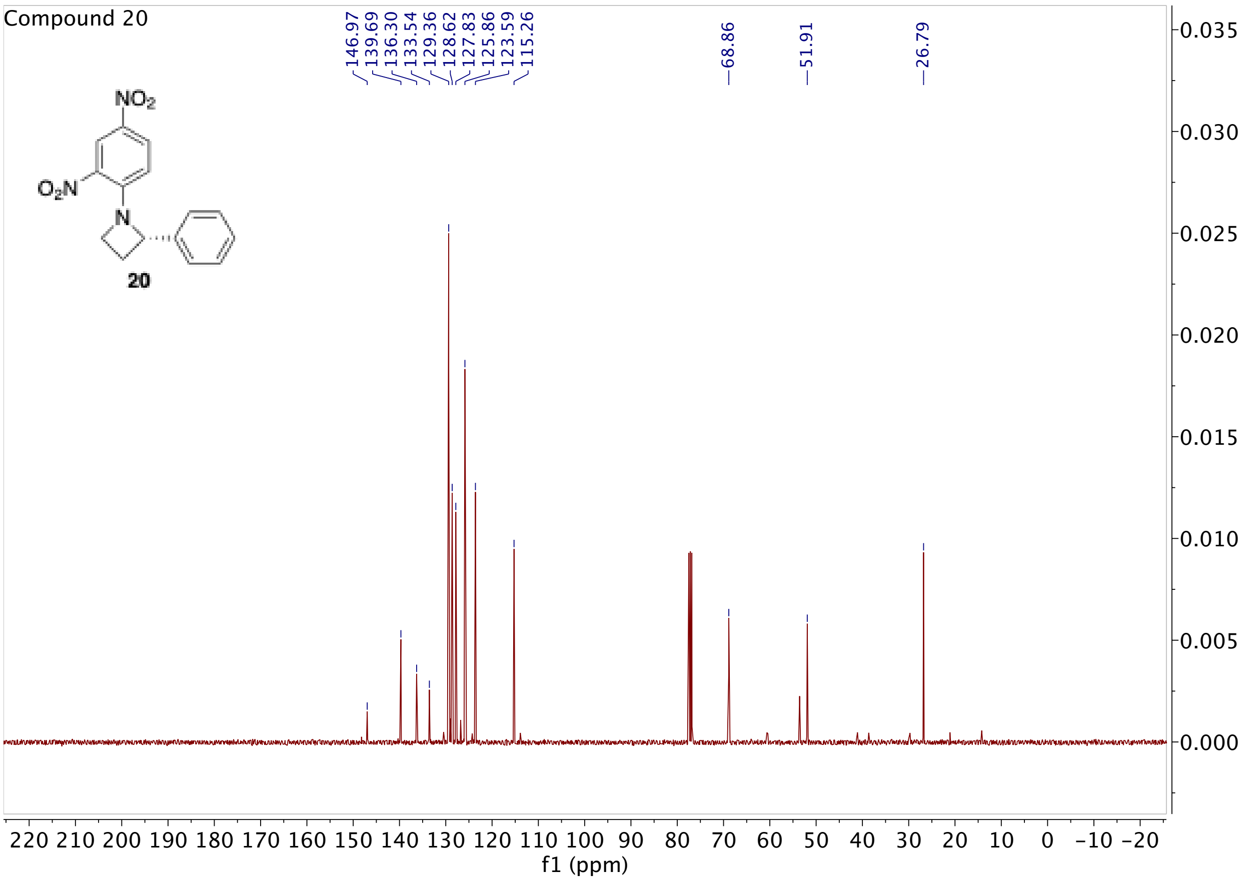

Acquired on a 400 MHz JEOL spectrometer.

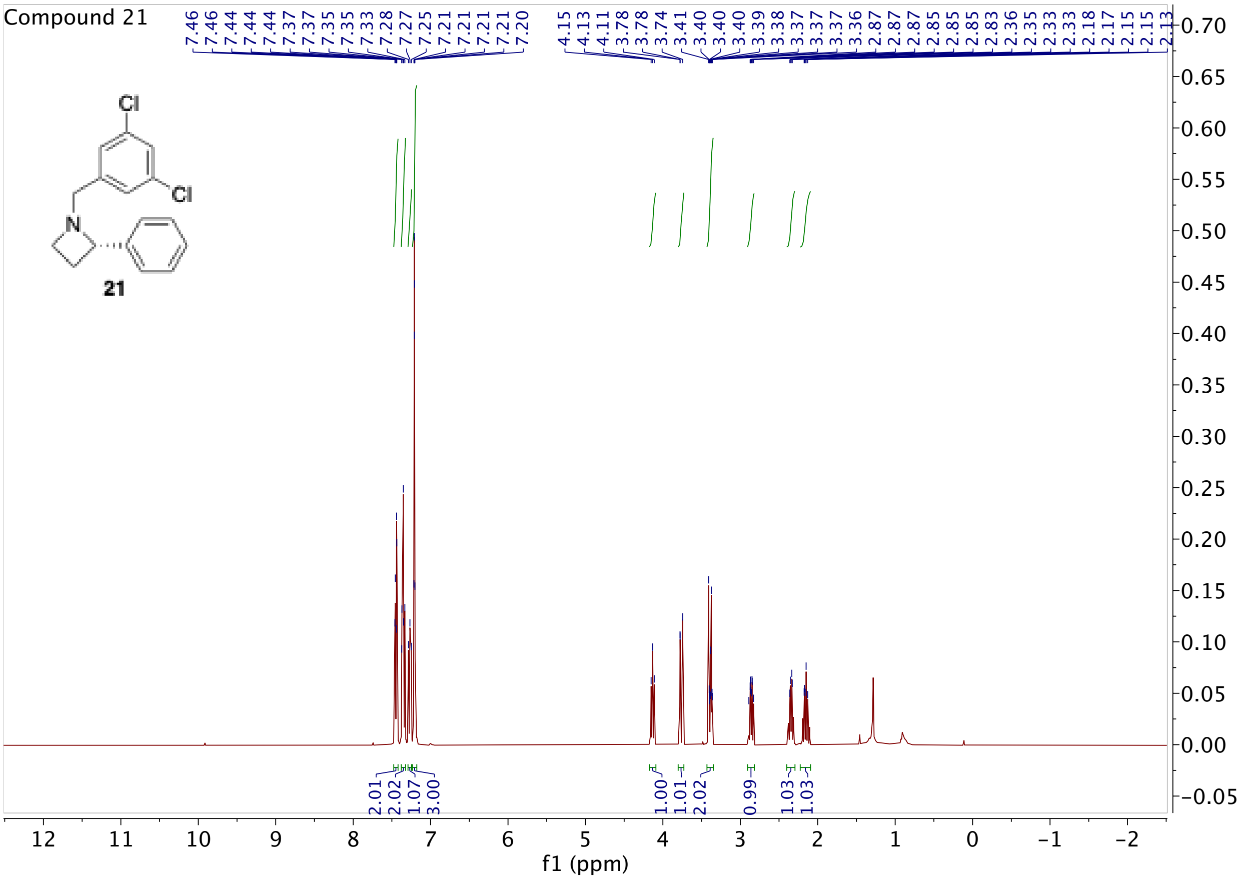

Acquired on a 400 MHz JEOL spectrometer.

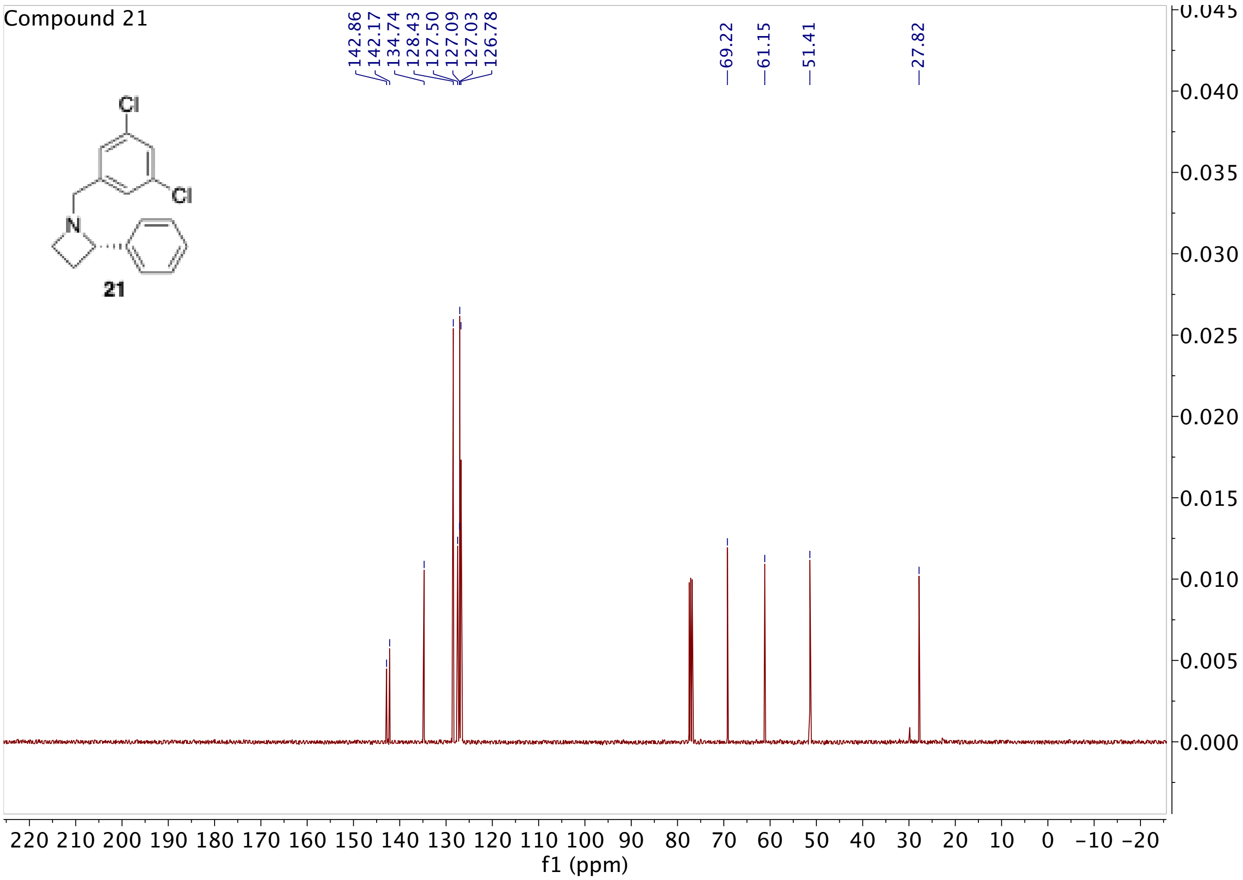

Acquired on a 400 MHz JEOL spectrometer.

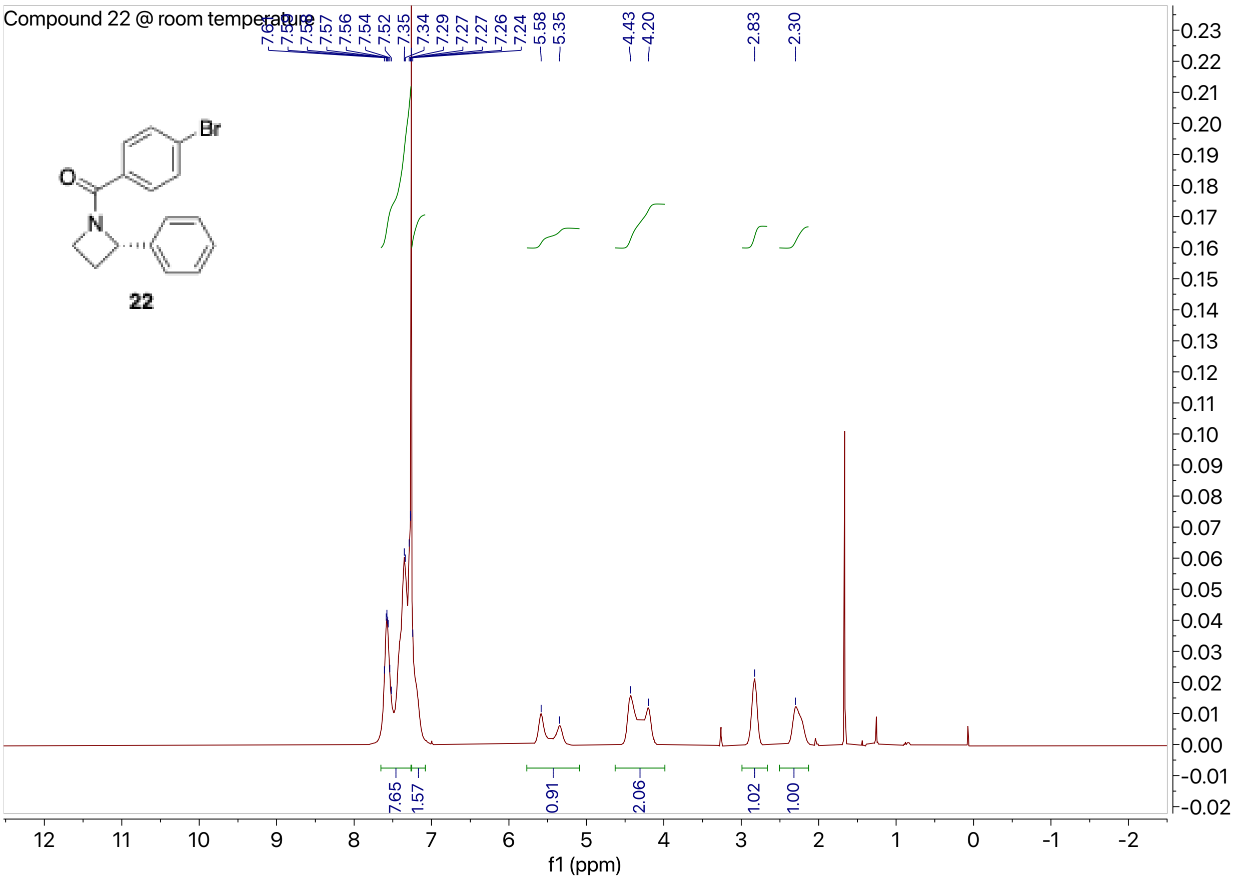

Acquired on a 400 MHz JEOL spectrometer.

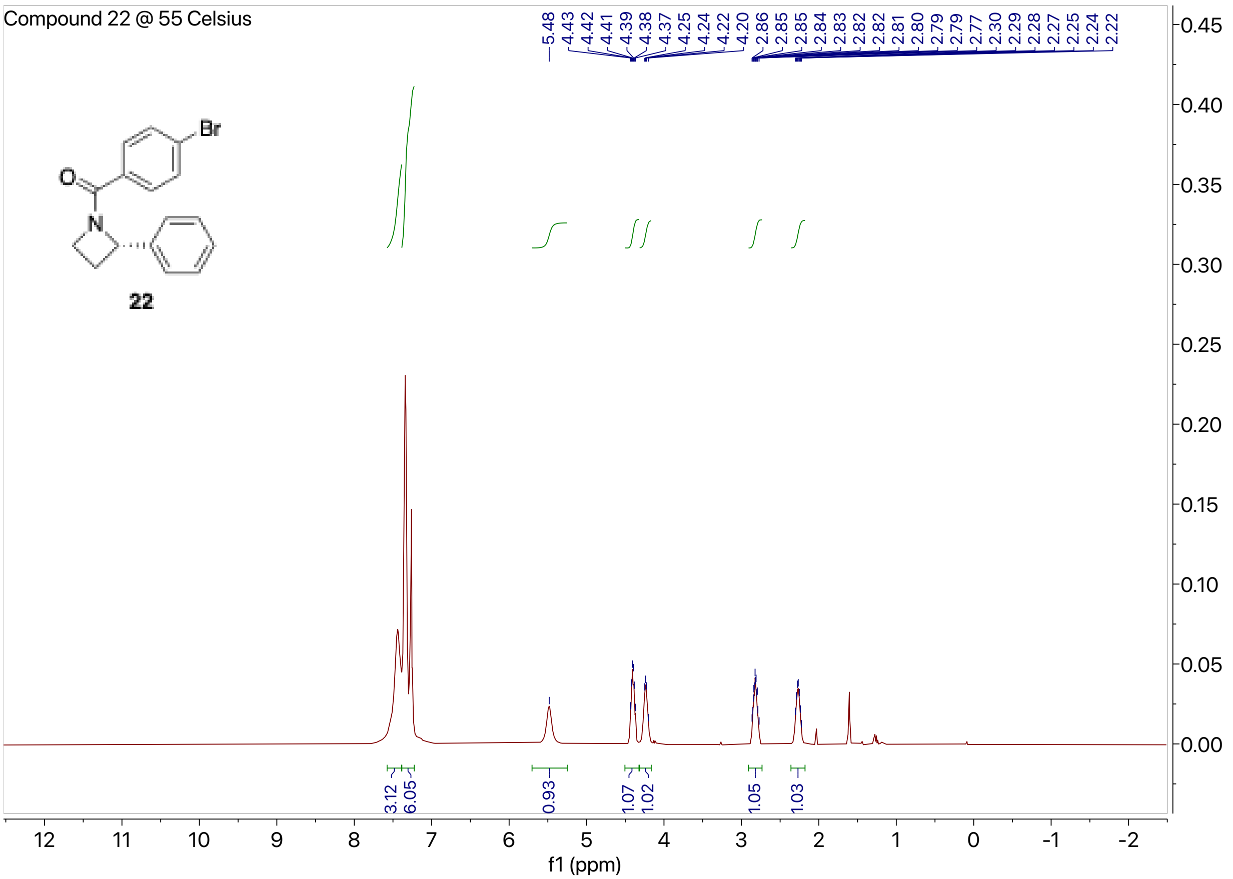

Acquired on a 400 MHz JEOL spectrometer.

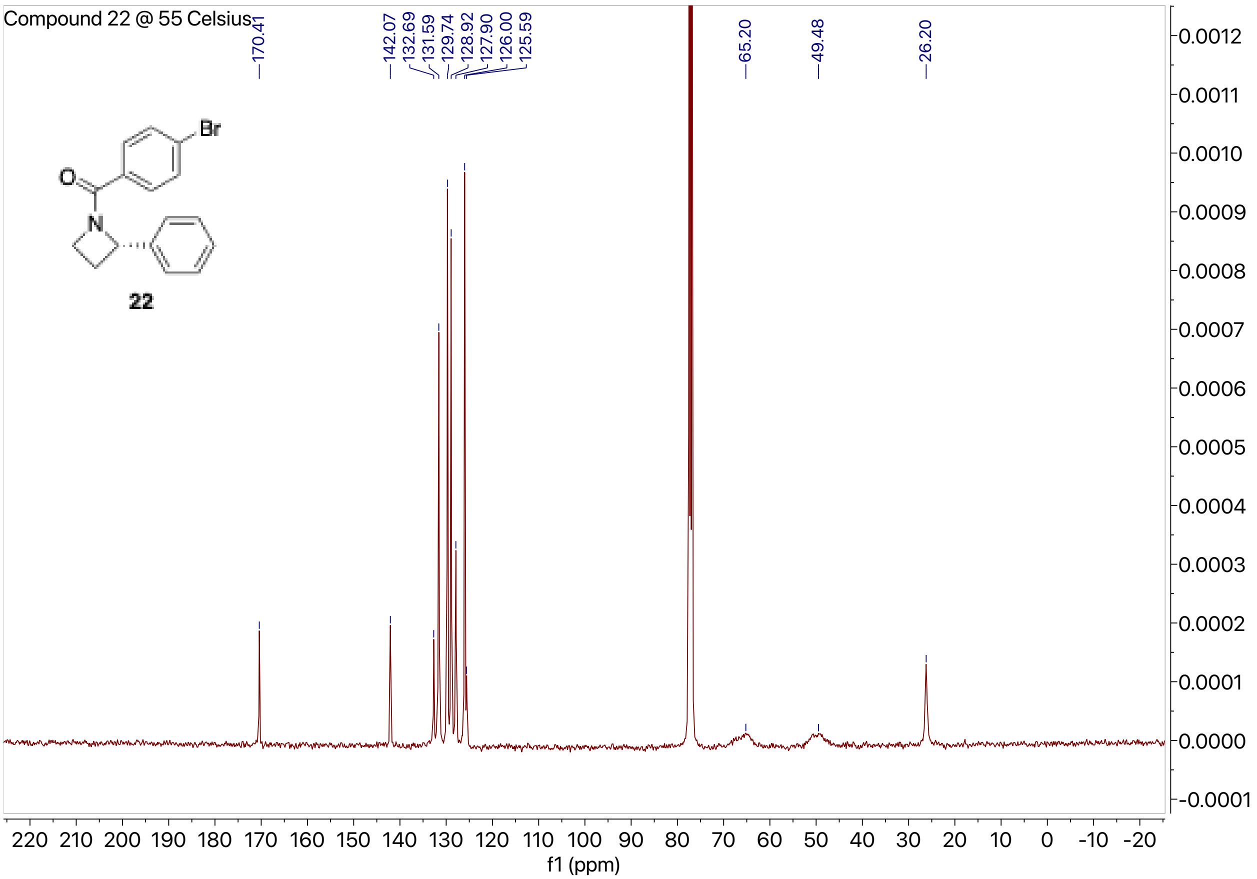

Acquired on a 400 MHz JEOL spectrometer.

Literature Cited:

(1) De Mico, A.; Margarita, R.; Parlanti, L.; Vescovi, A.; Piancatelli, G. A Versatile and Highly Selective Hypervalent Iodine (III)/2,2,6,6-Tetramethyl-1-Piperidinyloxyl-Mediated Oxidation of Alcohols to Carbonyl Compounds. *J. Org. Chem.* **1997**, *62* (20), 6974–6977. https://doi.org/10.1021/jo971046m.
(2) Fall, A.; Sene, M.; Gaye, M.; Gómez, G.; Fall, Y. Ionic Liquid-Supported TEMPO as Catalyst in the Oxidation of Alcohols to Aldehydes and Ketones. *Tetrahedron Lett.* **2010**, *51* (34), 4501–4504. https://doi.org/10.1016/j.tetlet.2010.06.086.
(3) Krasutsky, S. G.; Jacobo, S. H.; Tweedie, S. R.; Krishnamoorthy, R.; Filatov, A. S. Route Optimization and Synthesis of Taxadienone. *Org. Process Res. Dev.* **2015**, *19* (1), 284–289. https://doi.org/10.1021/op500314c.
(4) Ashley, G. W.; Burlingame, M.; Desai, R.; Fu, H.; Leaf, T.; Licari, P. J.; Tran, C.; Abbanat, D.; Bush, K.; Macielag, M. Preparation of Erythromycin Analogs Having Functional Groups at C-15. *J. Antibiot. (Tokyo)* **2006**, *59* (7), 392–401. https://doi.org/10.1038/ja.2006.56.
(5) Liu, G.; Cogan, D. A.; Ellman, J. A. Catalytic Asymmetric Synthesis of Tert-Butanesulfinamide. Application to the Asymmetric Synthesis of Amines. *J. Am. Chem. Soc.* **1997**, *119* (41), 9913–9914. https://doi.org/10.1021/ja972012z.
(6) Liu, G.; Cogan, D. A.; Owens, T. D.; Tang, T. P.; Ellman, J. A. Synthesis of Enantiomerically Pure N-Tert-Butanesulfinyl Imines (Tert-Butanesulfinimines) by the Direct Condensation of Tert-Butanesulfinamide with Aldehydes and Ketones. *J. Org. Chem.* **1999**, *64* (4), 1278–1284. https://doi.org/10.1021/jo982059i.
(7) Robak, M. T.; Herbage, M. A.; Ellman, J. A. Synthesis and Applications of Tert-Butanesulfinamide. *Chem. Rev.* **2010**, *110* (6), 3600–3740. https://doi.org/10.1021/cr900382t.
(8) Cogan, D. A.; Liu, G.; Ellman, J. Asymmetric Synthesis of Chiral Amines by Highly Diastereoselective 1,2-Additions of Organometallic Reagents to N-Tert-Butanesulfinyl Imines. *Tetrahedron* **1999**, *55* (29), 8883–8904. https://doi.org/10.1016/S0040-4020(99)00451-2.
